# Iron‐Catalyzed Borylation of Propargylic Acetates for the Synthesis of Multisubstituted Allenylboronates

**DOI:** 10.1002/chem.202203130

**Published:** 2022-11-22

**Authors:** Aitor Bermejo‐López, Wei‐Jun Kong, Pedro J. Tortajada, Daniels Posevins, Belén Martín‐Matute, Jan‐E. Bäckvall

**Affiliations:** ^1^ Department of Organic Chemistry Arrhenius Laboratory Stockholm University 10691 Stockholm Sweden

**Keywords:** Allenes, allenylboronates, borylation, iron, propargyl esters

## Abstract

A novel iron‐catalyzed borylation of propargylic acetates leading to allenylboronates has been developed. The method allows the preparation of a variety of di‐, tri‐ and tetrasubstituted allenylboronates at room temperature with good functional group compatibility. Stereochemical studies show that an *anti*‐S_N_2’ displacement of acetate by boron occurs; this also allows transfer of chirality to yield enantiomerically enriched allenylboronates. The synthetic utility of this protocol was further substantiated by transformations of the obtained allenylboronates including oxidation and propargylation.

## Graphical abstract

**Multisubstituted allenylboronates** were easily accessed through iron‐catalyzed borylation of propargylic acetates. The reaction is highly efficient, occurs under mild conditions, and is easy to scale up. The reaction is stereospecific and occurs with *anti*‐S_N_2‘ displacement of the acetate by boron, which allows a transfer of chirality. Several transformations including propargylation and further additions highlight the synthetic utility of this reaction.

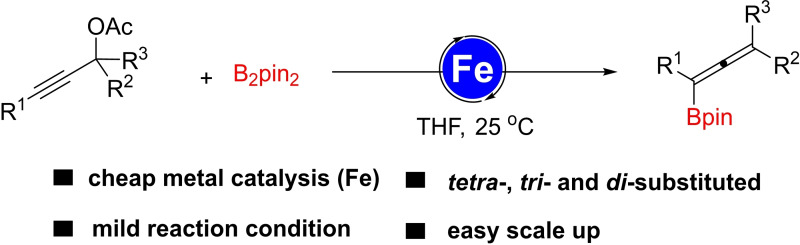

## Introduction

**Scheme 1:**
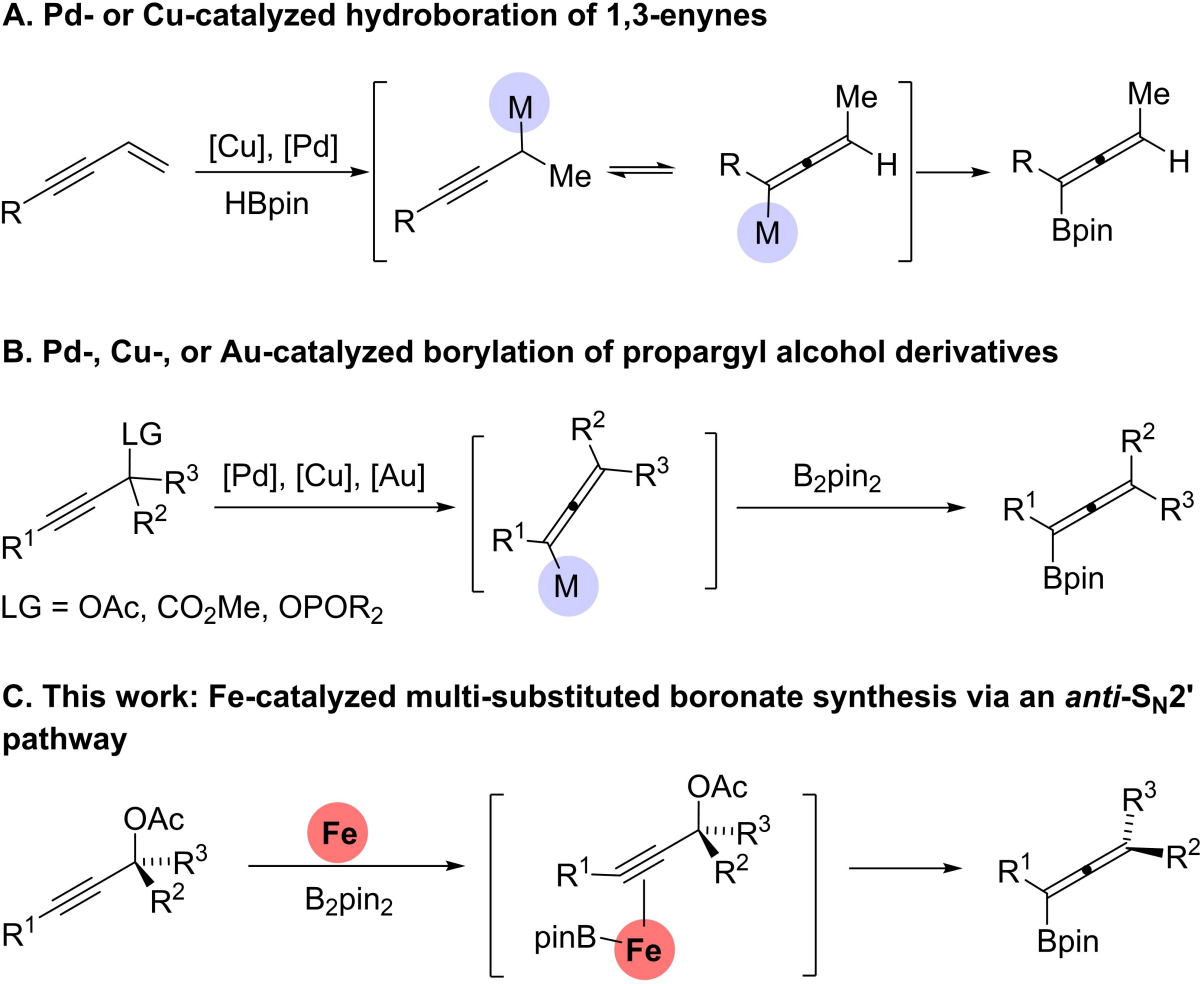
Previous work on allenylboronate synthesis and this work.

Organoboron compounds are valuable building blocks in organic synthesis and are widely applied in reactions such as cross‐coupling and nucleophilic addition.^[1]^ Allenylboronates have proven to be useful reagents in propargylation^[2]^ and allenylation^[3]^ reactions. Currently, there are two main approaches to the synthesis of allenylboronates: one employs a copper‐ or palladium‐catalyzed hydroboration of 1,3‐enynes usually affording trisubstituted allenylboronates (Scheme 1A).^[4]^ The other approach involves palladium‐, copper‐, or gold‐catalyzed borylation of propargyl alcohol derivatives (Scheme 1B).^[5]^ Additionally, a recent study reported allenylboronate synthesis through reductive cleavage of the propargylic C−O bond with sodium dispersion, followed by treatment with trimethoxyborane.^[6]^ However, stoichiometric amounts of a reactive alkali metal are applied at low temperature in this protocol, and this limits the practical use of the method. In spite of these advances, research on the synthesis and applications of allenylboronates still lags behind other organoboron compounds, hence the development of efficient and scalable methods for allenylboronate synthesis is still in high demand.

Iron catalysis has attracted increasing attention over the past decades due to the high abundance, low cost, and low toxicity of iron.^[7]^ While iron‐catalyzed C−C bond‐forming cross‐couplings have been extensively explored, the C−B formation through the coupling of an electrophile with a boron reagent has met with limited success. In 2014, the groups of Cook^[8]^ and Bedford^[9]^ independently reported an elegant iron‐catalyzed borylation of alkyl halides using ethyl magnesium bromide and *tert*‐butyl lithium as activators, respectively. Later on, Nakamura,^[10]^ Qu^[11]^ and Feng^[12]^ have further developed iron‐catalyzed borylation of different electrophiles including aryl halides, allylic esters and alkyl chlorides with *tert*‐butoxides as additives at elevated temperatures. Radical pathways were suggested in these reactions,^[12a]^ which occur in a non‐stereoselective manner. In previous studies, the groups of Fürstner and Bäckvall have explored the versatility of iron catalysis in allene synthesis through the coupling of propargyl alcohol derivatives and Grignard reagents.^[13]^ Notably, these iron‐catalyzed reactions proceeded predominately through a *syn*‐S_N_2’ pathway. To the best of our knowledge no examples of iron‐catalyzed borylation of propargylic derivatives have yet been reported. Herein, we report an unprecedented iron‐catalyzed borylation of propargylic acetates for the synthesis of allenylboronates using inexpensive iron(III) acetoacetate (Fe(acac)_3_) as precatalyst (Scheme 1C). The reaction provides access to a variety of di‐, tri‐, and tetrasubstituted allenylboronates under mild reaction conditions and is the first example on an iron‐catalyzed borylation of propargylic alcohol derivatives to give allenylboronates. The synthetic utility of the iron‐catalyzed borylation was demonstrated by gram‐scale reactions with subsequent transformations of the allenylboronate products. The borylation reaction was also found to be stereospecific, occurring preferentially according to an *anti*‐S_N_2’ pathway, which allowed a chirality transfer.

## Results and Discussion

We began our studies by running the reaction of propargyl acetate **1 a** as substrate with bis(pinacolato)diboron (B_2_pin_2_) as boron reagent in the presence of Fe(acac)_3_ (10 mol%) and tetramethylethylenediamine (TMEDA, 10 mol%) with EtMgBr as activator at room temperature. To our delight, the desired allenylboronate **2 a** was obtained in 74 % yield (Table 1, entry 1). Propargyl carbonate (**1 aa**) and pivalate (**1 ab**) are also feasible substrates in this reaction, giving **2 a** in 65 and 68 % yield, respectively (entries 2 and 3). However, when methylsulfonate (**1 ac**) and methyl ether (**1 ad**) were applied as leaving groups, low yields of **2 a** were observed (entries 4 and 5), while free alcohol (**1 ae**) failed to deliver the desired product **2 a** (entry 6). Elevating the reaction temperature to 50 °C with acetate **1 a** led to full conversion of the starting material but with low yield of **2 a** (50 %, entry 7). The use of *N*‐heterocyclic carbene 1,3‐dimesitylimidazolium chloride (IMes ⋅ HCl) as ligand in place of TMEDA resulted in an inferior yield of **2 a** (entry 8 vs. entry 1). Control experiment confirmed the necessity of the iron catalyst and TMEDA (entry 9). The yield of **2 a** increased to 85 % when the amounts of Fe(acac)_3_ and TMEDA were both reduced to 1.0 mol% (entry 10). When toluene and dimethoxyethane were used as solvents, less than 10 % yield of **2 a** was obtained (entries 11 and 12). Iron(II) salts Fe(OAc)_2_ and FeBr_2_ gave **2 a** in only 21 % and 18 % yield, respectively (entries 13 and 14). Reducing the amount of EtMgBr from 2.5 to 1.25 equivalents (with 1 mol% of Fe(acac)_3_) further increased the yield to 98 % (entry 15). It was confirmed that TMEDA is required for obtaining a good yield, since without TMEDA the yield dropped to 52 % (entry 16 vs. entry 15). The use of PhMgBr in place of EtMgBr afforded **2 a** in only 27 % yield (entry 17), while replacing EtMgBr by potassium *tert*‐butoxide (KO*t*Bu) or lithium methoxide (LiOMe) failed to give the desired product **2 a** (entries 18 and 19).

**Table 1:** Optimization of the iron‐catalyzed borylation of propargyl alcohol derivatives to allenylboronates.^[a]^

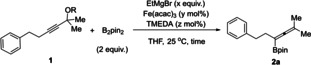						
	R	*x*	*y*/*z*	*t* [h]	**1** [%]	**2 a** [%]^[a]^
1	Ac (**1 a**)	2.5	10/10	16	25	74
2	CO_2_Me (**1 aa**)	2.5	10/10	16	5	65
3	Piv (**1 ab**)	2.5	10/10	21	15	68
4	Ms (**1 ac**)	2.5	10/10	18	11	27
5	Me (**1 ad)**	2.5	10/10	16	54	10
6	H (**1 ae**)	2.5	10/10	16	46	–
7^[b]^	Ac (**1 a**)	2.5	10/10	16	–	50
8^[c]^	Ac (**1 a**)	2.5	10/–	18	10	60
9	Ac (**1 a**)	2.5	–/–	16	77	14
10	Ac (**1 a**)	2.5	1/1	17	3	85
11^[d]^	Ac (**1 a**)	2.5	1/1	16	–	<10
12^[e]^	Ac (**1 a**)	2.5	1/1	18	–	<10
13^[f]^	Ac (**1 a**)	2.5	–/1	18	57	21
14^[g]^	Ac (**1 a**)	2.5	–/1	18	55	18
**15**	**Ac (1 a)**	**1.25**	**1/1**	**18**	–	**95(88)**
16	Ac (**1 a**)	1.25	1/–	18	–	52
17^[h]^	Ac (**1 a**)	–	10/10	18	25	27
18^[i]^	Ac (**1 a**)	–	10/10	18	50	–
19^[j]^	Ac (**1 a**)	–	10/10	18	41	–

[a] Yields were determined by using 1,3,5‐trimethoxylbenzene as internal standard; isolated yields in parenthesis. [b] At 50 °C. [c] IMes⋅HCl (10 mol%) was added instead of TMEDA. [d] toluene as solvent. [e] Dimethoxyethane (DME) as solvent. [f] Fe(OAc)_2_ as catalyst precursor. [g] FeBr_2_ as catalyst precursor. [h] PhMgBr (1.5 equiv.) in place of EtMgBr. [i] KO*t*Bu (1.5 equiv.) in place of EtMgBr. [j] LiOMe (1.5 equiv.) in place of EtMgBr.

**Scheme 2:**
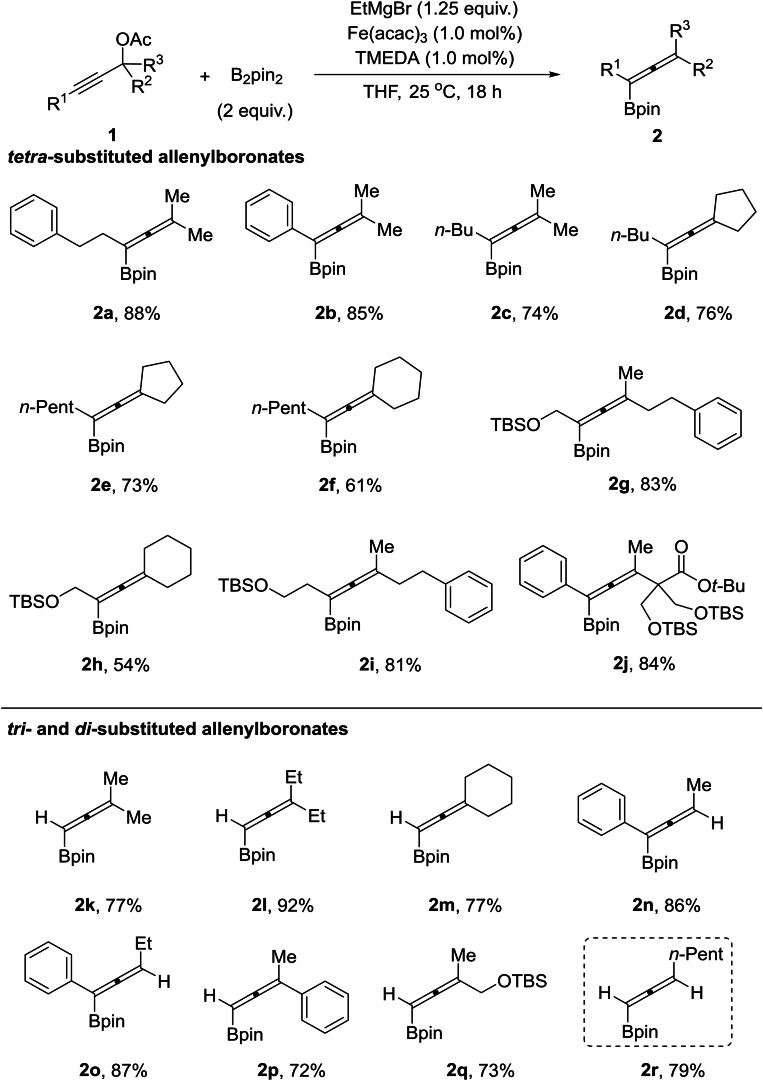
Scope of the iron‐catalyzed borylation reaction. Isolated yields.

After establishing the optimal reaction conditions for the iron‐catalyzed borylation, we explored its versatility with various propargyl acetates **1** (Scheme 2). First, the scope of **1** that could be transformed to tetrasubstituted allenylboronate was tested. Simple alkyl‐substituted propargyl acetates **1 a**–**1 f** including tertiary cyclopentyl (**1 d** and **1 e**) and cyclohexyl (**1 f**) acetates afforded the corresponding products **2 a**–**2 f** in good to excellent yields. This protocol also provided expedient access to *tert*‐butyldimethylsilyl (TBS)‐protected α‐ and β‐hydroxy allenylboronates **2 g**–**2 i**. Notably, the protected hydroxy group of allenylboronate provides a handle for various potential further transformations.^[14]^ Allenylboronate **2j** bearing a sterically hindered α‐all‐carbon quaternary center was obtained in 84 % yield with the tolerance of an ester group.

Then, the reaction towards synthesis of trisubstituted allenylboronates was evaluated. 3,3’‐Dimethyl, diethyl and cyclohexyl allenylboronates **2 k**–**2 m** were synthesized in good to excellent yields from the corresponding propargylic acetates. The reaction shows compatibility with aryl groups as shown in the synthesis of trisubstituted allenylboronates **2 n**–**2 p** containing a phenyl group. A TBS protected α‐hydroxyl trisubstituted allenylboronates **2 q** is accessed in good yield as well. It is worth noting that the reaction also produced disubstituted allenylboronate, as exemplified by the synthesis of **2 r** in 79 % yield.

**Scheme 3:**
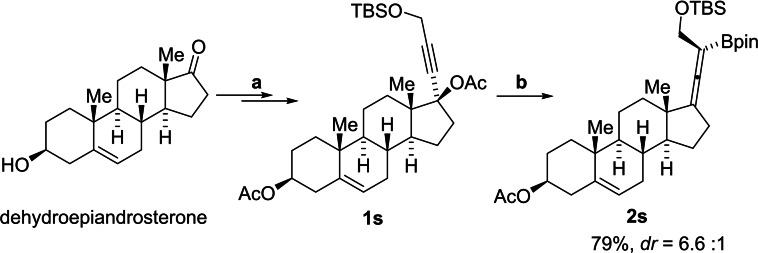
Functionalization of dehydroepiandrosterone. [a] See the Supporting Information. [b] Fe(acac)_3_ (1.0 mol%), **1 s** (0.3 mmol, 1 equiv.), B_2_pin_2_ (0.6 mmol, 2 equiv.), EtMgBr (0.38 mmol, 1.25 equiv.), THF (1.5 mL).

We also applied the iron‐catalyzed borylation to the synthesis of dehydroepiandrosterone‐derived allenylboronate **2 s** from the corresponding propargyl ester **1 s**, which successfully delivered the desired product **2 s** in 79 % yield in a 6.6 : 1 diastereoisomeric ratio. The major stereoisomer was formed by *anti*‐S_N_2’ displacement of the acetate group by the boron (Scheme 3).

**Scheme 4:**
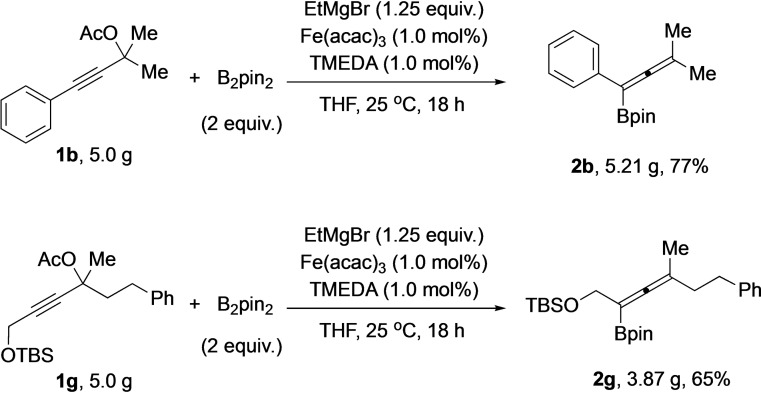
Gram‐scale synthesis of allenylboronates **2 b** and **2 g**.

The practical utility of the iron‐catalyzed allenylboronate synthesis approach was demonstrated by the easy scale up. Thus, from 5.0 g of staring material **1 b**, 5.21 g (77 % yield) of **2 b** was obtained. Furthermore, the scale up was applied to a more complex substrate **1 g**, which afforded 3.87 g (65 % yield) of product **2 g** (Scheme 4).

**Scheme 5:**
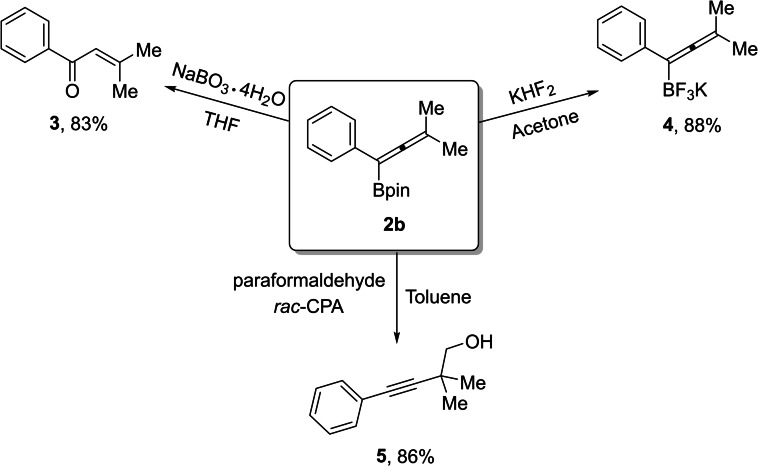
Synthetic applications of allenylboronate **2 b**. Yields shown are isolated yields.

Allenylboronates are highly valuable building blocks in organic synthesis that can be readily transformed to other functionalized compounds.^[15]^ A series of transformations were conducted using allenylboronate **2 b** (Scheme 5). Treatment of **2 b** with NaBO_3_⋅4 H_2_O yielded oxidized vinyl ketone product **3** in 83 % isolated yield. In a second selected application, trifluoroborate salt **4** was prepared upon addition of KHF_2_ in acetone. Finally, addition of paraformaldehyde in the presence of a racemic phosphoric acid (see the Supporting Information) afforded product **5** in 86 % isolated yield (Scheme 5).

**Scheme 6:**
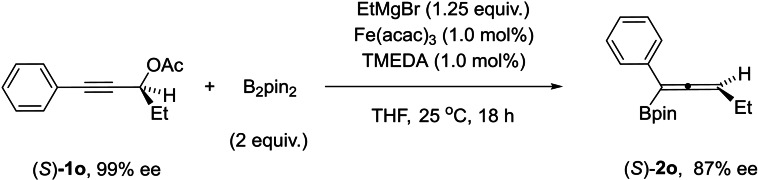
Chirality transfer experiment of allenylboronate (*S*)‐**1 o**.

The stereochemistry of the iron‐catalyzed borylation of (*S*)‐**1 o** to give (*S*)‐**2 o** revealed that the reaction proceeds with high level of chirality transfer (Scheme 6). The use of enantioenriched substrate (*S*)‐**1 o** (99 % *ee*) afforded product (*S*)‐**2 o** with an enantiomeric excess of 87 %. The absolute stereochemistry of (*S*)‐**2 o** was confirmed by comparison of its optical rotation value with the *R* enantiomer reported by Ge.^[4c]^ This result indicates that the reaction mainly operates according to an *anti*‐S_N_2’ pathway, in sharp contrast to our recent study, where iron‐catalyzed Grignard alkylation of propargylic ether occurred exclusively via a *syn*‐S_N_2’ pathway.^[13a]^

**Scheme 7:**
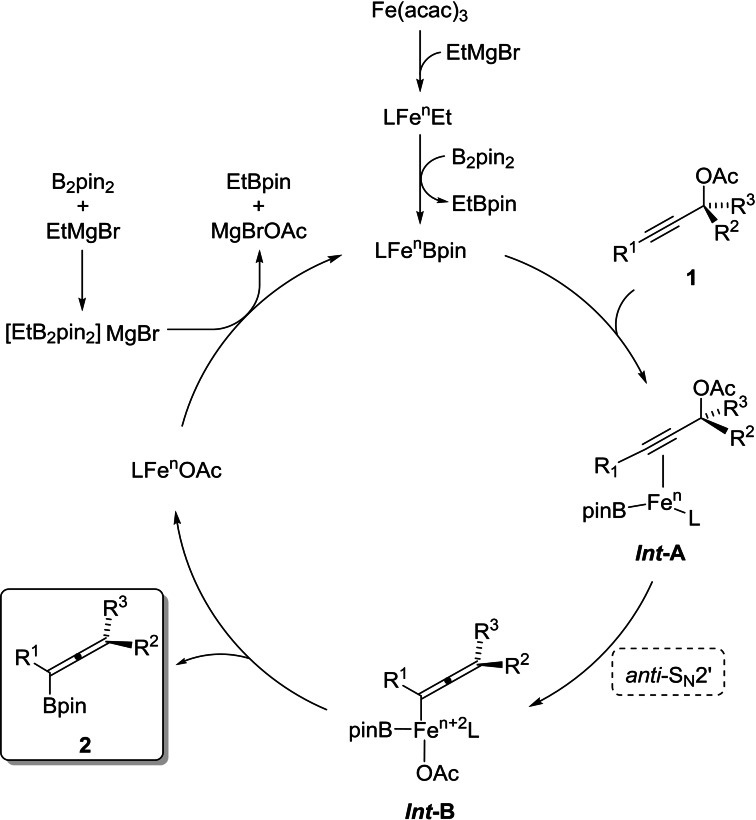
Proposed catalytic cycle.

A plausible mechanism for the formation of functionalized allenylboronates from propargylic acetates is given in Scheme 7. Based on previous studies,^[13]^ we propose initial formation of [LFe^*n*^Et] complexes resulting from the reaction between the Grignard reagent and Fe(acac)_3_,^[16]^ followed by transmetallation by B_2_pin_2_ forming a nucleophilic boron species, LFe^*n*^Bpin. The formation of these complexes is promoted by the presence of TMEDA and led to a color change from orange to dark brown.[^17^, ^18^] Coordination of the propargylic substrate **1** to the LFe^*n*^Bpin complex would give alkyne complex ***Int*** **‐A**. Oxidative addition by an *anti*‐S_N_2’ attack generates an allenyl iron intermediate ***Int*** **‐B**, which undergoes a reductive elimination affording allenylboronate products **2** (Scheme 7). It is not obvious why the reaction proceeds *syn* in our previous study (propargylic ethers)^[13a]^ and *anti* in the present study. One explanation could be that in the previous study,^[13a]^ where a Grignard alkyl is transferred, there would be an MgX group on iron that can coordinate the OMe leaving group, which would favor *syn* displacement. In the present study, where Bpin is bound to iron, there will be no MgX on iron, and furthermore the leaving group is now OAc (instead of OMe).

## Conclusion

In summary, we have reported a novel iron‐catalyzed borylation of readily accessible propargylic acetates, with a high level of chirality transfer, leading to multisubstituted allenylboronates. For the first time, iron catalysis has been used for this important transformation. Stereochemical studies showed that the borylation occurs with an *anti‐*S_N_2’ displacement of the acetate. The use of an inexpensive and environmentally friendly iron salt as catalyst under mild reaction conditions is an important advance in allenyl boronate synthesis. Furthermore, the reaction allows easy scale up and shows good functional group tolerance. The synthetic utility of the developed iron‐catalyzed allenylboronate synthesis has been further demonstrated by a series of transformations including oxidation and propargylation.

## Experimental Section

### Synthesis of multisubstituted allenylboronates

*General procedure*: Catalyst Fe(acac)_3_ (1.0 mol%) and B_2_pin_2_ (2.00 equiv.) were dissolved in a solution of TMEDA (1.0 mol%) in anhydrous THF (1.5 mL) in a dry 5 mL microwave flask and under argon. The resulting orange solution was cooled to 0 °C and the corresponding Grignard reagent (1.25 equiv.) was added, which led to a change in the color of the mixture from orange to dark brown. Then, the propargyl ester (0.30 mmol, 1.00 equiv.) was added dropwise. The reaction mixture was allowed to reach room temperature and was left for stirring for 16 h. After that time, the crude mixture was purified by flash column chromatography over SiO_2_ (eluent: pentane/diethyl ether) affording the desired product **2**.

*4,4,5,5‐Tetramethyl‐2‐(5‐methyl‐1‐phenylhexa‐3,4‐dien‐3‐yl)‐1,3,2‐dioxaborolane* (**2 a**): Following general procedure: Fe(acac)_3_ (1 mg, 0.003 mmol, 1.0 mol%), substrate **1 a** (69 mg, 0.30 mmol, 1.00 equiv.), B_2_pin_2_ (152 mg, 0.60 mmol, 2.00 equiv.) and ethylmagnesium bromide (3.0 M in diethyl ether, 125 μL, 0.38 mmol, 1.25 equiv.) in anhydrous THF (1.5 mL) were allowed to react. The reaction mixture was purified over SiO_2_ (pentane/diethyl ether 98 : 2) to yield product **2 a** (75 mg, 88 %) as a colorless oil, whose spectra match those in a literature report.^[5d]^ ^1^H NMR (400 MHz, CDCl_3_): *δ*=7.29–7.11 (m, 5 H), 2.74–2.68 (m, 2 H), 2.36–2.30 (m, 2 H), 1.62 (s, 6 H), 1.25 (s, 12 H); ^13^C NMR (101 MHz, CDCl_3_): *δ*=211.0, 142.8, 128.8, 128.2, 125.6, 91.7, 83.3, 35.8, 32.3, 24.9, 20.0. Carbons directly attached to boron atoms were not detected, most likely due to quadrupolar relaxation. HRMS (ESI): *m*/*z* calcd. for C_19_H_27_BNaO_2_: 321.200 [*M*+Na]^+^; found: 321.1968.

## Conflict of interest

The authors declare no conflict of interest.

1

## Supporting information

As a service to our authors and readers, this journal provides supporting information supplied by the authors. Such materials are peer reviewed and may be re‐organized for online delivery, but are not copy‐edited or typeset. Technical support issues arising from supporting information (other than missing files) should be addressed to the authors.

Supporting Information

## Data Availability

Research data are not shared.
