# Supplementary material for: Iron‐Catalyzed Borylation of Propargylic Acetates for the Synthesis of Multisubstituted Allenylboronates
Source: Chemistry. 2022 Nov 22;29(3):e202203130. doi: 10.1002/chem.202203130 (PMC10099795; doi:10.1002/chem.202203130)
Supplement: Supplementary file 1 — Supporting Information [file CHEM-29-0-s001.pdf]

Chemistry–A European Journal

Supporting Information

Iron-Catalyzed Borylation of Propargylic Acetates for the Synthesis of Multisubstituted Allenylboronates

Aitor Bermejo-López, Wei-Jun Kong, Pedro J. Tortajada, Daniels Posevins,* Belén Martín-Matute,* and Jan-E. Bäckvall*

Supporting Information

TABLE OF CONTENTS

1. **GENERAL INFORMATION** **S2**

2. **IRON-CATALYZED SYNTHESIS OF ALLENES** **S3**

 2.1 PREPARATION OF STARTING MATERIALS S3
 2.2 SYNTHESIS AND CHARACTERIZATION OF COMPOUNDS S4
 2.3 SYNTHESIS OF MULTISUBSTITUTED ALLENYLBORONATES S12
 2.4 STUDY OF THE STEREOCHEMISTRY S23

3. **SCALE-UP SYNTHESIS OF ALLENYLBORONATES** **S255**

4. **SELECTED APPLICATIONS OF ALLENYLBORONATES** **S25**

5. **NMR SPECTRA** **S28**

6. **REFERENCES** **S69**

1. General information

Unless otherwise noted, all reagents were used as received from commercial suppliers. Dry solvents were obtained from commercial sources, from a VAC™ drying system or dried over molecular sieves. $Fe(acac)_3$ was purchased from Sigma-Aldrich (Prod. Nr.: 517003, Lot Nr.: MKBS7930V with a Cu content of 0.5 ppm). Grignard reagents were titrated before use.[1] All reactions were conducted in dry flasks under argon atmosphere. Room temperature is ca. 22 °C. Reactions were monitored using Merck silica gel 60 F254 plates (TLC analysis). TLC plates were visualized with UV light (254 nm) or $KMnO_4$. Flash column chromatography was carried out with 60Å (particle size 35 - 70 µm) silica gel. ^{1}H-/^{13}C-NMR experiments were performed on a Bruker NMR (400/101 MHz) or (500/125 MHz) at room temperature. ^{19}F-NMR experiments were performed on a 400 MHz Bruker NMR (377 MHz). Chemical shifts (δ) are reported in parts per million (ppm) relative to the $CDCl_3$ peak (δ(H) = 7.26 and δ(C) = 77.16 ppm). Coupling constants (*J*) are reported in Hertz (Hz). HRMS were recorded on a Bruker MicroTOF spectrometer equipped with an ESI or APCI as ion sources. The enantiomeric excess of compounds was determined by chiral GC using racemic compounds as references. GC analyses were performed using the following instruments: a) Varian GC 3900 using an IVADEX-1 chiral column from IVA Analysentechnik with FID detector and N_2 as a carrier gas with a flow of 1.8 mL/min; b) Agilent 8860 GC System using an Hydrodex β-DM chiral column with 5977B GC/MSD detector and N_2 as a carrier gas with a flow of 1.8 mL/min.

2. Iron-Catalyzed Synthesis of Allenes

2.1 Preparation of starting materials

General procedure A. Preparation of substrates from Table 1 entries 1-6:

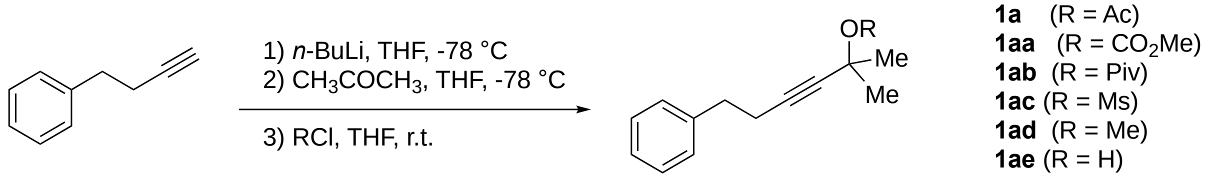

In accordance to the reported procedure,[2] *n*-BuLi (2.5 M solution in THF, 2.5 mL, 6.25 mmol, 1.25 equiv.) under Ar atmosphere was added to anhydrous THF (10.0 mL) at –78 °C. Then, 4-phenyl-1-butyne (651 mg, 5.00 mmol, 1.00 equiv.) was added dropwise. After 30 minutes at – 78 °C, acetone (558 *µ*L, 7.50 mmol, 1.50 equiv.) in anhydrous THF (2.0 mL) was added dropwise. The reaction mixture was allowed to warm to room temperature and stirred for 2 hours. The reaction mixture was cooled to 0 °C and the corresponding RCl reagent (10.00 mmol, 2.00 equiv.) was added dropwise. The mixture was allowed to reach room temperature and stirred overnight. After completion, sat. aq. NH_4Cl was added and the mixture was extracted with diethyl ether, washed with sat. aq. $NaHCO_3$, water and dried over Na_2SO_4. The crude product was purified over SiO_2 (ethyl acetate/pentane) to afford the corresponding alcohol esters **1a-1ae** as products.

General procedure B. Preparation of propargyl acetates 1b-1r:

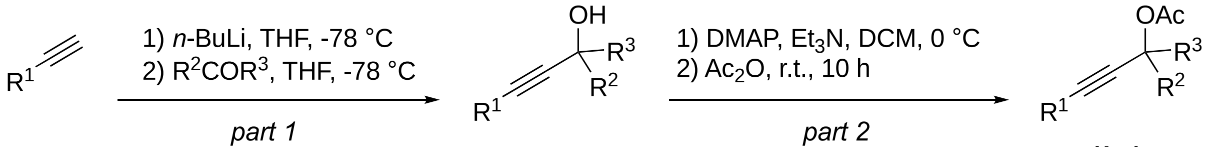

1b-1r

n-BuLi (1.10 equiv.) was added to a solution of alkyne (1.00 equiv.) in THF (0.5 M) at -78 °C under Ar atmosphere. The mixture was stirred for 1 hour at -78 °C and the corresponding ketone (1.50 equiv.) was added. After the complete consumption of the alkyne (as monitored by TLC), the mixture was quenched with sat. aq. NH_4Cl solution and extracted with diethyl ether. Combined organic phases were washed with brine, dried over Na_2SO_4 and concentrated

in vacuo. Purification over SiO_2 (eluent: ethyl acetate/pentane) afforded the corresponding propargyl alcohols.

To a solution of the propargyl alcohol (1.00 equiv.) in dry DCM at 0 °C was slowly added DMAP (0.10 equiv.) and triethylamine (1.50 equiv.). After stirring the mixture at 0 °C for 5 min, acetic anhydride or acetyl chloride (1.50 equiv.) was added. The solution was allowed to reach room temperature and stirred overnight (reaction progress was monitored by TLC). The resulting mixture was carefully quenched with sat. aq. $NaHCO_3$ solution and extracted with pentane. The combined ether extracts were concentrated in vacuo and the crude product was purified over SiO_2 (ethyl acetate/pentane) to yield the desired propargyl acetyl esters as products.

2.2 Synthesis and characterization of compounds

2-Methyl-6-phenylhex-3-yn-2-yl acetate (**1a**)

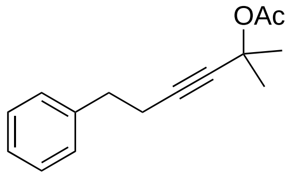

1a

Following general procedure A starting from 4-phenyl-1-butyne (5.0 mmol) and acetone, and using acetyl chloride. Purification over SiO_2 (ethyl acetate/pentane 5%) gave product as a colorless oil.

^{1}H NMR (400 MHz, $CDCl_3$) δ 7.31–7.18 (m, 5 H), 2.81 (t, *J* = 7.5 Hz, 2 H), 2.49 (t, *J* = 7.5 Hz, 2 H), 2.01 (s, 3 H), 1.61 (s, 6 H); ^{13}C NMR (101 MHz, $CDCl_3$) δ 169.6, 140.9, 128.7, 128.4, 126.3, 84.0, 82.3, 72.6, 35.1, 29.4, 22.2, 21.1; HRMS (ESI): m/z calcd. for $C_{15}H_{18}NaO_2$ $[M+Na]^+$: 253.1199; found: 253.1201.

2-Methyl-4-phenylbut-3-yn-2-yl acetate (**1b**)

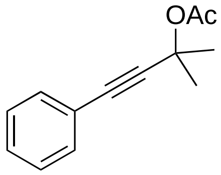

1b

Following general procedure B starting from ethynylbenzene (5.00 mmol) and acetone, and using acetyl chloride. Purification over SiO_2 (ethyl acetate/pentane 5%) gave product as a

colorless oil, whose spectra match literature report.[3]

^{1}H NMR (400 MHz, $CDCl_3$) δ 7.42–7.35 (m, 2 H), 7.21–7.18 (m, 3 H), 1.96–1.92 (m, 3 H), 1.69 (d, J = 2.1 Hz, 6 H); ^{13}C NMR (101 MHz, $CDCl_3$) δ 168.9, 131.6, 128.1, 128.0, 122.5, 106.4, 90.1, 83.8, 72.1, 28.8, 21.7; HRMS (ESI): calc. for $C_{13}H_{14}NaO_2$ $[M+Na]^+$: 225.0886; found: 225.0890.

2-Methyloct-3-yn-2-yl acetate (**1c**)

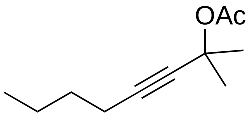

1c

Following general procedure B starting from hex-1-yne (5.00 mmol) and acetone, and using acetyl chloride. Purification over SiO_2 (ethyl acetate/pentane 5%) gave product as a colorless oil, whose spectra match literature report.[4]

^{1}H NMR (400 MHz, $CDCl_3$) δ 2.19 (t, J = 7.0 Hz, 2 H), 1.99 (s, 3 H), 1.62 (d, J = 1.7 Hz, 6 H), 1.51–1.42 (m, 2 H), 1.41–1.34 (m, 2 H), 0.89 (t, J = 7.3 Hz, 3 H); ^{13}C NMR (101 MHz, $CDCl_3$) δ 169.5, 84.8, 81.5, 72.8, 30.8, 29.4, 22.2, 22.0, 18.5, 13.7; HRMS (ESI): m/z calcd. for $C_{11}H_{18}NaO_2$ $[M+Na]^+$: 205.1199; found: 205.1199.

1-(Hex-1-yn-1-yl)cyclopentyl acetate (**1d**)

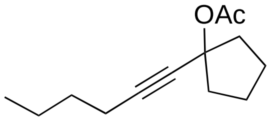

1d

Following general procedure B starting from hex-1-yne (5.00 mmol) and cyclopentanone. Purification over SiO_2 (ethyl acetate/pentane 5%) gave product as a colorless oil, whose spectra match literature report.[5]

^{1}H NMR (400 MHz, $CDCl_3$) δ 2.21–2.11 (m, 4 H), 2.08–2.02 (m, 2 H), 2.00 (s, 3 H), 2.75–1.67 (m, 4 H), 1.50–1.33 (m, 4 H), 0.88 (t, J = 7.3 Hz, 3 H); ^{13}C NMR (101 MHz, $CDCl_3$) δ 169.8, 85.4, 81.2, 80.7, 40.6, 30.8, 23.3, 22.0, 18.6, 13.7; HRMS (ESI): m/z calcd. for $C_{13}H_{20}NaO_2$ $[M+Na]^+$: 231.1356; found: 231.1360.

1-(Hept-1-yn-1-yl)cyclopentyl acetate (**1e**)

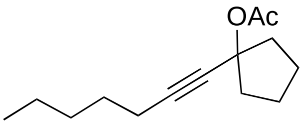

1e

Following general procedure B starting from hept-1-yne (5.00 mmol) cyclopentanone. Purification over SiO_2 (ethyl acetate/pentane 5%) gave product as a colorless oil.

^{1}H NMR (400 MHz, $CDCl_3$) δ 2.22–2.13 (m, 4 H), 2.12–2.03 (m, 2 H), 2.00 (s, 3 H), 1.76–1.67 (m, 4 H), 1.48 (p, *J* = 7.2 Hz, 2 H), 1.37–1.27 (m, 4 H), 0.88 (t, *J* = 6.9 Hz, 3 H); ^{13}C NMR (101 MHz, $CDCl_3$) δ 169.7, 85.4, 81.1, 80.7, 40.6, 31.1, 28.4, 23.3, 22.2, 22.0, 18.9, 14.1; HRMS (ESI): m/z calcd. for $C_{14}H_{22}NaO_2$ $[M+Na]^+$: 245.1512; found: 245.1519.

1-(Hept-1-yn-1-yl)cyclohexyl acetate (**1f**)

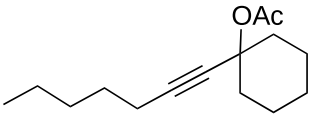

1f

Following general procedure B starting from hept-1-yne (5.00 mmol) and cyclohexanone. Purification over SiO_2 (ethyl acetate/pentane 5%) gave product as a colorless oil.

^{1}H NMR (400 MHz, $CDCl_3$) δ 2.22 (t, *J* = 7.1 Hz, 2 H), 2.08 (dt, *J* = 11.5, 5.0 Hz, 2 H), 2.02 (s, 3 H), 1.80 (dt, *J* = 13.3, 6.9 Hz, 2 H), 1.64–1.57 (m, 4 H), 1.57–1.55 (m, 3 H), 1.40–1.25 (m, 5 H), 0.89 (t, *J* = 6.9 Hz, 3 H); ^{13}C NMR (101 MHz, $CDCl_3$) δ 169.4, 87.0, 80.2, 76.2, 37.5, 31.2, 28.5, 25.4, 22.9, 22.3, 22.3, 18.9, 14.1; HRMS (ESI): m/z calcd. for $C_{15}H_{24}NaO_2$ $[M+Na]^+$: 259.1669; found: 259.1669.

6-((tert-Butyldimethylsilyl)oxy)-3-methyl-1-phenylhex-4-yn-3-yl acetate (**1g**)

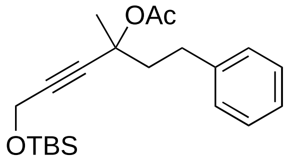

1g

Following general procedure B starting from *tert*-butyldimethyl(prop-2-yn-1-yloxy)silane (5.00 mmol) and 4-phenylbutan-2-one. Purification over SiO_2 (ethyl acetate/pentane 5%) gave product as a colorless oil.

^{1}H NMR (400 MHz, $CDCl_3$) δ 7.34–7.14 (m, 5 H), 4.39 (s, 2 H), 2.86–2.77 (m, 2 H), 2.30–2.22 (m, 1 H), 2.13–2.07 (m, 1 H), 2.00 (s, 3 H), 1.72 (s, 3 H), 0.92 (s, 9 H), 0.14 (d, *J* = 3.4

Hz, 6 H); ^{13}C NMR (101 MHz, $CDCl_3$) δ 169.3, 141.6, 128.5, 128.5, 126.0, 84.6, 84.3, 75.1, 51.9, 43.4, 30.8, 26.6, 25.9, 22.0, 18.3, -5.0 (x2); HRMS (ESI): m/z calcd. for $C_{21}H_{32}NaO_3Si$ $[M+Na]^+$: 383.2013; found: 383.2010.

1-(4-((tert-Butyldimethylsilyl)oxy)but-1-yn-1-yl)cyclohexyl acetate (**1h**)

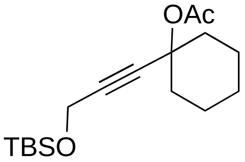

1h

Following general procedure B starting from *tert*-butyldimethyl(prop-2-yn-1-yloxy)silane 5.00 mmol) and cyclohexanone. Purification over SiO_2 (ethyl acetate/pentane 5%) gave product as a colorless oil, whose spectra match literature report.[4]

^1H NMR (400 MHz, $CDCl_3$) δ 4.34 (s, 2 H), 2.05 (dt, *J* = 12.2, 5.4 Hz, 2 H), 1.98 (s, 3 H), 1.83 (dt, *J* = 13.2, 6.7 Hz, 2 H), 1.61–1.53 (m, 4 H), 1.46 (dt, *J* = 14.7, 5.2 Hz, 1 H), 1.30 (dt, *J* = 13.2, 6.6 Hz, 1 H), 0.87 (s, 9 H), 0.09 (s, 6 H); ^{13}C NMR (101 MHz, $CDCl_3$) δ 169.2, 84.8, 84.7, 75.4, 51.9, 37.0, 25.9, 25.3, 22.6, 22.0, 18.3, -5.0; HRMS (ESI): m/z calcd. $C_{17}H_{30}NaO_3Si$ $[M+Na]^+$: 333.1856; found: 333.1862.

7-((tert-Butyldimethylsilyl)oxy)-3-methyl-1-phenylhept-4-yn-3-yl acetate (**1i**)

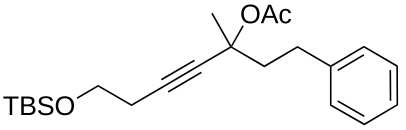

1i

Following general procedure B starting from (but-3-yn-1-yloxy)(tert-butyl)dimethylsilane (5.00 mmol) and 4-phenylbutan-2-one. Purification over SiO_2 (ethyl acetate/pentane 5%) gave product as a colorless oil.

^1H NMR (400 MHz, $CDCl_3$) δ 7.45–7.05 (m, 5 H), 3.78 (t, *J* = 7.1 Hz, 2 H), 2.85 (dd, *J* = 9.7, 7.3 Hz, 2 H), 2.52 (t, *J* = 7.1 Hz, 2 H), 2.34–2.25 (m, 1 H), 2.16–2.07 (m, 1 H), 2.04 (s, 3 H), 1.75 (s, 3 H), 0.94 (s, 9 H), 0.12 (s, 6 H); ^{13}C NMR (101 MHz, $CDCl_3$) δ 169.3, 141.7, 128.5, 128.4, 125.9, 83.1, 81.2, 75.5, 61.9, 43.6, 30.9, 26.9, 25.9, 23.3, 22.1, 18.3, -5.2; HRMS (ESI): m/z calcd. for $C_{22}H_{34}NaO_3Si$ $[M+Na]^+$: 397.2169; found: 397.2171.

tert-Butyl-3-acetoxy-2,2-bis(((tert-butyldimethylsilyl)oxy)methyl)-3-methyl-5-phenylpent-4-ynoate (**1j**)

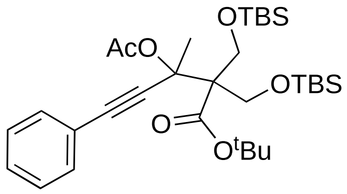

1j

Following general procedure B starting from ethynylbenzene (5.00 mmol) and *tert*-butyl 2,2-bis(((*tert*-butyldimethylsilyl)oxy)methyl)-3-oxobutanoate. Purification over SiO_2 (ethyl acetate/pentane 5%) gave product as a colorless oil.

^{1}H NMR (400 MHz, $CDCl_3$) δ 7.47–7.21 (m, 5 H), 4.22–4.05 (m, 4 H), 2.02 (s, 3 H), 1.94 (s, 3 H), 1.43 (s, 9 H), 0.89 (d, *J* = 4.3 Hz, 18 H), 0.11–0.04 (m, 12 H); ^{13}C NMR (101 MHz, $CDCl_3$) δ 169.6, 168.6, 131.8, 128.4, 128.3, 123.1, 87.9, 87.5, 80.8, 77.7, 61.3, 60.3, 28.2, 26.1 (x2), 24.3, 22.3, 18.4, 18.3, -5.4 (x2), -5.5; HRMS (ESI): m/z calcd. for $C_{32}H_{54}NaO_6Si_2$ $[M+Na]^+$: 613.3351; found: 613.3357.

2-Methylbut-3-yn-2-yl acetate (**1k**)

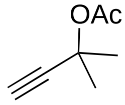

1k

Following part 2 of general procedure B starting from commercially available 2-methylbut-3-yn-2-ol (5.00 mmol). Purification over SiO_2 (ethyl acetate/pentane 5%) gave product as a colorless oil, whose spectra match literature report.[6]

^{1}H NMR (400 MHz, $CDCl_3$) δ 2.52 (s, 1 H), 2.02 (s, 3 H), 1.66 (s, 6 H); ^{13}C NMR (101 MHz, $CDCl_3$) δ 169.4, 84.7, 72.3, 71.6, 28.9, 21.9; HRMS (ESI): m/z calcd. for $C_7H_{10}NaO_2$ $[M+Na]^+$: 149.0573; found: 149.0576.

3-Ethylpent-1-yn-3-yl acetate (**1l**)

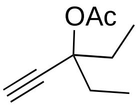

1l

Following part 2 of general procedure B starting from commercially available 3-ethyl-1-pentyn-3-ol (5.00 mmol). Purification over SiO_2 (ethyl acetate/pentane 5%) gave product as a colorless oil.

^{1}H NMR (400 MHz, $CDCl_3$) δ 2.55 (s, 1 H), 2.08–1.99 (m, 5 H), 1.96–1.87 (dq, J = 14.4, 7.4 Hz, 2 H), 0.98 (t, J = 7.4 Hz, 6 H); ^{13}C NMR (101 MHz, $CDCl_3$) δ 169.5, 83.1, 79.5, 74.1, 30.7, 22.0, 8.3; HRMS (ESI): m/z calcd. for $C_9H_{14}NaO_2$ $[M+Na]^+$: 177,0886; found: 177,0891.

1-Ethynylcyclohexyl acetate (**1m**)

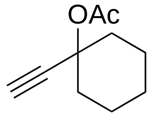

1m

Following part 2 of general procedure B starting from commercially available 1-ethynylcyclohexan-1-ol (5.00 mmol). Purification over SiO_2 (ethyl acetate/pentane 5%) gave product as a colorless oil, whose spectra match literature report.[7]

^{1}H NMR (400 MHz, $CDCl_3$) δ 2.59 (d, J = 2.4 Hz, 1 H), 2.16–2.08 (m, 2 H), 2.04 (d, J = 2.4 Hz, 3 H), 1.84 (dt, J = 14.2, 7.5 Hz, 2 H), 1.66–1.58 (m, 4 H), 1.55–1.48 (m, 1 H), 1.37–1.27 (m, 1 H); ^{13}C NMR (101 MHz, $CDCl_3$) δ 169.3, 83.7, 75.1, 74.2, 36.9, 25.1, 22.4, 21.9; HRMS (ESI): m/z calcd. for $C_{10}H_{14}NaO_2$ $[M+Na]^+$: 189.0886; found: 189.0888.

4-Phenylbut-3-yn-2-yl acetate (**1n**)

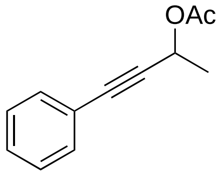

1n

Following part 2 of general procedure B starting from commercially available 4-phenyl-3-butyn-2-ol (5.00 mmol). Purification over SiO_2 (ethyl acetate/pentane 5%) gave product as a colorless oil, whose spectra match literature report.[5]

^{1}H NMR (400 MHz, $CDCl_3$) δ 7.45–7.27 (m, 5 H), 5.68 (q, J = 6.7 Hz, 1 H), 2.11 (s, 3 H), 1.58 (d, J = 6.6 Hz, 3 H); ^{13}C NMR (101 MHz, $CDCl_3$) δ 170.1, 132.0, 128.7, 128.4, 122.4, 87.5, 84.7, 61.0, 21.7, 21.3; HRMS (ESI): m/z calcd. for $C_{12}H_{12}NaO_2$ $[M+Na]^+$: 211.0730; found: 211.0734.

1-Phenylpent-1-yn-3-yl acetate (**1o**)

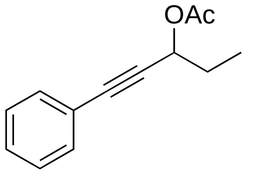

1o

Following part 2 of general procedure B starting from commercially available 1-phenylpent-1-yn-3-ol (5.00 mmol). Purification over SiO_2 (ethyl acetate/pentane 5%) gave product as a colorless oil, whose spectra match literature report.[3]

^{1}H NMR (400 MHz, $CDCl_3$) δ 7.45–7.43 (m, 2 H), 7.33–7.28 (m, 3 H), 5.56 (t, J = 6.5 Hz, 1 H), 2.11 (s, 3 H), 1.91–1.84 (m, 2 H), 1.07 (t, J = 7.4 Hz, 3 H); ^{13}C NMR (101 MHz, $CDCl_3$) δ 170.2, 132.0, 128.7, 128.4, 122.5, 86.5, 85.4, 65.8, 28.3, 21.2, 9.6; HRMS (ESI): m/z calcd. for $C_{13}H_{14}NaO_2$ $[M+Na]^+$: 225.0886; found: 225.0886.

2-Phenylbut-3-yn-2-yl acetate (**1p**)

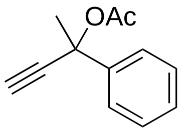

1p

Following part 2 of general procedure B starting from commercially available 1-phenyl-2-propyn-1-ol (5.00 mmol). Purification over SiO_2 (ethyl acetate/pentane 5%) gave product as a colorless oil, whose spectra match literature report.[8]

^{1}H NMR (400 MHz, $CDCl_3$) δ 7.59–7.57 (m, 2 H), 7.38–7.28 (m, 3 H), 2.81 (s, 1 H), 2.08 (s, 3 H), 1.90 (s, 3 H); ^{13}C NMR (101 MHz, $CDCl_3$) δ 168.7, 142.2, 128.5, 128.0, 124.8, 83.0, 75.7, 75.4, 32.1, 21.8; HRMS (ESI): m/z calcd. for $C_{12}H_{12}NaO_2$ $[M+Na]^+$: 211.0730; found: 211.0732.

1-((tert-Butyldimethylsilyl)oxy)-2-methylbut-3-yn-2-yl acetate (**1q**)

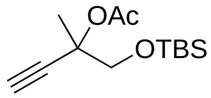

1q

Following part 2 of general procedure B starting from 1-((*tert*-butyldimethylsilyl)oxy)-2-methylbut-3-yn-2-ol (5.00 mmol). Purification over SiO_2 (ethyl acetate/pentane 5%) gave product as a colorless oil, whose spectra match literature report.[6]

^{1}H NMR (400 MHz, $CDCl_3$) δ 3.97 (d, *J* = 10.3 Hz, 1 H), 3.70 (d, *J* = 10.3 Hz, 1 H), 2.53 (s, 1 H), 2.03 (s, 3 H), 1.65 (s, 3 H), 0.90 (s, 9 H), 0.07 (d, *J* = 2.9 Hz, 6 H); ^{13}C NMR (101 MHz, $CDCl_3$) δ 169.5, 82.5, 74.8, 74.0, 68.4, 25.9, 23.4, 21.9, 18.4, -5.2 (x2); HRMS (ESI): m/z calcd. for $C_{13}H_{24}NaO_3Si$ $[M+Na]^+$: 279.1387; found: 279.1390.

Oct-1-yn-3-yl acetate (**1r**)

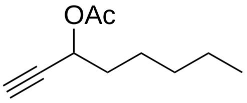

1r

Following part 2 of general procedure B starting from commercially available oct-1-yn-3-ol (5.00 mmol). Purification over SiO_2 (ethyl acetate/pentane 5%) gave product as a colorless oil, whose spectra match literature report.[8]

^{1}H NMR (400 MHz, $CDCl_3$) δ 5.33 (td, *J* = 6.7, 2.1 Hz, 1 H), 2.44 (d, *J* = 2.1 Hz, 1 H), 2.08 (s, 3 H), 1.76 (m, 2 H), 1.44 (m, 2 H), 1.31 (m, 4 H), 0.93–0.84 (m, 3 H); ^{13}C NMR (101 MHz, $CDCl_3$) δ 169.9, 81.4, 73.5, 63.9, 34.6, 31.3, 24.6, 22.5, 21.0, 14.0; HRMS (ESI): m/z calcd. for $C_{10}H_{16}NaO_2$ $[M+Na]^+$: 191.1043; found: 191.1044.

(3S,8R,10R,13S,14S)-17-(3-((tert-Butyldimethylsilyl)oxy)prop-1-yn-1-yl)-10,13-dimethyl-2,3,4,7,8,9,10,11,12,13,14,15,16,17-tetradecahydro-1H-cyclopenta[a]phenanthrene-3,17-diyl diacetate (**1s**)

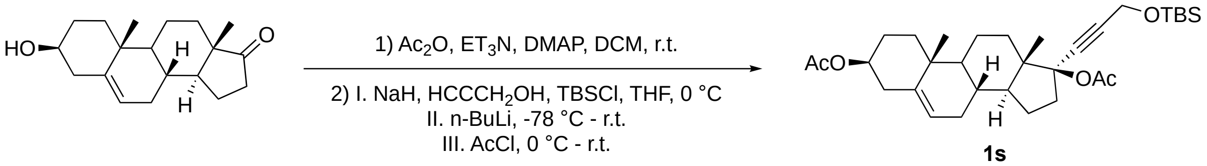

To a 100 mL round bottom flask containing dehydroepiandrosterone (1.00 g, 3.47 mmol, 1.00 equiv.), Et_3N (0.5 ml, 6.90 mmol, 2.00 equiv.), and DMAP (21 mg, 174 µmol, 5 mol%), in DCM (30 ml) was added Ac_2O (410 µl, 4.33 mmol, 1.25 equiv.). The solution was left stirring for 1 h before quenching with H_2O (10 ml), extracted with pentane (50 ml) and washed with 10% citric acid (10 ml), sat. $NaHCO_3$ (10 ml) and brine (10 ml). The combined organic phases were dried over $MgSO_4$ and concentrated under vacuum to give 1.12 g of pure product (98% yield). Propargyl alcohol (140 mg, 2.50 mmol, 1.10 equiv.) was added to a suspension of NaH (110 mg, 2.72 mmol, 60% in mineral oil, 1.20 equiv.) in THF (25 ml) at 0 °C. The mixture was stirred for 15 min, then TBSCl (377 mg, 2.50 mmol, 1.10 equiv.) was added. After 1.5 h stirring at room temperature, the mixture was cooled to –78°C and n-BuLi (0.96 mL, 2.39 mmol, 2.5

M in hexane, 1.05 equiv.) was added dropwise. Then, the reaction was left stirring for 1 h before adding acetyl-dehydroandrosterone (0.75 g, 2.27 mmol, 1.00 equiv.). After 2 h the reaction mixture was allowed to warm to 0 °C and AcCl (242 µl, 3.41 mmol, 1.5 equiv.) was added and the reaction was stirred overnight. The reaction was quenched with sat. $NaHCO_3$ (10 ml), extracted with pentane (25 ml), and washed with H_2O (10 ml) and brine (10 ml) and dried over $MgSO_4$. The evaporated crude mixture was purified by column chromatography on silica gel (SiO_2; diethyl ether/pentane 3-5%) to yield the product in 51% yield, whose spectra match those in a literature report.[9]

^{1}H NMR (400 MHz, $CDCl_3$) δ 5.35 (d, J = 5.1 Hz, 1 H), 4.59 (tdd, J = 10.6, 6.4, 4.2 Hz, 1 H), 4.34 (s, 2 H), 2.67 (ddd, J = 15.0, 9.7, 5.8 Hz, 1 H), 2.36–2.25 (m, 2H), 2.03–1.94 (m, 8 H), 1.84 (ddd, J = 11.4, 6.7, 3.6 Hz, 3 H), 1.72–1.18 (m, 9 H), 1.17–0.78 (m, 17 H), 0.09 (d, J = 1.7 Hz, 6 H). ^{13}C NMR (101 MHz, $CDCl_3$) δ 170.6, 169.5, 139.7, 122.3, 85.4, 84.8, 84.4, 73.9, 52.0, 49.7, 49.0, 47.7, 38.2, 37.2, 37.1, 36.7, 33.0, 32.4, 31.6, 27.8, 25.9, 23.8, 21.5 (x2), 20.7, 19.4, 18.3, 13.5, -5.0 (x2); HRMS (ESI): m/z calcd. for $C_{32}H_{50}NaO_5Si$ $[M+Na]^+$: 565.3320; found: 565.3328.

2.3 Synthesis of multisubstituted allenylboronates

General procedure

Catalyst $Fe(acac)_3$ (1.0 mol%) and B_2pin_2 (2.00 equiv.) were dissolved in a solution of TMEDA (1.0 mol%) in anhydrous THF (1.5 mL) in a dry 5 mL microwave flask and under argon. The resulting orange solution was cooled to 0 °C and the corresponding Grignard reagent (1.25 equiv.) was added, which led to a change in the color of the mixture from orange to dark brown. Then, the propargyl ester (0.30 mmol, 1.00 equiv.) was added dropwise. The reaction mixture was allowed to reach room temperature and was left for stirring for 16 hours. After that time, the crude mixture was purified by flash column chromatography over SiO_2 (eluent: pentane/diethyl ether) affording the desired product **2**.

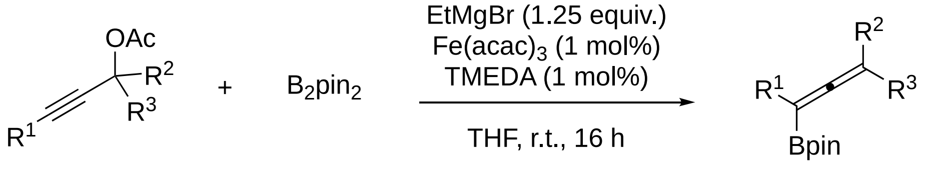

1a-1s (0.3 mmol) (2.0 equiv.) **2a-2s**

4,4,5,5-Tetramethyl-2-(5-methyl-1-phenylhexa-3,4-dien-3-yl)-1,3,2-dioxaborolane (**2a**)

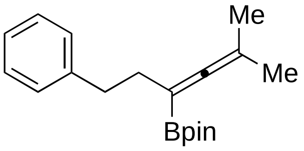

2a

Following general procedure: $Fe(acac)_3$ (1 mg, 0.003 mmol, 1.0 mol%), substrate **1a** (69 mg, 0.30 mmol, 1.00 equiv.), B_2pin_2 (152 mg, 0.60 mmol, 2.00 equiv.) and ethylmagnesium bromide (3.0 M in diethyl ether, 125 μL, 0.38 mmol, 1.25 equiv.) in anhydrous THF (1.5 mL) were allowed to react. The reaction mixture was purified over SiO_2 (pentane/diethyl ether 98:2) to yield product **2a** (75 mg, 88%) as a colorless oil, whose spectra match those in a literature report.[10]

^{1}H NMR (400 MHz, $CDCl_3$) δ 7.29–7.11 (m, 5 H), 2.74–2.68 (m, 2 H), 2.36–2.30 (m, 2 H), 1.62 (s, 6 H), 1.25 (s, 12 H); ^{13}C NMR (101 MHz, $CDCl_3$) δ 211.0, 142.8, 128.8, 128.2, 125.6, 91.7, 83.3, 35.8, 32.3, 24.9, 20.0. Carbons directly attached to boron atoms were not detected, most likely due to quadrupolar relaxation. HRMS (ESI): m/*z* calcd. for $C_{19}H_{27}BNaO_2$ $[M+Na]^+$: 321.200; found: 321.1968.

4,4,5,5-Tetramethyl-2-(3-methyl-1-phenylbuta-1,2-dien-1-yl)-1,3,2-dioxaborolane (**2b**)

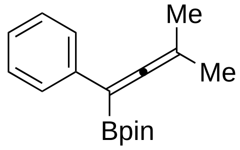

2b

Following general procedure: $Fe(acac)_3$ (1 mg, 0.003 mmol, 1 mol%), substrate **1b** (61 mg, 0.3 mmol, 1.0 equiv.), B_2pin_2 (152 mg, 0.60 mmol, 2.00 equiv.) and ethylmagnesium bromide (3.0 M in diethyl ether, 125 μL, 0.38 mmol, 1.25 equiv.) in anhydrous THF (1.5 mL) were allowed to react. The reaction mixture was purified over SiO_2 (pentane/diethyl ether 98:2) to yield product **2b** (69 mg, 85%) as a white solid, whose spectra match literature report.[11]

^{1}H NMR (400 MHz, $CDCl_3$) δ 7.55 (d, J = 6.8 Hz, 2 H), 7.32 (t, J = 7.7 Hz, 3 H), 7.19 (t, J = 7.4 Hz, 1 H), 1.87 (s, 6 H), 1.36 (s, 12 H); ^{13}C NMR (101 MHz, $CDCl_3$) δ 213.1, 137.8, 128.3, 128.0, 126.0, 93.8, 83.524.8, 19.6. Carbons directly attached to boron atoms were not detected, most likely due to quadrupolar relaxation. HRMS (ESI): m/*z* calcd. for $C_{17}H_{23}BNaO_2$ $[M+Na]^+$: 293.1683; found: 293.1685.

4,4,5,5-Tetramethyl-2-(2-methylocta-2,3-dien-4-yl)-1,3,2-dioxaborolane (**2c**)

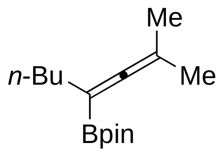

2c

Following general procedure: Fe(acac)$_3$ (1.0 mg, 0.003 mmol, 1.0 mol%), substrate **1c** (55 mg, 0.30 mmol, 1.00 equiv.), B$_2$pin$_2$ (152. mg, 0.60 mmol, 2.00 equiv.) and ethylmagnesium bromide (3.0 M in diethyl ether, 125 µL, 0.38 mmol, 1.25 equiv.) in anhydrous THF (1.5 mL) were allowed to react. The reaction mixture was purified over SiO$_2$ (pentane/diethyl ether 98:2) to yield product **2c** (55 mg, 74%) as a colorless oil, whose spectra match literature report.[10]
^{1}H NMR (400 MHz, CDCl$_3$) δ 2.01–1.97 (m, 2 H), 1.69 (s, 6 H), 1.41–1.30 (m, 4 H), 1.25 (s, 12 H), 0.88 (t, *J* = 7.1 Hz, 6 H); ^{13}C NMR (101 MHz, CDCl$_3$) δ 210.8, 91.0, 83.3, 31.7, 30.2, 24.9, 22.3, 20.1, 14.2. Carbons directly attached to boron atoms were not detected, most likely due to quadrupolar relaxation. HRMS (ESI): m/z calcd. for $C_{15}H_{27}BNaO_2$ [M+Na]$^+$: 273.1996; found: 273.1999.

2-(1-Cyclopentylidenehex-1-en-2-yl)-4,4,5,5-tetramethyl-1,3,2-dioxaborolane (**2d**)

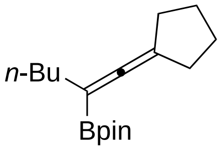

2d

Following general procedure: Fe(acac)$_3$ (1 mg, 0.003 mmol, 1 mol%), substrate **1d** (63 mg, 0.3 mmol, 1.0 equiv.), B$_2$pin$_2$ (152 mg, 0.6 mmol, 2 equiv.) and ethylmagnesium bromide (3.0 M in diethyl ether, 125 µL, 0.38 mmol, 1.25 equiv.) in anhydrous THF (1.5 mL) were allowed to react. The reaction mixture was purified over SiO$_2$ (pentane/diethyl ether 98:2) to yield product **2d** (63 mg, 76%) as a colorless oil, whose spectra match those in a literature report.[10]
^{1}H NMR (400 MHz, CDCl$_3$) δ 2.48–2.32 (m, 4 H), 2.01 (t, *J* = 7.0 Hz, 2 H), 1.72–1.58 (m, 4 H), 1.40–1.29 (m, 4 H), 1.24 (s, 12 H), 0.88 (t, *J* = 6.9 Hz, 3 H); ^{13}C NMR (101 MHz, CDCl$_3$) δ 206.4, 99.5, 83.2, 31.6, 30.9, 30.3, 27.4, 24.9, 22.3, 14.2. Carbons directly attached to boron atoms were not detected, most likely due to quadrupolar relaxation. HRMS (ESI): m/*z* calcd. for $C_{17}H_{29}BNaO_2$ [M+Na]$^+$: 299.2153; found: 299.2156.

2-(1-Cyclopentylidenehept-1-en-2-yl)-4,4,5,5-tetramethyl-1,3,2-dioxaborolane (**2e**)

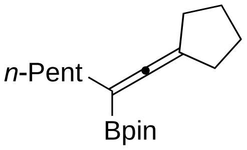

2e

Following general procedure: Fe(acac)$_3$ (1 mg, 0.003 mmol, 1 mol%), substrate **1e** (67 mg, 0.30 mmol, 1.00 equiv.), B$_2$pin$_2$ (152 mg, 0.60 mmol, 2.00 equiv.) and ethylmagnesium bromide (3.0 M in diethyl ether, 125 µL, 0.38 mmol, 1.25 equiv.) in anhydrous THF (1.5 mL) were allowed to react. The reaction mixture was purified over SiO$_2$ (pentane/diethyl ether 98:2) to yield product **2e** (64 mg, 73%) as a colorless oil.

^{1}H NMR (400 MHz, CDCl$_3$) δ 2.55–2.25 (m, 4 H), 2.01 (t, *J* = 7.2 Hz, 2 H), 1.65 (td, *J* = 7.7, 6.8, 4.0 Hz, 4 H), 1.42–1.25 (m, 18 H), 0.87 (t, *J* = 6.8 Hz, 3 H); ^{13}C NMR (101 MHz, CDCl$_3$) δ 206.4, 99.5, 83.2, 31.5, 31.0, 30.6, 29.1, 27.4, 24.9, 24.9, 14.3. Carbons directly attached to boron atoms were not detected, most likely due to quadrupolar relaxation. HRMS (ESI): m/z calcd. for $C_{18}H_{31}BNaO_2$ [M+Na]$^+$: 313.2313; found: 313.2308.

2-(1-Cyclohexylidenehept-1-en-2-yl)-4,4,5,5-tetramethyl-1,3,2-dioxaborolane (**2f**)

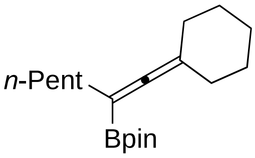

2f

Following general procedure: Fe(acac)$_3$ (1 mg, 0.003 mmol, 1 mol%), substrate **1f** (71 mg, 0.30 mmol, 1.00 equiv.), B$_2$pin$_2$ (152 mg, 0.6 mmol, 2 equiv.) and ethylmagnesium bromide (3.0 M in diethyl ether, 125 µL, 0.38 mmol, 1.25 equiv.) in anhydrous THF (1.5 mL) were allowed to react. The reaction mixture was purified over SiO$_2$ (pentane/diethyl ether 98:2) to yield product **2f** (56 mg, 61%) as a colorless oil.

^{1}H NMR (400 MHz, CDCl$_3$) δ 2.19–2.05 (m, 4 H), 1.99 (t, *J* = 7.3 Hz, 2 H), 1.71–1.35 (m, 8 H), 1.32–1.21 (m, 16 H), 0.88 (t, *J* = 6.8 Hz, 3 H); ^{13}C NMR (101 MHz, CDCl$_3$) δ 207.5, 98.7, 83.2, 31.4, 31.2, 30.3, 29.0, 27.8, 26.5, 25.0, 24.9, 22.8, 14.3. Carbons directly attached to boron atoms were not detected, most likely due to quadrupolar relaxation. HRMS (ESI): m/*z* calcd. for $C_{19}H_{33}BNaO_2$ [M+Na]$^+$: 327.2466; found: 327.2466.

tert-Butyldimethyl((4-methyl-6-phenyl-2-(4,4,5,5-tetramethyl-1,3,2-dioxaborolan-2-yl)hexa-2,3-dien-1-yl)oxy)silane (**2g**)

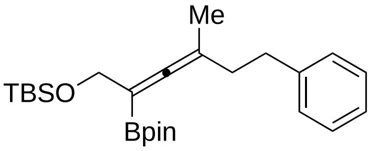

2g

Following general procedure: Fe(acac)$_3$ (1 mg, 0.003 mmol, 1 mol%), substrate **1g** (108 mg, 0.30 mmol, 1.00 equiv.), B$_2$pin$_2$ (152 mg, 0.60 mmol, 2.00 equiv.) and ethylmagnesium bromide (3.0 M in diethyl ether, 125 µL, 0.38 mmol, 1.25 equiv.) in anhydrous THF (1.5 mL) were allowed to react. The reaction mixture was purified over SiO$_2$ (pentane/diethyl ether 98:2) to yield product **2g** (107 mg, 83%) as a white solid, whose spectra match those in a literature report.[12]

^{1}H NMR (400 MHz, CDCl$_3$) δ 7.32–7.09 (m, 5 H), 4.23–4.07 (m, 2 H), 2.72 (td, *J* = 7.3, 3.1 Hz, 2 H), 2.26 (td, *J* = 7.3, 2.5 Hz, 2 H), 1.74 (s, 3 H), 1.26 (d, *J* = 2.9 Hz, 12 H), 0.90 (s, 9 H), 0.06 (s, 6 H); ^{13}C NMR (101 MHz, CDCl$_3$) δ 210.0, 142.6, 128.5, 128.4, 125.8, 97.3, 83.4, 62.6, 35.4, 34.4, 26.0, 25.0, 24.7, 18.6, 18.4, -5.0. Carbons directly attached to boron atoms were not detected, most likely due to quadrupolar relaxation. HRMS (ESI): m/z calcd. for $C_{25}H_{41}BNaO_3Si$ [M+Na]$^+$: 451.2815; found: 451.2815.

tert-Butyl((3-cyclohexylidene-2-(4,4,5,5-tetramethyl-1,3,2-dioxaborolan-2-yl)allyl)oxy)dimethylsilane (**2h**)

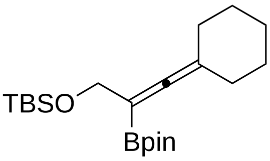

2h

Following general procedure: Fe(acac)$_3$ (1 mg, 0.003 mmol, 1 mol%), substrate **1h** (93 mg, 0.30 mmol, 1.00 equiv.), B$_2$pin$_2$ (152 mg, 0.60 mmol, 2.00 equiv.) and ethylmagnesium bromide (3.0 M in diethyl ether, 125 µL, 0.38 mmol, 1.25 equiv.) in anhydrous THF (1.5 mL) were allowed to react. The reaction mixture was purified over SiO$_2$ (pentane/diethyl ether 98:2) to yield product **2h** (61 mg, 54%) as a white solid, whose spectra match those in a literature report.[13]

^{1}H NMR (400 MHz, CDCl$_3$) δ 4.19 (s, 2 H), 2.14 (t, *J* = 5.9 Hz, 4 H), 1.67–1.60 (m, 2 H), 1.57–1.43 (m, 4 H), 1.24 (s, 12 H), 0.89 (s, 9 H), 0.05 (s, 6 H); ^{13}C NMR (101 MHz, CDCl$_3$) δ 207.1, 100.0, 83.3, 63.2, 30.8, 27.5, 26.4, 26.1, 24.9, 18.5, -5.0. Carbons directly attached to

boron atoms were not detected, most likely due to quadrupolar relaxation. HRMS (ESI): m/z calcd. for $C_{21}H_{39}BNaO_3Si$ $[M+Na]^+$: 401.2654; found: 401.2650.

tert-Butyldimethyl((5-methyl-7-phenyl-3-(4,4,5,5-tetramethyl-1,3,2-dioxaborolan-2-yl)hepta-3,4-dien-1-yl)oxy)silane (**2i**)

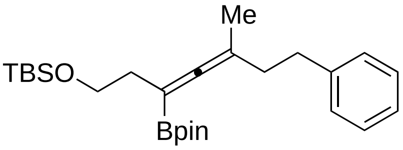

2i

Following general procedure: $Fe(acac)_3$ (1 mg, 0.003 mmol, 1 mol%), substrate **1i** (112 mg, 0.30 mmol, 1.00 equiv.), B_2pin_2 (152 mg, 0.60 mmol, 2.00 equiv.) and ethylmagnesium bromide (3.0 M in diethyl ether, 125 µL, 0.38 mmol, 1.25 equiv.) in anhydrous THF (1.5 mL) were allowed to react. The reaction mixture was purified over SiO_2 (pentane/diethyl ether 98:2) to yield product **2i** (108 mg, 81%) as a white solid.

^1H NMR (400 MHz, $CDCl_3$) δ 7.32–7.12 (m, 5 H), 3.63 (t, J = 7.6 Hz, 2 H), 2.74–2.66 (m, 2 H), 2.30–2.18 (m, 4 H), 1.71 (s, 3 H), 1.25 (d, J = 3.0 Hz, 12 H), 0.90 (s, 9 H), 0.06 (s, 6 H); ^{13}C NMR (101 MHz, $CDCl_3$) δ 211.3, 142.6, 128.5, 128.4, 125.8, 95.2, 83.4, 63.8, 35.3, 34.4, 34.4, 26.2, 25.0, 24.7, 18.8, 18.6, -5.0. Carbons directly attached to boron atoms were not detected, most likely due to quadrupolar relaxation. HRMS (ESI): m/z calcd. for $C_{26}H_{43}BNaO_3Si$ $[M+Na]^+$: 465.2967; found: 465.2967.

tert-Butyl-2,2-bis(((tert-butyldimethylsilyl)oxy)methyl)-3-methyl-5-phenyl-5-(4,4,5,5-tetramethyl-1,3,2-dioxaborolan-2-yl)penta-3,4-dienoate (**2j**)

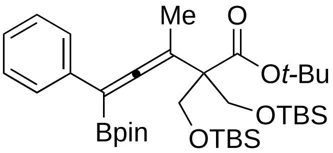

2j

Following general procedure: $Fe(acac)_3$ (1 mg, 0.003 mmol, 1 mol%), substrate **1j** (177 mg, 0.30 mmol, 1.00 equiv.), B_2pin_2 (152 mg, 0.60 mmol, 2.00 equiv.) and ethylmagnesium bromide (3.0 M in diethyl ether, 125 µL, 0.38 mmol, 1.25 equiv.) in anhydrous THF (1.5 mL) were allowed to react. The reaction mixture was purified over SiO_2 (pentane/diethyl ether 98:2) to yield product **2j** (165 mg, 84%) as a white solid.

^1H NMR (400 MHz, $CDCl_3$) δ 7.62 (d, J = 7.1 Hz, 2 H), 7.28 (t, J = 7.5 Hz, 2 H), 7.16 (t, J = 7.3 Hz, 1 H), 3.99 (dd, J = 9.5, 4.9 Hz, 2 H), 3.86 (dd, J = 21.1, 9.5 Hz, 2 H), 1.79 (s, 3 H),

1.45 (s, 9 H), 1.32 (s, 12 H), 0.93 (d, *J* = 2.2 Hz, 18 H), 0.09 (s, 3 H), 0.06 (s, 9 H); ^{13}C NMR (101 MHz, $CDCl_3$) δ 213.1, 171.8, 137.1, 128.3, 128.2, 126.3, 96.8, 83.6, 80.6, 61.0, 60.7, 56.4, 28.2, 26.2, 26.1, 25.0, 24.7, 18.6, 18.4, 14.7, -5.2, -5.4, -5.4. Carbons directly attached to boron atoms were not detected, most likely due to quadrupolar relaxation. HRMS (ESI): m/z calcd. for $C_{36}H_{63}BNaO_6Si_2$ $[M+Na]^+$: 681.4148; found: 681.4152.

*4,4,5,5-Tetramethyl-2-(3-methylbuta-1,2-dien-1-yl)-1,3,2-*dioxaborolane (**2k**)

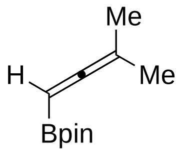

2k

Following general procedure: $Fe(acac)_3$ (1 mg, 0.003 mmol, 1 mol%), substrate **1k** (38 mg, 0.30 mmol, 1.00 equiv.), B_2pin_2 (152 mg, 0.60 mmol, 2.00 equiv.) and ethylmagnesium bromide (3.0 M in diethyl ether, 125 µL, 0.38 mmol, 1.25 equiv.) in anhydrous THF (1.5 mL) were allowed to react. The reaction mixture was purified over SiO_2 (pentane/diethyl ether 98:2) to yield product **2l** (45 mg, 77%) as a colorless oil.

^{1}H NMR (400 MHz, $CDCl_3$) δ 4.75 (p, *J* = 3.6 Hz, 1 H), 1.67 (d, *J* = 3.7 Hz, 6 H), 1.24 (d, *J* = 1.3 Hz, 12 H); ^{13}C NMR (101 MHz, $CDCl_3$) δ 215.5, 89.9, 83.4, 24.8, 19.5. Carbons directly attached to boron atoms were not detected, most likely due to quadrupolar relaxation. HRMS (ESI): m/z calcd. for $C_{11}H_{19}BNaO$ $[M+Na]^+$: 217.1370; found: 217.1372.

2-(3-Ethylpenta-1,2-dien-1-yl)-4,4,5,5-tetramethyl-1,3,2-dioxaborolane (**2l**)

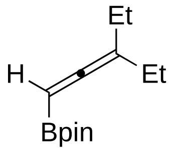

2l

Following general procedure: $Fe(acac)_3$ (1 mg, 0.003 mmol, 1 mol%), substrate **1l** (46 mg, 0.30 mmol, 1.00 equiv.), B_2pin_2 (152 mg, 0.60 mmol, 2.00 equiv.) and ethylmagnesium bromide (3.0 M in diethyl ether, 125 µL, 0.38 mmol, 1.25 equiv.) in anhydrous THF (1.5 mL) were allowed to react. The reaction mixture was purified over SiO_2 (pentane/diethyl ether 98:2) to yield product **2m** (61 mg, 92%) as a colorless oil.

^{1}H NMR (400 MHz, $CDCl_3$) δ 4.94 (t, *J* = 4.0 Hz, 1 H), 2.01–1.89 (m, 4 H), 1.25 (s, 12 H), 1.05–0.95 (m, 6 H); ^{13}C NMR (101 MHz, $CDCl_3$) δ 214.6, 97.8, 83.3, 24.8, 24.7, 12.4. Carbons

directly attached to boron atoms were not detected, most likely due to quadrupolar relaxation. HRMS (ESI): m/z calcd. for $C_{13}H_{23}BNaO_2$ $[M+Na]^+$: 245.1686; found: 245.1645.

2-(2-Cyclohexylidenevinyl)-4,4,5,5-tetramethyl-1,3,2-dioxaborolane (**2m**)

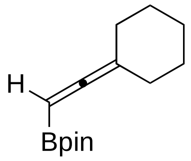

2m

Following general procedure: $Fe(acac)_3$ (1 mg, 0.003 mmol, 1 mol%), substrate **1m** (46 mg, 0.30 mmol, 1.00 equiv.), B_2pin_2 (152 mg, 0.60 mmol, 2.00 equiv.) and ethylmagnesium bromide (3.0 M in diethyl ether, 125 µL, 0.38 mmol, 1.25 equiv.) in anhydrous THF (1.5 mL) were allowed to react. The reaction mixture was purified over SiO_2 (pentane/diethyl ether 98:2) to yield product **2n** (51 mg, 77%) as a colorless oil, whose spectra match those in a literature report.[8]

^{1}H NMR (400 MHz, $CDCl_3$) δ 4.77 (p, J = 2.5 Hz, 1 H), 2.14 (tt, J = 8.7, 5.4 Hz, 4 H), 1.65 (dd, J = 13.2, 4.5 Hz, 2 H), 1.61–1.38 (m, 2 H), 1.25 (s, 12 H); ^{13}C NMR (101 MHz, $CDCl_3$) δ 212.6, 97.0, 83.4, 30.5, 27.3, 26.3, 25.0. Carbons directly attached to boron atoms were not detected, most likely due to quadrupolar relaxation. HRMS (ESI): m/z calcd. for $C_{14}H_{23}BNaO_2$ $[M+Na]^+$: 257.1683; found: 257.1687.

4,4,5,5-Tetramethyl-2-(3-phenylbuta-1,2-dien-1-yl)-1,3,2-dioxaborolane (**2n**)

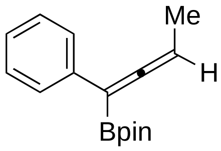

2n

Following general procedure: $Fe(acac)_3$ (1 mg, 0.003 mmol, 1 mol%), substrate **1n** (56 mg, 0.30 mmol, 1.00 equiv.), B_2pin_2 (152 mg, 0.60 mmol, 2.00 equiv.) and ethylmagnesium bromide (3.0 M in diethyl ether, 125 µL, 0.38 mmol, 1.25 equiv.) in anhydrous THF (1.5 mL) were allowed to react. The reaction mixture was purified over SiO_2 (ethyl pentane/diethyl ether 98:2) to yield product **2o** (55 mg, 72%) as a colorless oil, whose spectra match literature report.[14]

^{1}H NMR (400 MHz, $CDCl_3$) δ 7.39–7.14 (m, 5 H), 5.28 (q, J = 3.6 Hz, 1 H), 2.11 (d, J = 3.5 Hz, 3 H), 1.29 (d, J = 3.9 Hz, 12 H); ^{13}C NMR (101 MHz, $CDCl_3$) δ 218.1, 136.7, 128.5, 126.5, 125.9, 96.4, 83.9, 25.1, 24.9, 16.2. Carbons directly attached to boron atoms were not detected,

most likely due to quadrupolar relaxation. HRMS (ESI): m/z calcd. for $C_{16}H_{21}BNaO_2$ $[M+Na]^+$: 279.1527; found: 279.1530.

4,4,5,5-Tetramethyl-2-(1-phenylpenta-1,2-dien-1-yl)-1,3,2-dioxaborolane (**2o**)

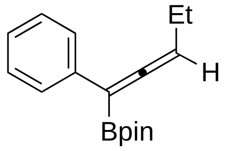

2o

Following general procedure: $Fe(acac)_3$ (1 mg, 0.003 mmol, 1 mol%), substrate **1o** (61 mg, 0.30 mmol, 1.00 equiv.), B_2pin_2 (152 mg, 0.60 mmol, 2.00 equiv.) and ethylmagnesium bromide (3.0 M in diethyl ether, 125 μL, 0.38 mmol, 1.25 equiv.) in anhydrous THF (1.5 mL) were allowed to react. The reaction mixture was purified over SiO_2 (pentane/diethyl ether 98:2) to yield product **2p** (70 mg, 87%) as a colorless oil, whose spectra match those in a literature report.[15]

^1H NMR (400 MHz, $CDCl_3$) δ 7.55 (d, J = 7.1 Hz, 2 H), 7.29 (t, J = 7.7 Hz, 2 H), 7.17 (t, J = 7.4 Hz, 1 H), 5.54 (t, J = 6.3 Hz, 1 H), 2.22–2.10 (m, 2 H), 1.33 (d, J = 2.3 Hz, 12 H), 1.09 (t, J = 7.4 Hz, 3 H); ^{13}C NMR (101 MHz, $CDCl_3$) δ 213.9, 136.8, 128.4, 127.9, 126.4, 91.7, 83.9, 25.1, 24.6, 21.2, 13.7. Carbons directly attached to boron atoms were not detected, most likely due to quadrupolar relaxation. HRMS (ESI): m/z calcd. for $C_{17}H_{23}BNaO_2$ $[M+Na]^+$: 293.1683; found: 293.1683.

4,4,5,5-Tetramethyl-2-(1-phenylbuta-1,2-dien-1-yl)-1,3,2-dioxaborolane (**2p**)

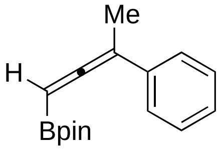

2p

Following general procedure: $Fe(acac)_3$ (1 mg, 0.003 mmol, 1 mol%), substrate **1p** (57 mg, 0.30 mmol, 1.00 equiv.), B_2pin_2 (152 mg, 0.60 mmol, 2.00 equiv.) and ethylmagnesium bromide (3.0 M in diethyl ether, 125 μL, 0.38 mmol, 1.25 equiv.) in anhydrous THF (1.5 mL) were allowed to react. The reaction mixture was purified over SiO_2 (pentane/diethyl ether 98:2) to yield product **2q** (67 mg, 86%) as a colorless oil.

^1H NMR (400 MHz, $CDCl_3$) δ 7.53 (d, J = 7.0 Hz, 2 H), 7.29 (t, J = 7.8 Hz, 2 H), 7.17 (t, J = 7.3 Hz, 1 H), 5.43 (q, J = 7.1 Hz, 1 H), 1.79 (d, J = 7.1 Hz, 3 H), 1.33 (s, 12 H); ^{13}C NMR (101 MHz, $CDCl_3$) δ 215.0, 136.8, 128.4, 128.1, 126.4, 84.5, 83.9, 25.0, 24.8, 13.2. Carbons directly

attached to boron atoms were not detected, most likely due to quadrupolar relaxation. HRMS (ESI): m/z calcd. for $C_{16}H_{21}BNaO_2$ $[M+Na]^+$: 279.1527; found: 279.1531.

tert-Butyldimethyl((2-methyl-4-(4,4,5,5-tetramethyl-1,3,2-dioxaborolan-2-yl)buta-2,3-dien-1-yl)oxy)silane (**2q**)

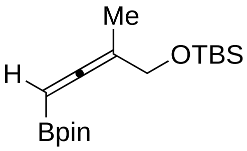

2q

Following general procedure: $Fe(acac)_3$ (1 mg, 0.003 mmol, 1 mol%), substrate **1q** (77 mg, 0.30 mmol, 1.00 equiv.), B_2pin_2 (152 mg, 0.60 mmol, 2.00 equiv.) and ethylmagnesium bromide (3.0 M in diethyl ether, 125 µL, 0.38 mmol, 1.25 equiv.) in anhydrous THF (1.5 mL) were allowed to react. The reaction mixture was purified over SiO_2 (pentane/diethyl ether 98:2) to yield product **2r** (73 mg, 75%) as a colorless oil.

^{1}H NMR (400 MHz, $CDCl_3$) δ 4.91 (q, J = 3.2 Hz, 1 H), 4.15 (d, J = 2.7 Hz, 2 H), 1.71 (d, J = 3.5 Hz, 3 H), 1.26 (d, J = 1.6 Hz, 12 H), 0.89 (s, 9 H), 0.07 (d, J = 5.4 Hz, 6 H); ^{13}C NMR (101 MHz, $CDCl_3$) δ 214.5, 94.9, 83.6, 65.1, 26.2, 25.0 (x2), 18.6, 14.8, -5.0 (x2). Carbons directly attached to boron atoms were not detected, most likely due to quadrupolar relaxation. HRMS (ESI): m/z calcd. for $C_{17}H_{33}BNaO_3Si$ $[M+Na]^+$: 347.2184; found: 347.2188.

4,4,5,5-Tetramethyl-2-(octa-1,2-dien-1-yl)-1,3,2-dioxaborolane (**2r**)

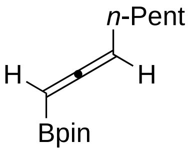

2r

Following general procedure: $Fe(acac)_3$ (1 mg, 0.003 mmol, 1 mol%), substrate **1r** (50 mg, 0.30 mmol, 1.00 equiv.), B_2pin_2 (152 mg, 0.60 mmol, 2.00 equiv.) and ethylmagnesium bromide (3.0 M in diethyl ether, 125 µL, 0.38 mmol, 1.25 equiv.) in anhydrous THF (1.5 mL) were allowed to react. The reaction mixture was purified over SiO_2 (pentane/diethyl ether 98:2) to yield product **2s** (56 mg, 79%) as a colorless oil, whose spectra match those in a literature report.[8]

^{1}H NMR (400 MHz, $CDCl_3$) δ 5.06 (q, J = 6.8 Hz, 1 H), 4.88 (dt, J = 7.0, 3.6 Hz, 1 H), 2.02 (qd, J = 7.0, 3.4 Hz, 2 H), 1.43–1.37 (m, 2 H), 1.35–1.32 (m, 4 H), 1.31–1.26 (m, 12 H), 0.88 (t, J = 7.0 Hz , 3 H); ^{13}C NMR (101 MHz, $CDCl_3$) δ 216.6, 86.1, 83.6, 31.3, 28.9, 27.4, 25.0,

24.7, 22.6, 14.2. Carbons directly attached to boron atoms were not detected, most likely due to quadrupolar relaxation. HRMS (ESI): m/z calcd. for $C_{14}H_{25}BNaO_2$ $[M+Na]^+$: 259.1840; found: 259.1843.

(3S,8S,10R,13S,14S)-17-(3-((tert-Butyldimethylsilyl)oxy)-2-(4,4,5,5-tetramethyl-1,3,2-dioxaborolan-2-yl)prop-1-en-1-ylidene)-10,13-dimethyl-2,3,4,7,8,9,10,11,12,13,14,15,16,17-tetradecahydro-1H-cyclopenta[a]phenanthren-3-yl acetate (**2s**)

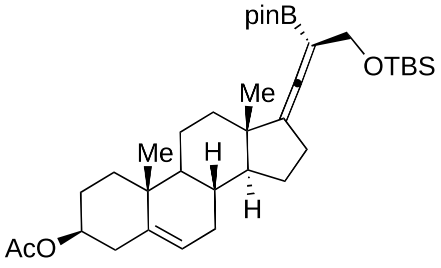

2s

Following general procedure: $Fe(acac)_3$ (1 mg, 0.003 mmol, 1 mol%), substrate **1s** (163 mg, 0.30 mmol, 1.00 equiv.), B_2pin_2 (152 mg, 0.60 mmol, 2.00 equiv.) and ethylmagnesium bromide (3.0 M in diethyl ether, 125 µL, 0.38 mmol, 1.25 equiv.) in anhydrous THF (1.5 mL) were allowed to react. The reaction mixture was purified over SiO_2 (pentane/diethyl ether 98:2) to yield product **2k** (145 mg, 79%) as a white solid (*dr* = 6.6:1), whose spectra match literature report.[9]

^{1}H NMR (400 MHz, $CDCl_3$) δ 5.38 (m, 1 H), 4.61 (tdd, J = 10.5, 6.3, 4.0 Hz, 1 H), 4.22 (d, J = 12.1 Hz, 1 H), 4.13 (d, J = 12.1 Hz, 1 H), 2.70–2.60 (m, 1 H), 2.43 (dt, J = 16.8, 8.7 Hz, 1 H), 2.32 (dd, J = 9.4, 3.8 Hz, 2 H), 2.01 (d, J = 16.8 Hz, 5 H), 1.87 (dt, J = 14.8, 4.0 Hz, 2 H), 1.63–1.40 (m, 6 H), 1.39–1.08 (m, 18 H), 1.03 (s, 3 H), 0.89 (d, J = 4.0 Hz, 12 H), 0.04 (d, J = 1.7 Hz, 5 H); ^{13}C NMR (101 MHz, $CDCl_3$) δ 205.2, 170.7, 140.0, 122.6, 110.3, 83.2, 74.1, 63.0, 56.0, 50.4, 45.3, 38.3, 37.2, 36.9, 36.4, 32.0 (x2), 29.9, 27.9 (x2), 27.0, 26.1, 25.3, 25.2, 24.5, 21.6, 21.2, 19.5, 19.4, 18.6, 18.5, 14.3, 14.2, -4.9, -5.0. Carbons directly attached to boron atoms were not detected, most likely due to quadrupolar relaxation. HRMS (ESI): m/*z* calcd. for $C_{36}H_{59}BNaO_5Si$ $[M+Na]^+$: 633.4117; found: 633.4122.

2.4 Study of the stereochemistry

Preparation of propargyl methyl ether (*S*)-1p:

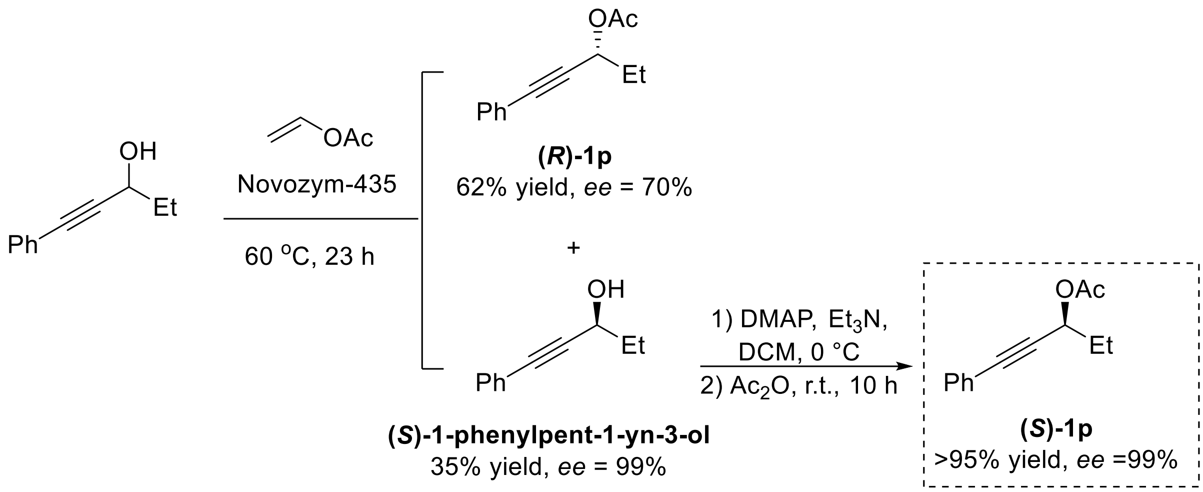

According to a reported procedure,[16] racemic 1-phenylpent-1-yn-3-ol (2.0 g, 12.5 mmol) was dissolved in 100 ml of vinyl acetate. Novozym-435 (300 mg) was added to the solution and it was left stirring at 60 °C for 23 hours. Then, the mixture was filtrated an extracted with diethyl ether (2 x 30 mL). The solvent was evaporated and the crude was purified over SiO_2 (ethyl acetate/pentane) to yield product **(*R*)-1p** (62% yield, 70% *ee*) and **(*S*)**-phenylpent-1-yn-3-ol (35% yield, 99% *ee*). In a second step, the **(*S*)**-alcohol is acetylated following part 2 general procedure B affording compound **(*S*)-1p** in quantitative yield and with complete retention of configuration. Optical rotation: $[\alpha]^{20}_{D}$ = -17.3 (c = 0.01 g/cm^3, $CHCl_3$). The absolute configuration was measured by SFC. Conditions: Chiral column ADH, 5% MeOH, flow rate = 1.0 mL/min, wavelength = 230 nm.

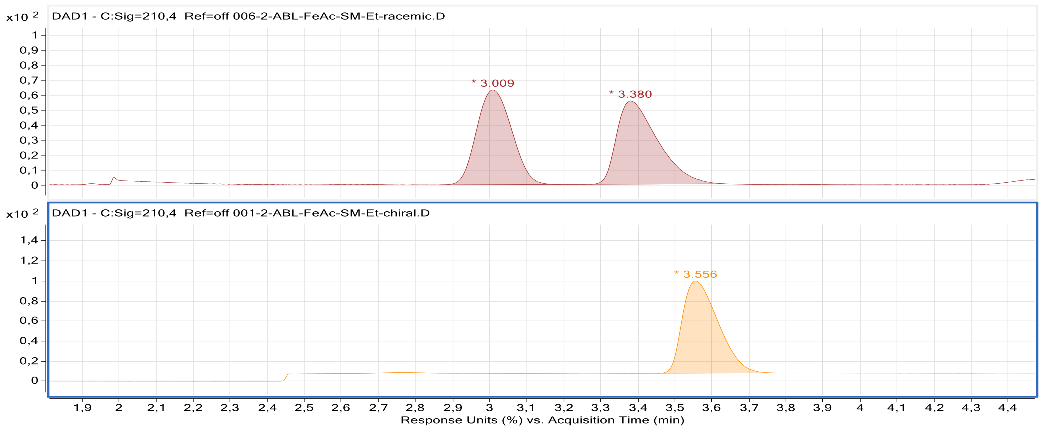

(S)-4,4,5,5-Tetramethyl-2-(1-phenylpenta-1,2-dien-1-yl)-1,3,2-dioxaborolane (**(*S*)-2p**)

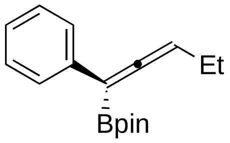

(*S*)-2p

Following general procedure: Fe(acac)$_3$ (1 mg, 0.003 mmol, 1.0 mol%), substrate **(*S*)-1p** (61 mg, 0.30 mmol, 1.00 equiv.), B_2pin_2 (152 mg, 0.6 mmol, 2 equiv.) and ethylmagnesium bromide (3.0 M in diethyl ether, 125 µL, 0.38 mmol, 1.25 equiv.) in anhydrous THF (1.5 mL) were allowed to react. The reaction mixture was purified over SiO_2 (pentane/diethyl ether 98:2) to yield product **(*S*)-2p** as a colorless oil, whose spectra match those in a literature report, with an enantiomeric excess of 87%.

Optical rotation: $[\alpha]^{20}_{D}$ = +20.1 (c = 0.01 g/cm^3, $CHCl_3$). The absolute configuration was measured by SFC. Conditions: Chiral column IC, 5% MeOH, flow rate = 1.0 mL/min, wavelength = 230 nm, and it is in accordance to that previously reported in the literature.[17]

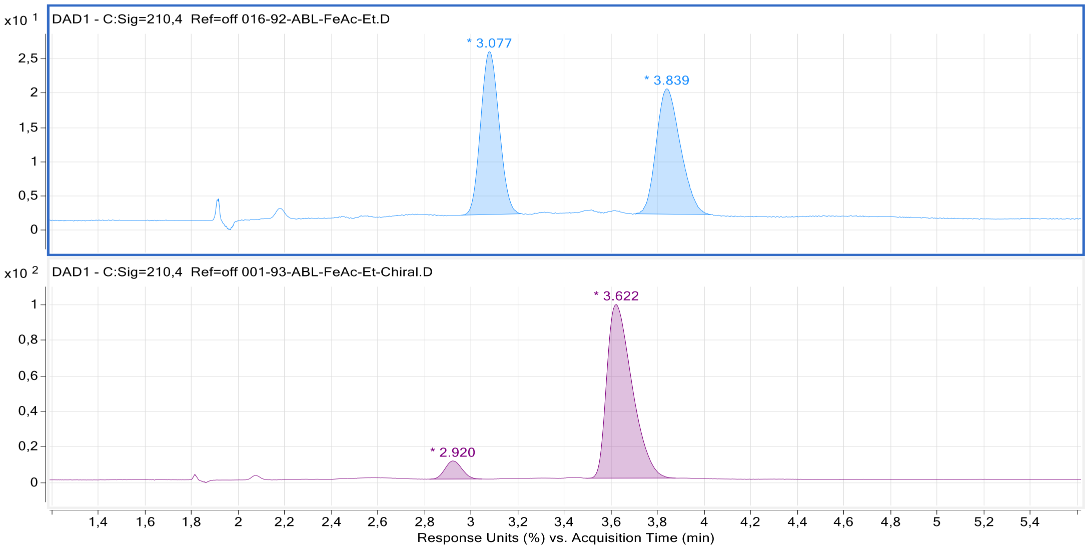

3. Scale-up synthesis of allenylboronates

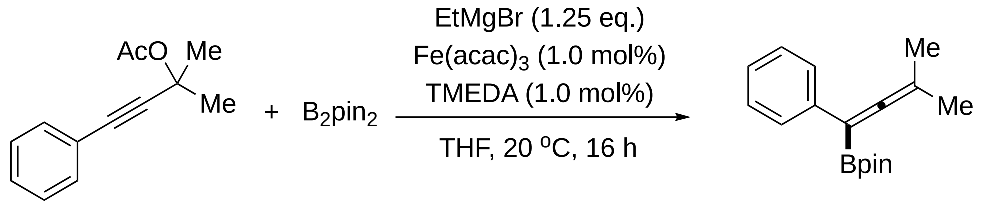

1b, 5.0 g

2b, 5.21 g, 77%

Following the general procedure: $Fe(acac)_3$ (90 mg, 0.25 mmol, 1.0 mol%), substrate **1b** (5.0 g, 25.00 mmol, 1.00 equiv.), B_2pin_2 (12.5 g, 50.00 mmol, 2.00 equiv.), TMEDA (1.2 mL, 0.14 mmol, 1.0 mol%) and ethylmagnesium bromide (3.0 M in diethyl ether, 10.3 mL, 31.0 mmol, 1.25 equiv.) in anhydrous THF (120 mL) were allowed to react. The reaction mixture was purified over SiO_2 (pentane/diethyl ether 98:2) to yield product **2b** (5.21 g, 77%) as a white solid.

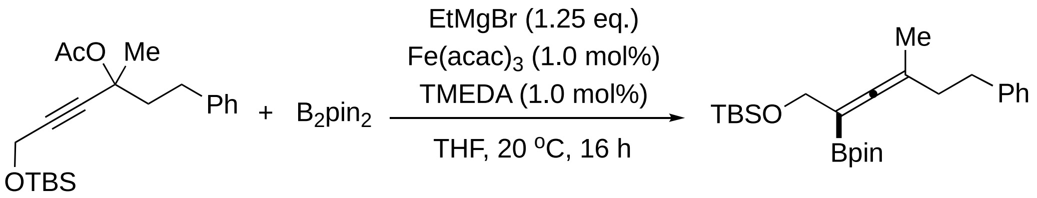

1c, 5.0 g

2c, 3.87 g, 65%

Following general procedure: $Fe(acac)_3$ (49.0 mg, 0.14 mmol, 1.0 mol%), substrate **1c** (5.0 g, 13.90 mmol, 1.0 equiv.), B_2pin_2 (7.0 g, 27.7 mmol, 2.00 equiv.), TMEDA (1.4 mL, 0.14 mmol, 1.0 mol%) and ethylmagnesium bromide (3.0 M in diethyl ether, 5.8 mL, 17.30 mmol, 1.25 equiv.) in anhydrous THF (120 mL) were reacted. The reaction mixture was purified over SiO_2 (pentane/diethyl ether 98:2) to yield product **2c** (3.87 g, 65%) as a colorless oil.

4. Selected applications of allenylboronates

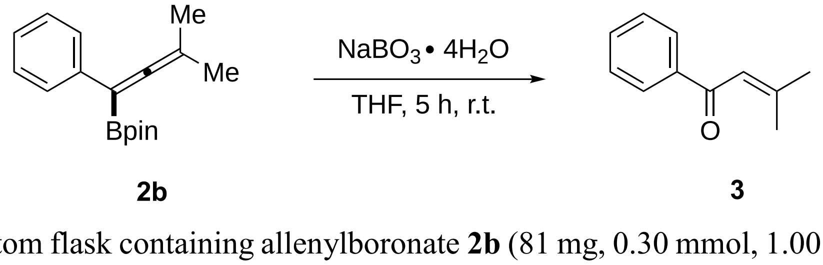

2b **3**

To a round bottom flask containing allenylboronate **2b** (81 mg, 0.30 mmol, 1.00 equiv.) in TFH (1 mL) and H_2O (1 mL), was added $NaBO_3 \cdot 4H_2O$ (235 mg, 1.53 mmol, 5.00 equiv.). The reaction mixture was left stirring at room temperature for 5 hours. Then, it was extracted with

ethyl acetate (3 x 10 mL), dried over $MgSO_4$ and concentrated under vacuum to yield product **3** as a colorless oil in 83% isolated yield.

^{1}H NMR (400 MHz, $CDCl_3$) δ 7.97–7.88 (m, 2 H), 7.52 (t, *J* = 7.3 Hz, 1 H), 7.44 (t, *J* = 7.5 Hz, 2 H), 6.79–6.71 (m, 1 H), 2.21 (s, 3 H), 2.02 (s, 3 H); ^{13}C NMR (101 MHz, $CDCl_3$) δ 191.7, 156.8, 139.4, 132.4, 131.8, 128.6, 128.4, 128.3, 121.3, 28.1, 21.3. HRMS (ESI): m/*z* calcd. for $C_{11}H_{12}NaO$ $[M+Na]^+$: 183.0780; found: 183.0790.

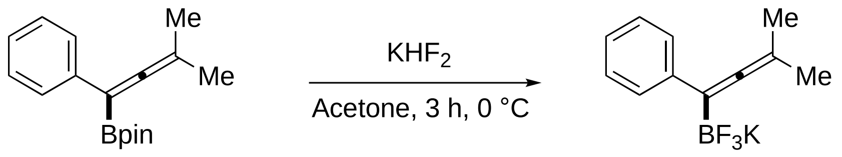

2b **4**

To a round bottom flask containing allenylboronate **2b** (81 mg, 0.30 mmol, 1.00 equiv.) in acetone (5 mL) at 0 °C, KHF_2 (119 mg, 1.53 mmol, 5.00 equiv.) and deionized water (5 mL) were added under inert atmosphere. After three hours stirring, the crude was filtered and the solvent removed under vacuum. The resulting crude mixture was extracted with hot acetone (3 x 10 mL) and filtered. The solution was dried under vacuum and then 10 mL of pentane were added. A white precipitate was formed and the mixture was left in the freezer for 6 hours. The crystals formed (**4**) were filtered off and washed with cold pentane (88% isolated yield).

^{1}H NMR (400 MHz, Acetone- *d*$_6$) δ 7.51 (d, *J* = 7.7 Hz, 2 H), 7.09 (t, *J* = 7.6 Hz, 2 H), 6.93 (t, *J* = 7.3 Hz, 1 H), 1.67 (s, 6 H); ^{13}C NMR (101 MHz, Acetone- *d*$_6$) δ 143.4, 133.1, 132.2, 129.4, 129.3, 129.3, 128.8, 128.0, 124.7, 88.0, 20.7. ^{19}F NMR (376 MHz, Acetone- *d*$_6$) δ –137.1.

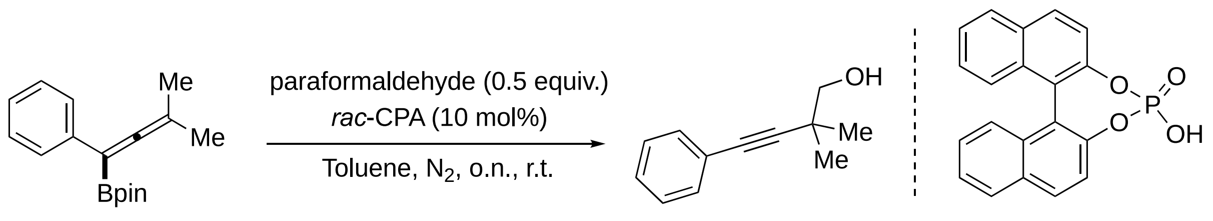

2b **5** *rac*-CPA

To a round bottom flask containing allenylboronate **2b** (81 mg, 0.30 mmol, 1.00 equiv.) in toluene 2 mL) under inert atmosphere, were added paraformaldehyde (0.15 mmol, 7 mg, 0.50 equiv.) and racemic phosphoric acid (0.03 mmol, 11 mg, 0.10 equiv.). The mixture was left stirring overnight at room temperature, before addition of $NaHCO_3$ (5 mL). The resulting biphasic mixture was stirred for 1 more hour and extracted with ethyl acetate (2x50 mL). The combined organic phases were dried over $MgSO_4$, filtered and concentrated under vacuum.

Purification by column chromatography (pentane/diethyl ether 90:10) yielded product **5** in 86%, whose spectra match those in a literature report.[18]

^{1}H NMR (400 MHz, $CDCl_3$) δ 7.43–7.37 (m, 2 H), 7.32–7.27 (m, 3 H), 3.49 (s, 2 H), 1.31 (s, 6 H); ^{13}C NMR (101 MHz, $CDCl_3$) δ 131.82, 128.35, 128.03, 123.40, 94.40, 82.24, 71.84, 35.00, 25.63. HRMS (ESI): m/*z* calcd. for $C_{12}H_{14}NaO$ $[M+Na]^+$: 197.0937; found: 197.0942.

5. NMR Spectra

2-Methyl-6-phenylhex-3-yn-2-yl acetate (**1a**)

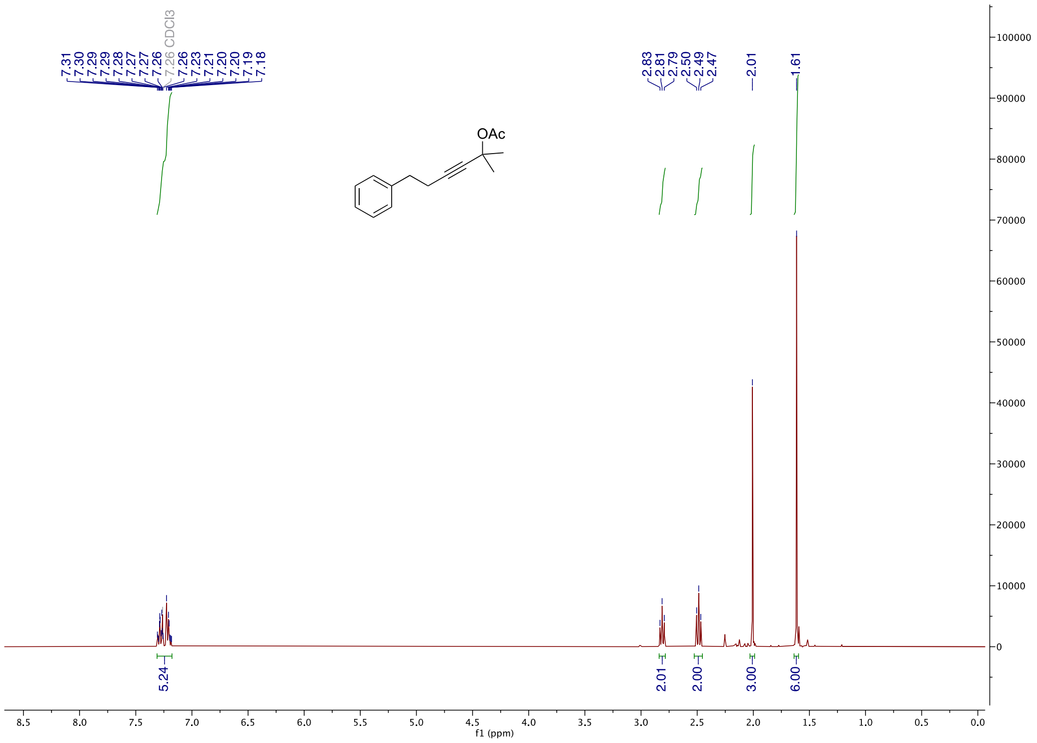

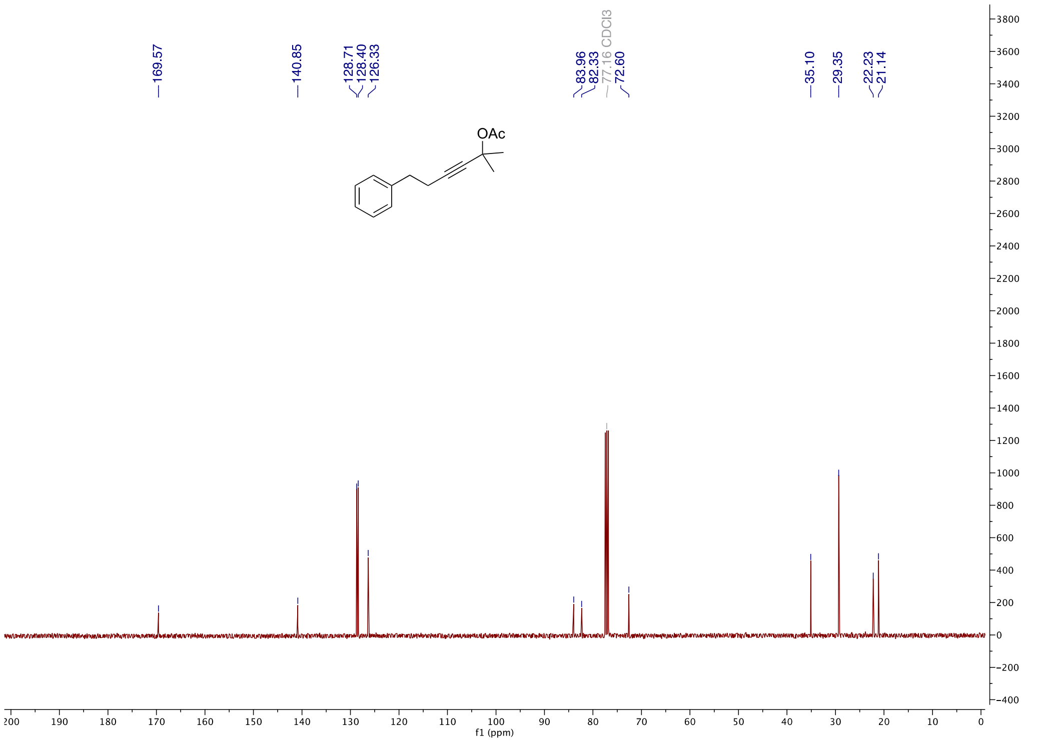

Top: ^{1}H NMR (400 MHz, $CDCl_3$), and bottom: ^{13}C NMR (101 MHz, $CDCl_3$).

2-Methyl-4-phenylbut-3-yn-2-yl acetate (**1b**)

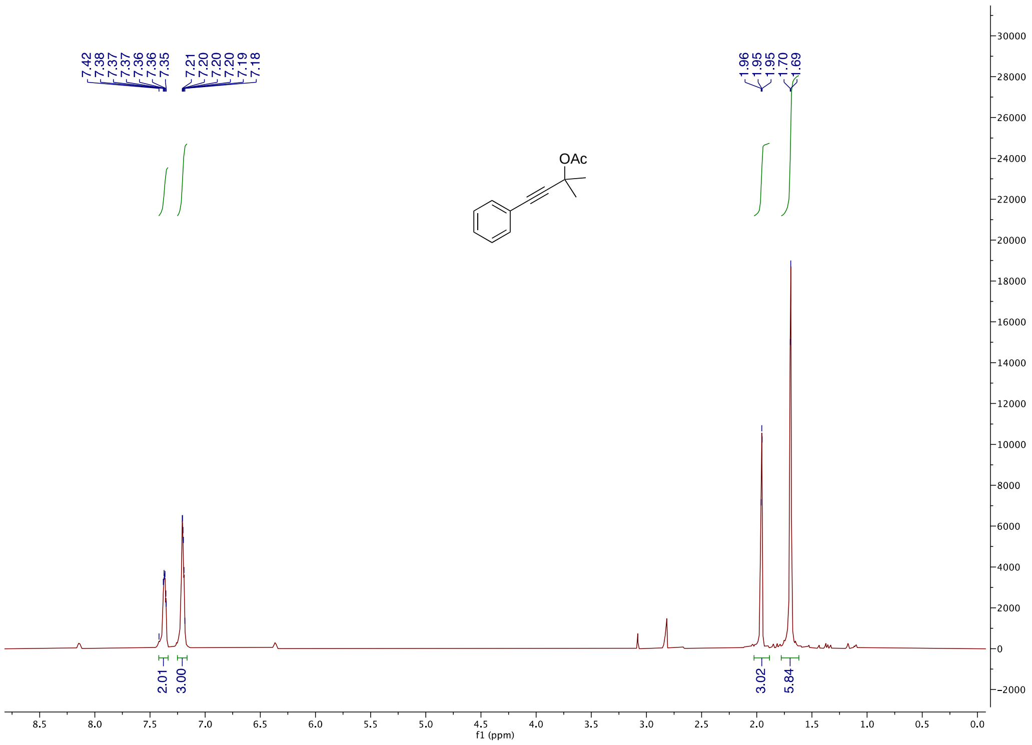

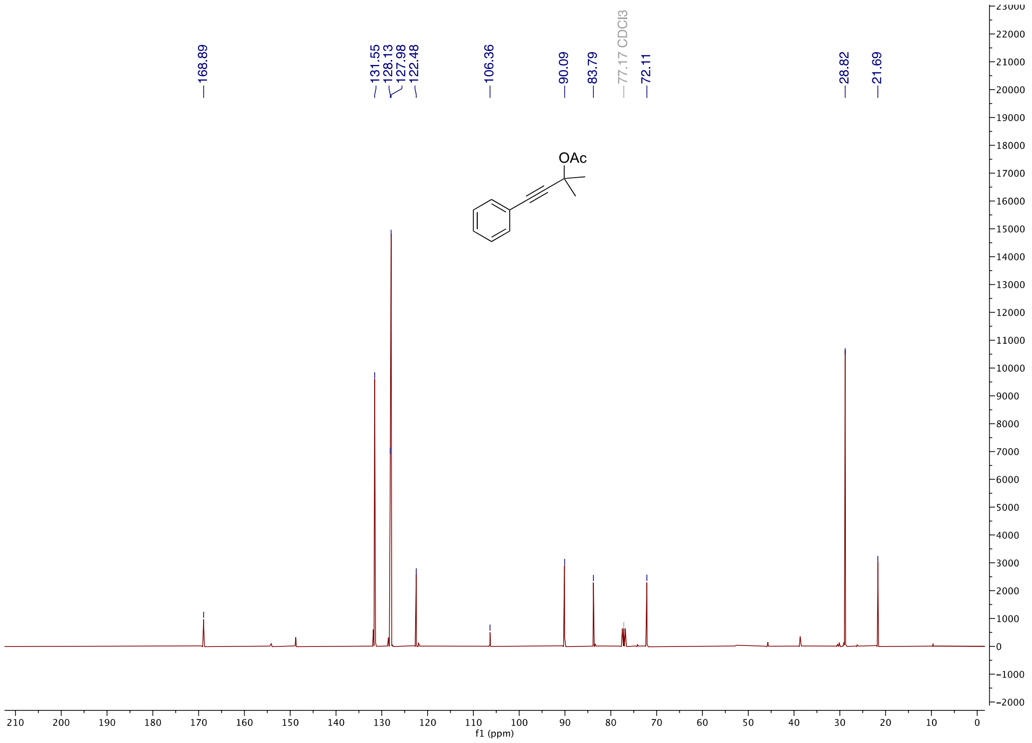

Top: ^{1}H NMR (400 MHz, $CDCl_3$), and bottom: ^{13}C NMR (101 MHz, $CDCl_3$).

2-Methyloct-3-yn-2-yl acetate (**1c**)

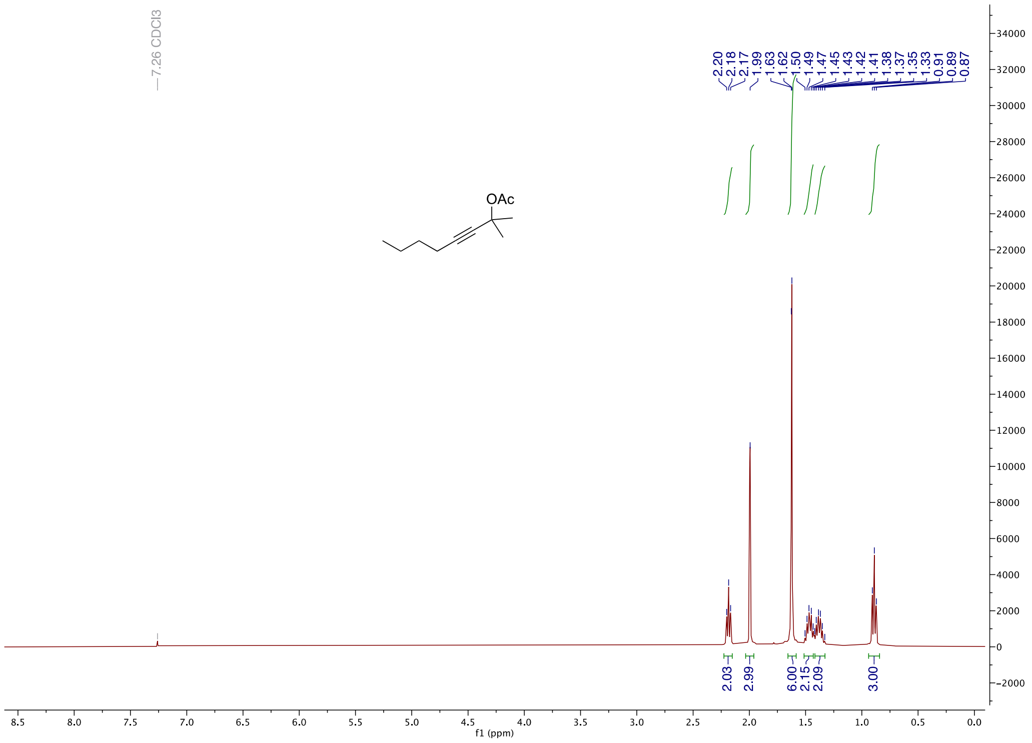

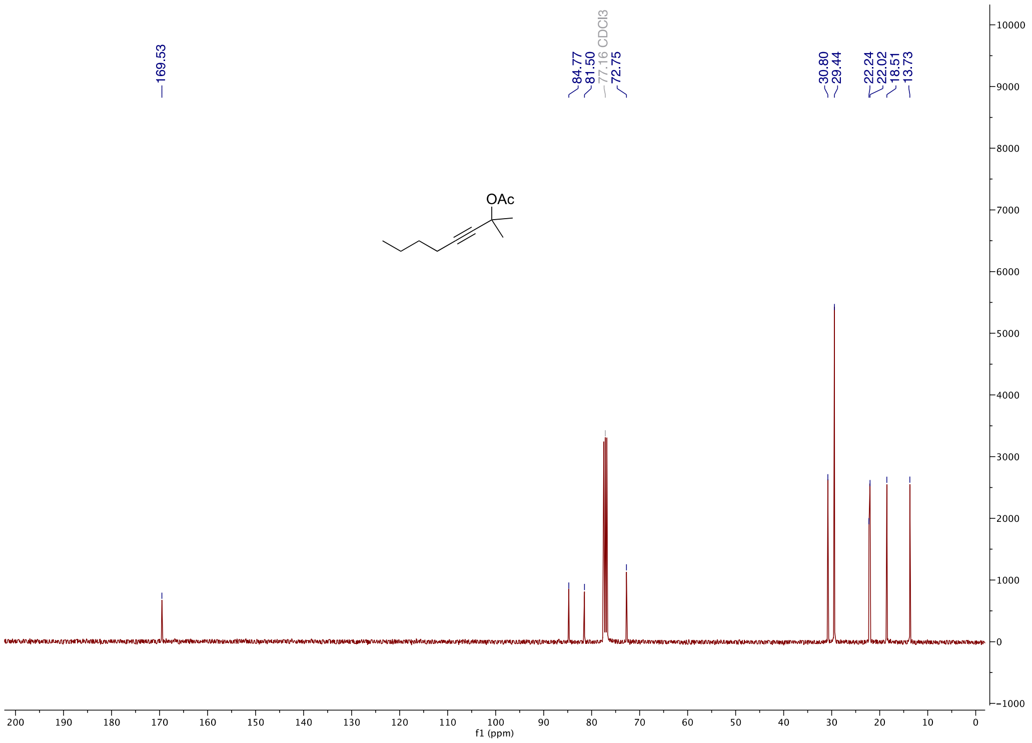

Top: ^{1}H NMR (400 MHz, $CDCl_3$), and bottom: ^{13}C NMR (101 MHz, $CDCl_3$).

1-(Hex-1-yn-1-yl)cyclopentyl acetate (**1d**)

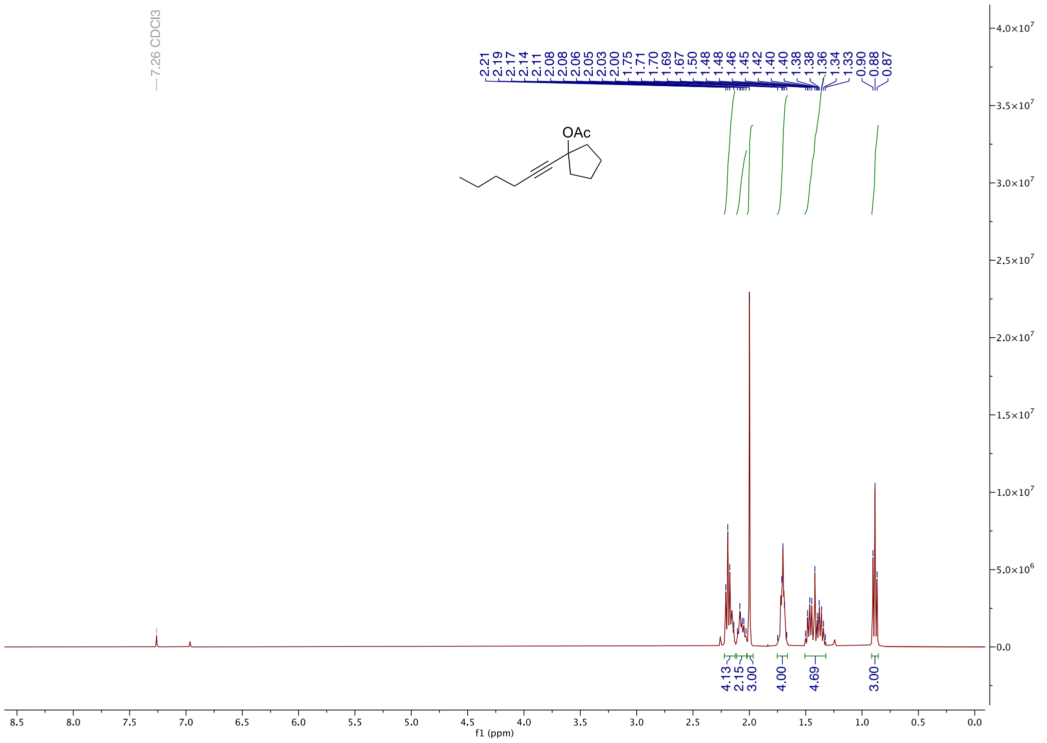

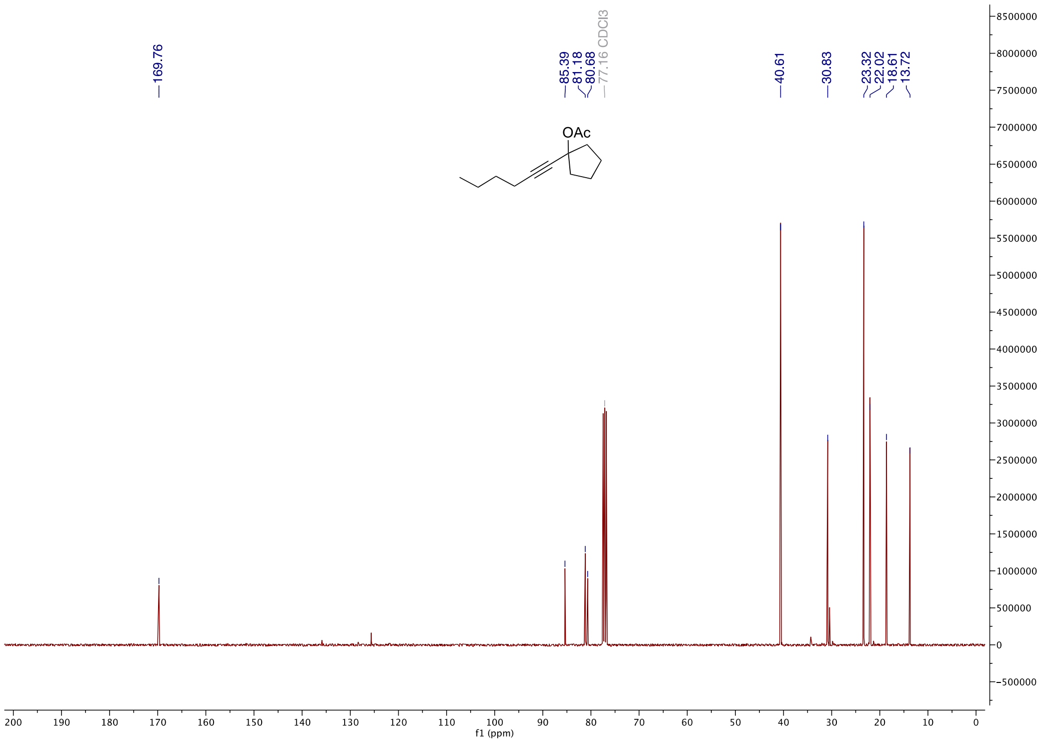

Top: ^{1}H NMR (400 MHz, $CDCl_3$), and bottom: ^{13}C NMR (101 MHz, $CDCl_3$).

1-(Hept-1-yn-1-yl)cyclopentyl acetate (**1e**)

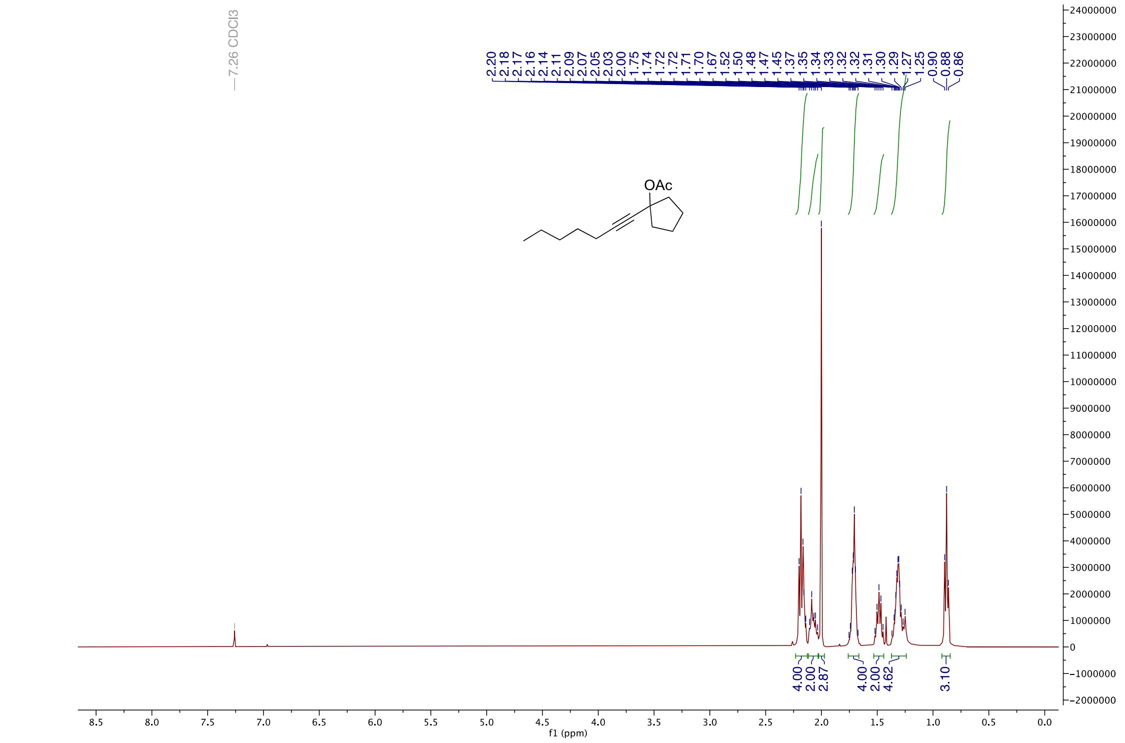

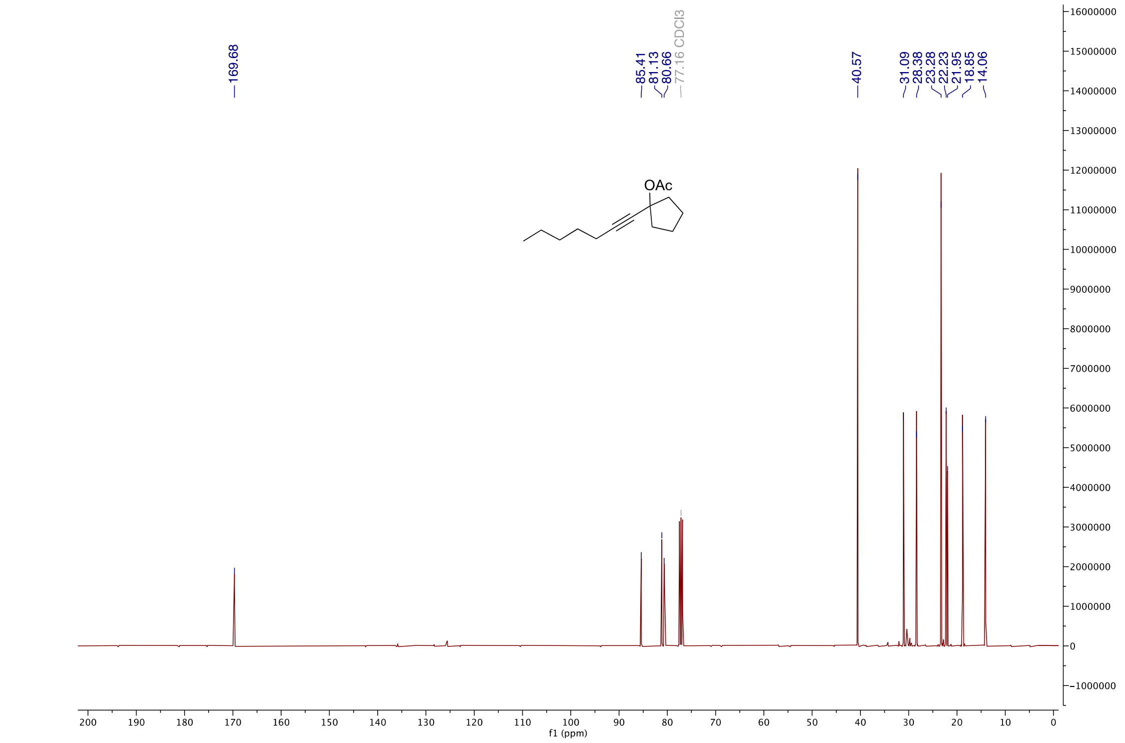

Top: ^{1}H NMR (400 MHz, $CDCl_3$), and bottom: ^{13}C NMR (101 MHz, $CDCl_3$).

1-(Hept-1-yn-1-yl)cyclohexyl acetate (**1f**)

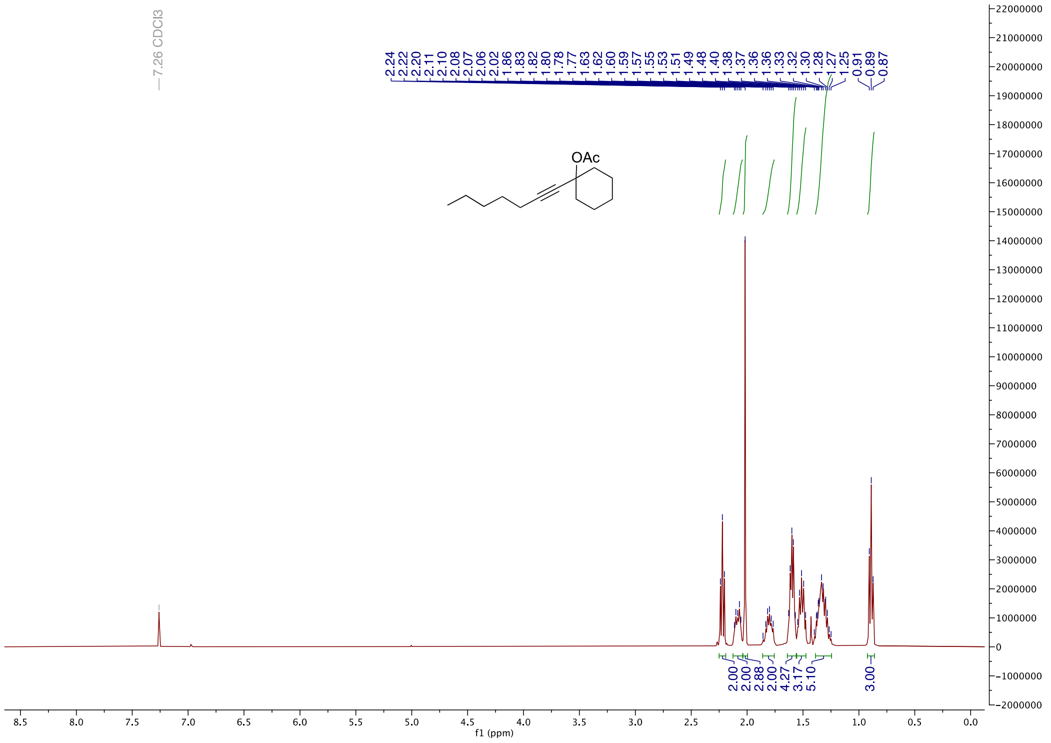

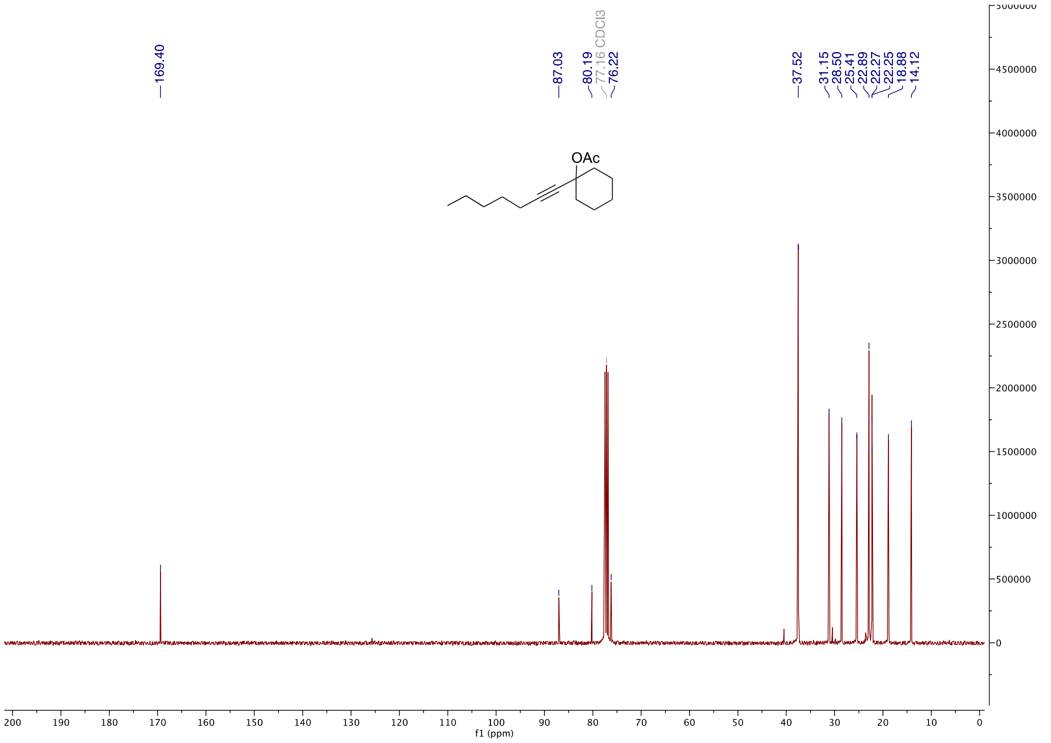

Top: ^{1}H NMR (400 MHz, $CDCl_3$), and bottom: ^{13}C NMR (101 MHz, $CDCl_3$).

6-((tert-Butyldimethylsilyl)oxy)-3-methyl-1-phenylhex-4-yn-3-yl acetate (**1g**)

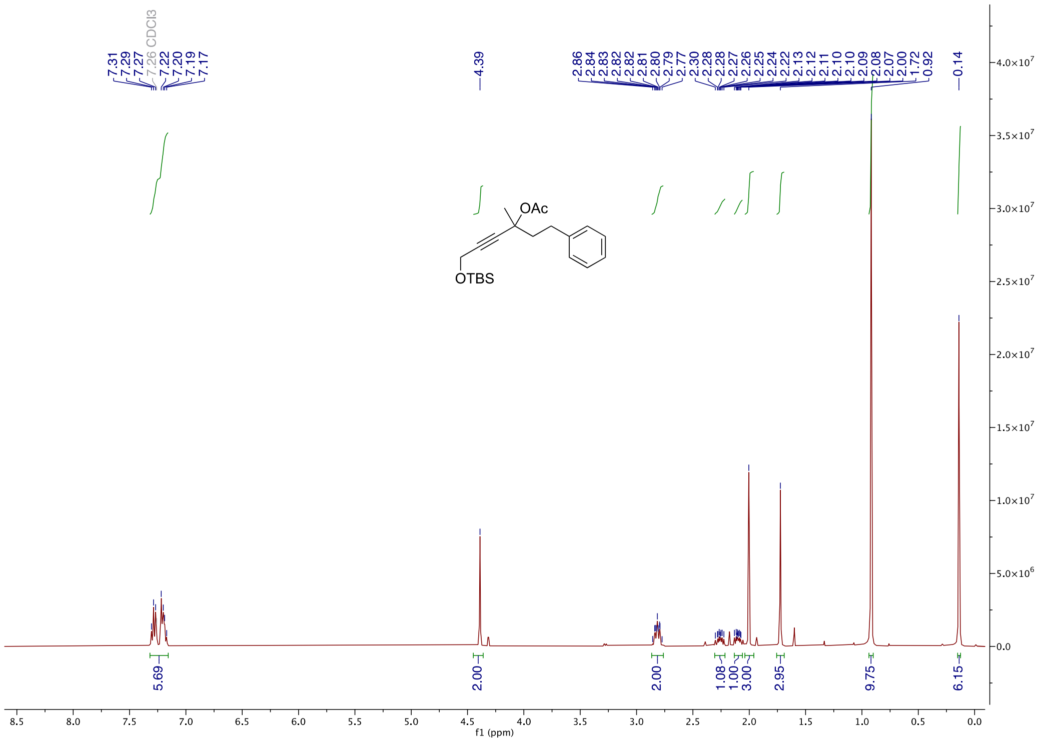

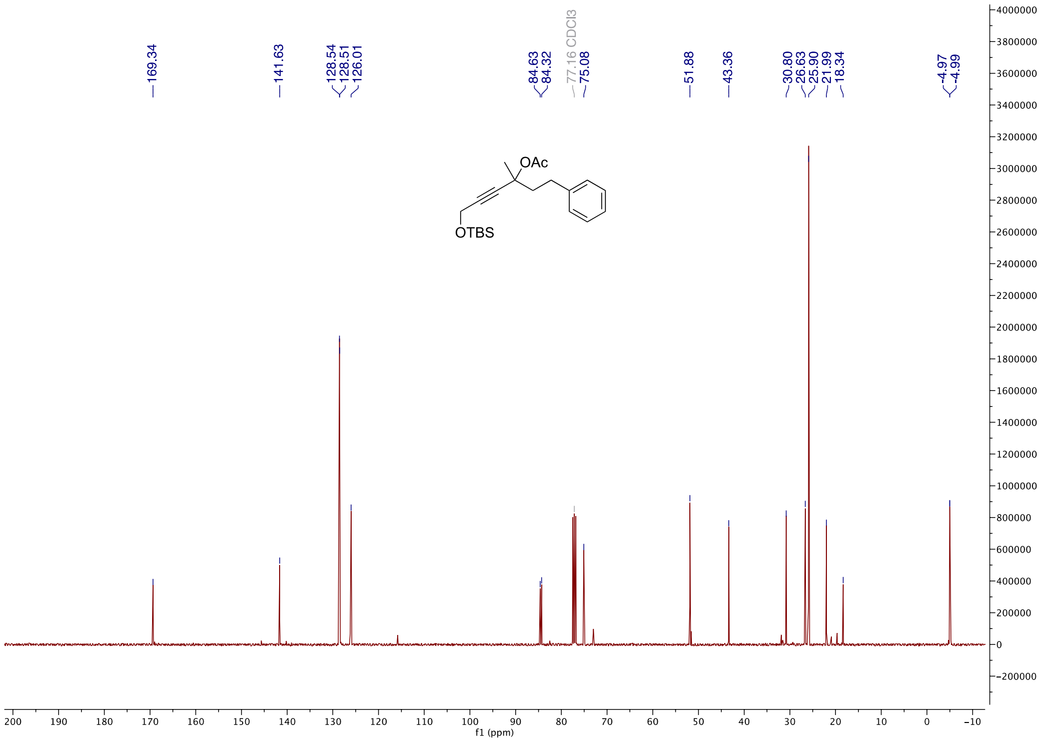

Top: ^{1}H NMR (400 MHz, $CDCl_3$), and bottom: ^{13}C NMR (101 MHz, $CDCl_3$).

1-(4-((tert-Butyldimethylsilyl)oxy)but-1-yn-1-yl)cyclohexyl acetate (**1h**)

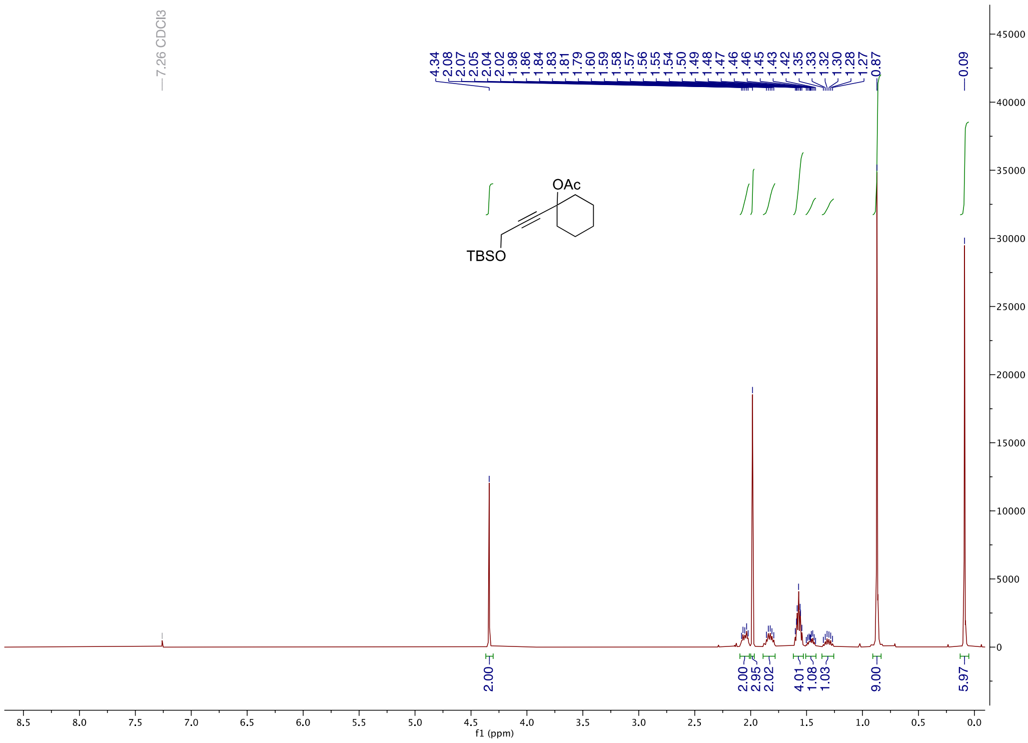

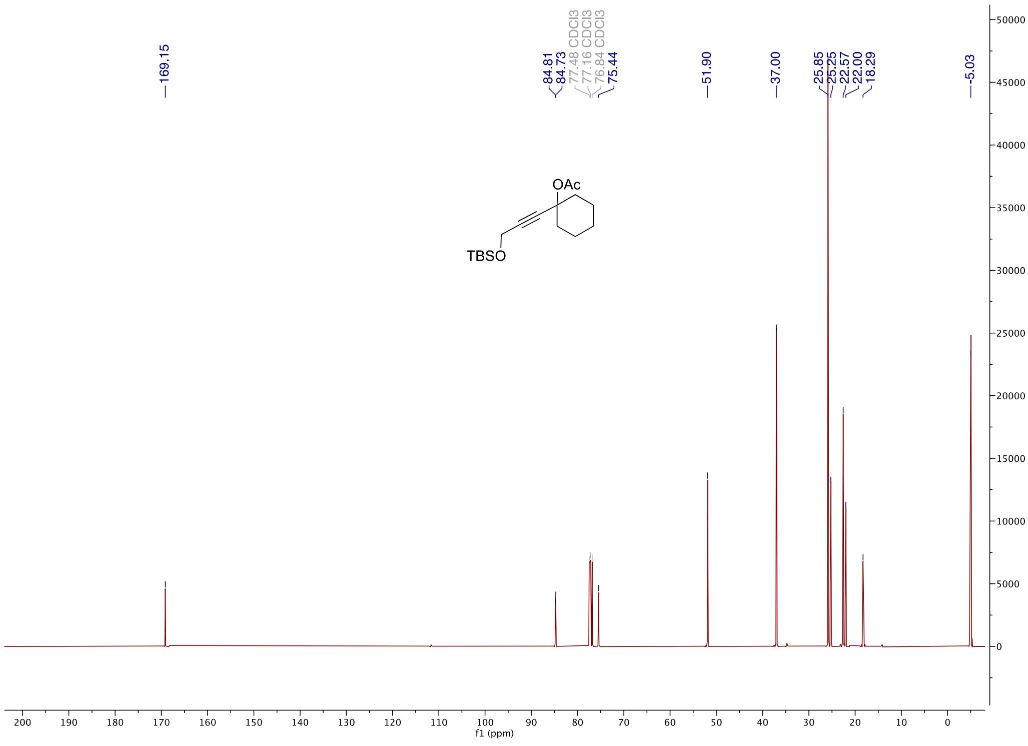

Top: ^{1}H NMR (400 MHz, $CDCl_3$), and bottom: ^{13}C NMR (101 MHz, $CDCl_3$).

7-((tert-Butyldimethylsilyl)oxy)-3-methyl-1-phenylhept-4-yn-3-yl acetate (**1i**)

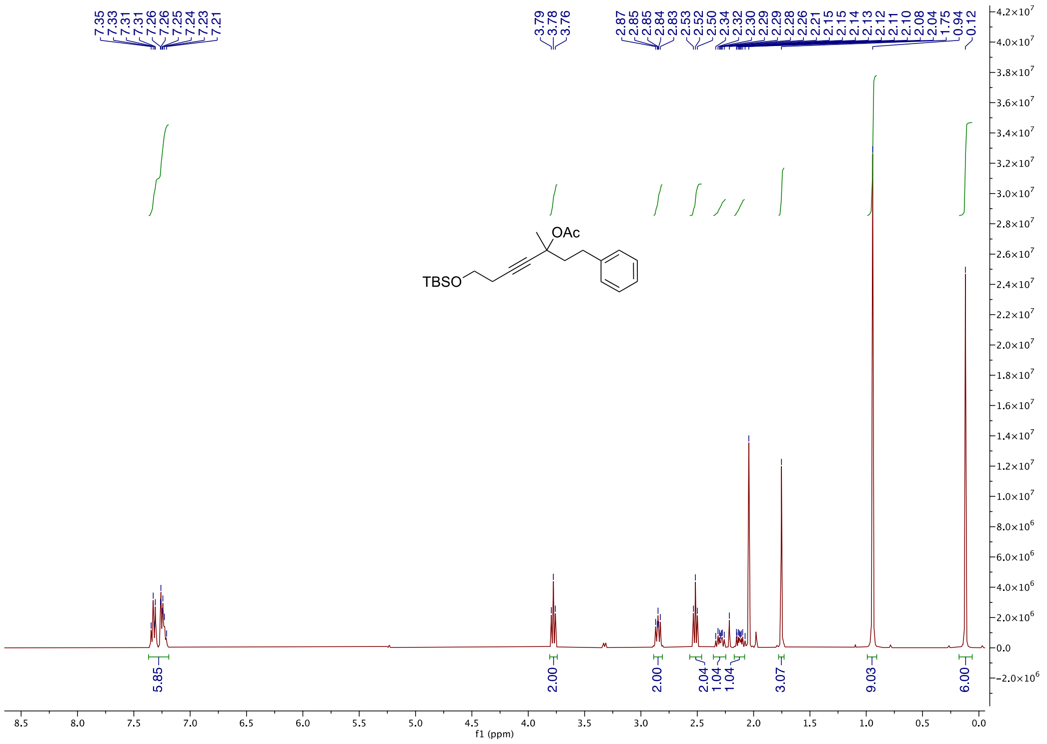

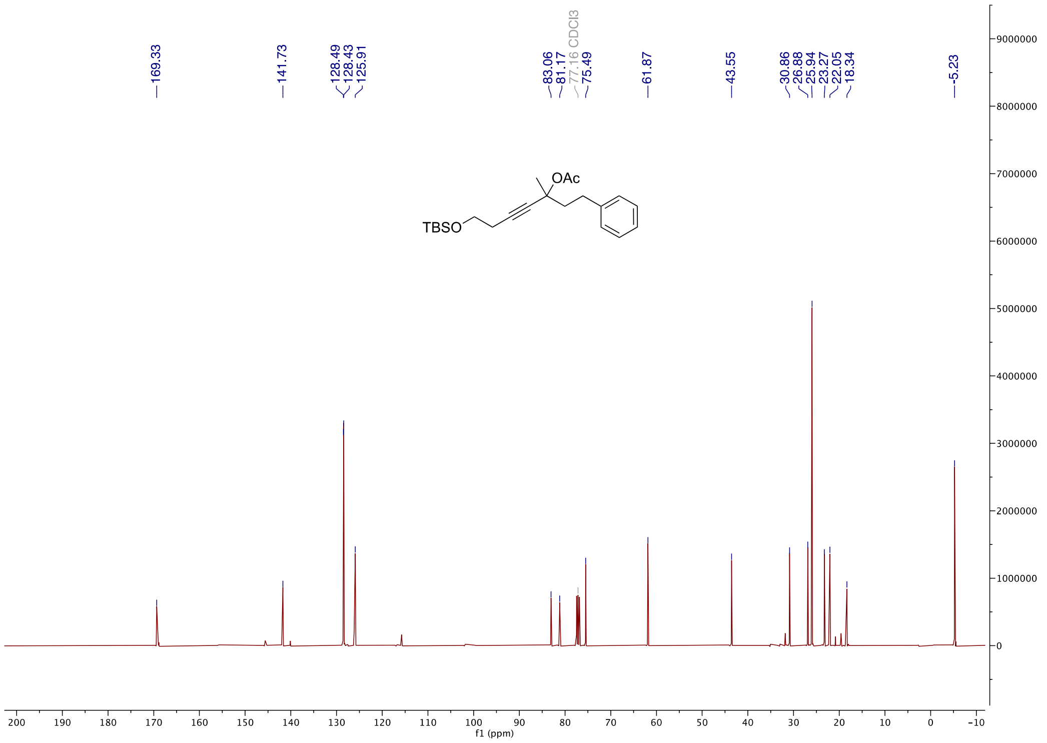

Top: ^{1}H NMR (400 MHz, $CDCl_3$), and bottom: ^{13}C NMR (101 MHz, $CDCl_3$).

tert-Butyl-3-acetoxy-2,2-bis(((tert-butyldimethylsilyl)oxy)methyl)-3-methyl-5-phenylpent-4-ynoate (**1j**)

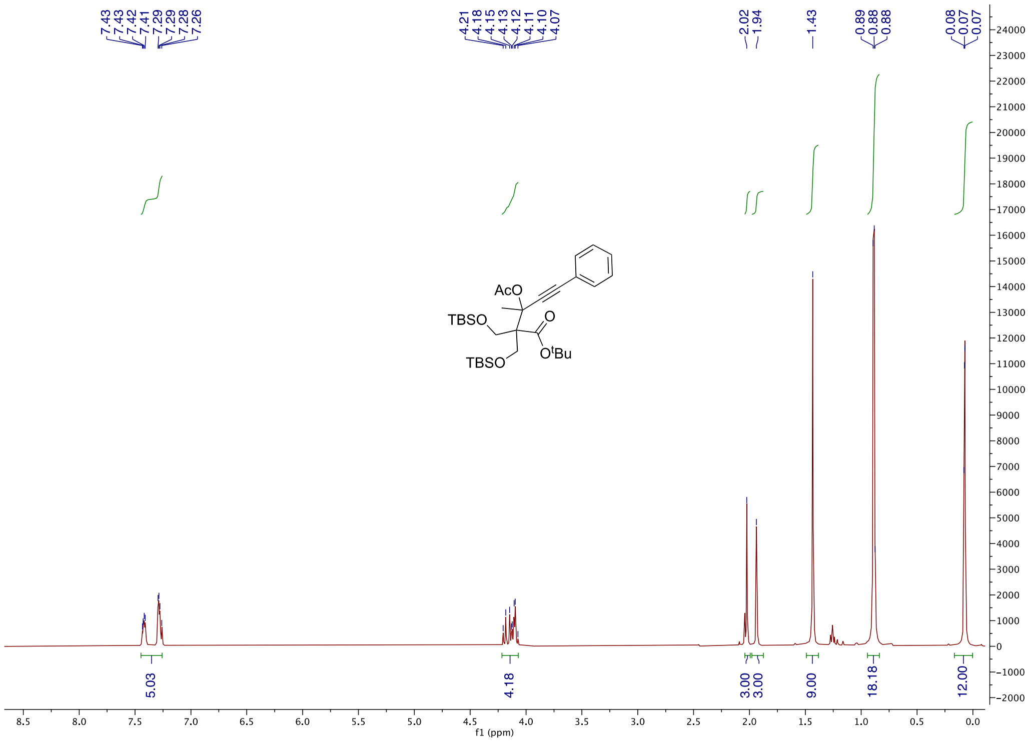

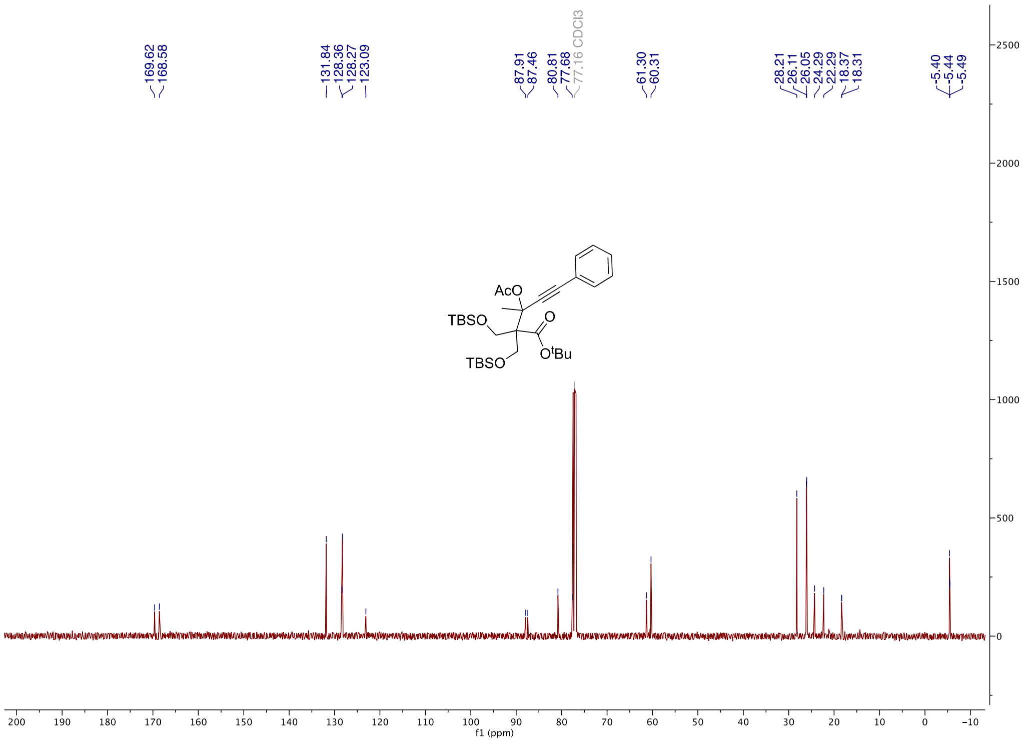

Top: ^{1}H NMR (400 MHz, $CDCl_3$), and bottom: ^{13}C NMR (101 MHz, $CDCl_3$).

2-Methylbut-3-yn-2-yl acetate (**1k**)

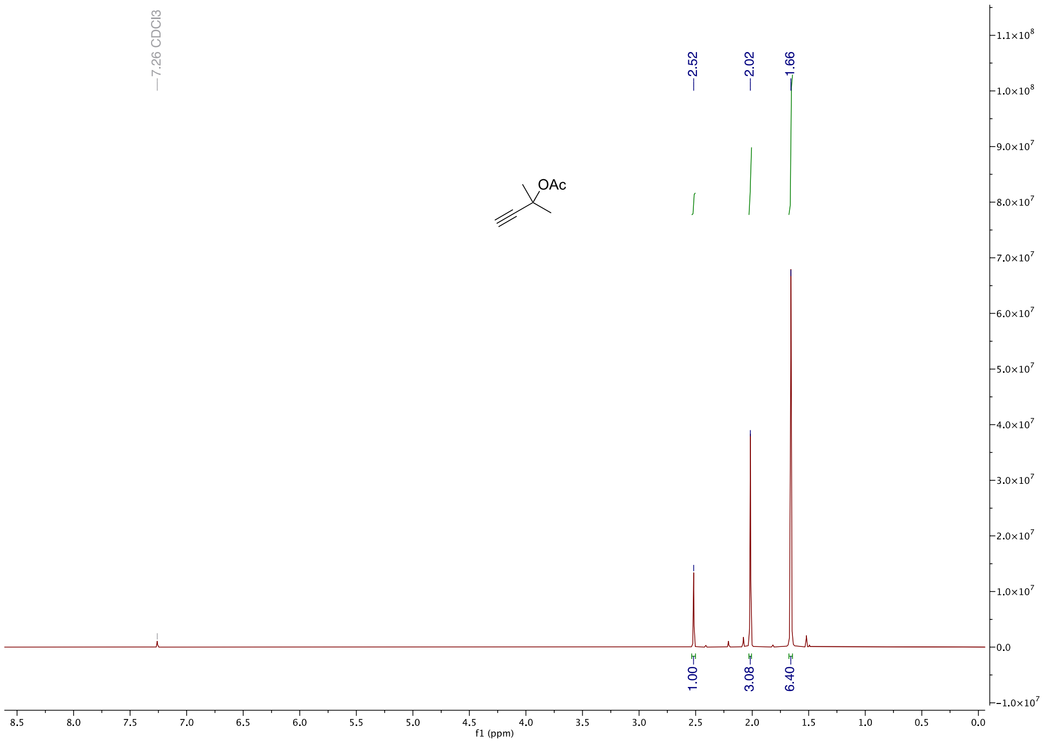

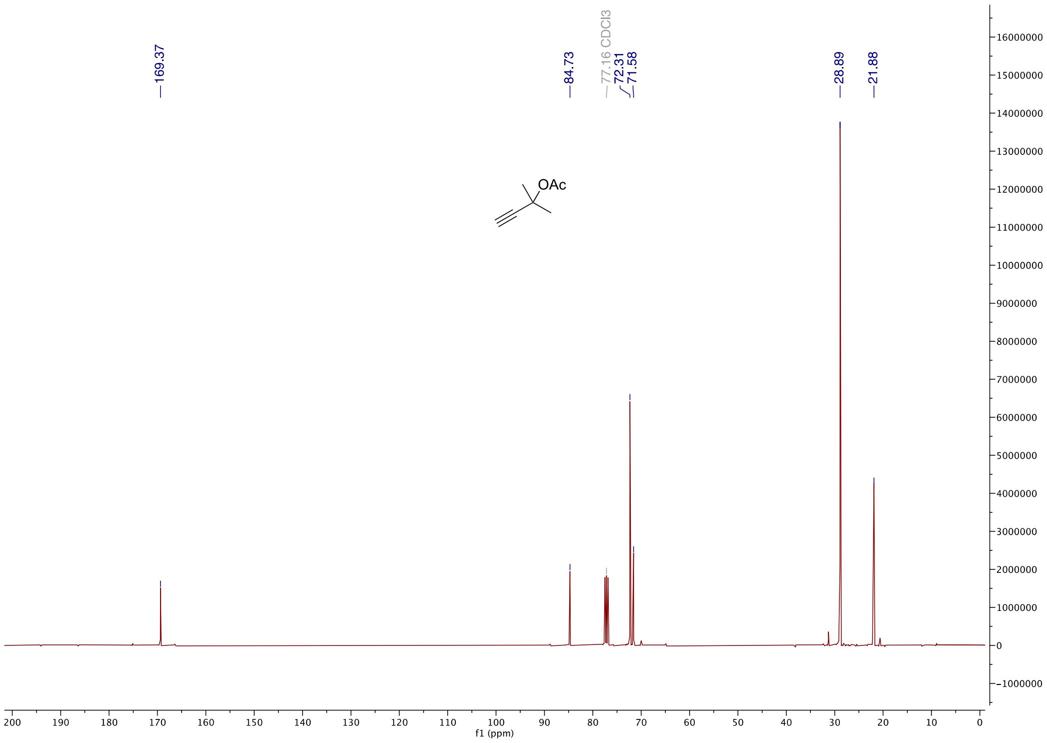

Top: ^{1}H NMR (400 MHz, $CDCl_3$), and bottom: ^{13}C NMR (101 MHz, $CDCl_3$).

3-Ethylpent-1-yn-3-yl acetate (**1l**)

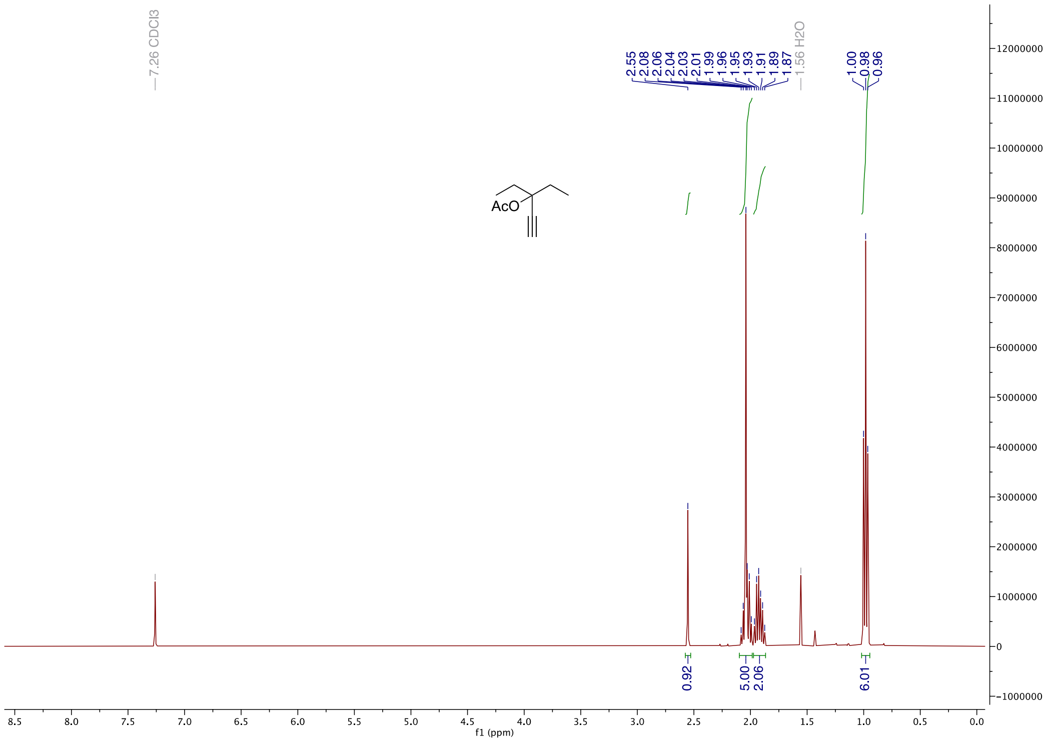

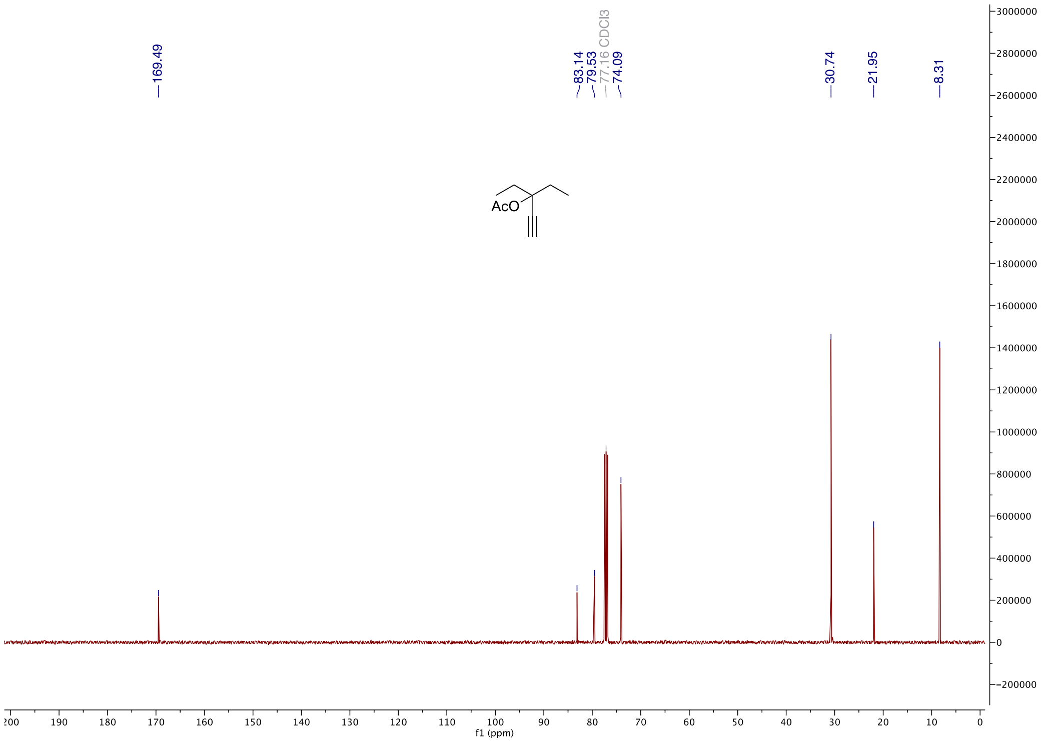

Top: ^{1}H NMR (400 MHz, $CDCl_3$), and bottom: ^{13}C NMR (101 MHz, $CDCl_3$).

1-Ethynylcyclohexyl acetate (**1m**)

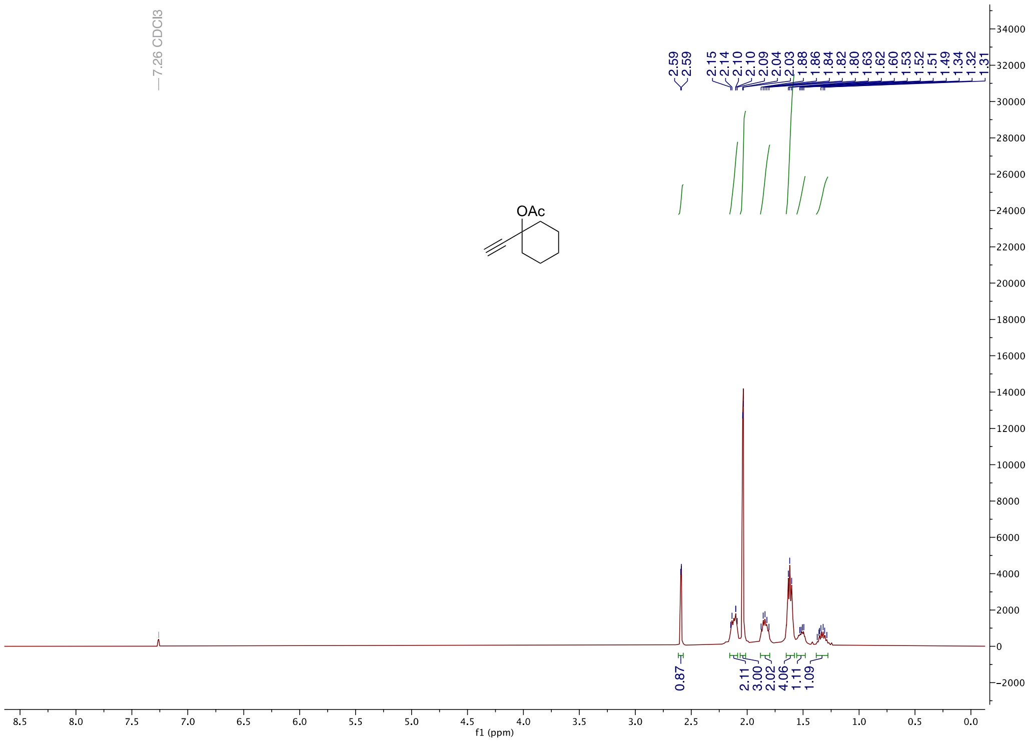

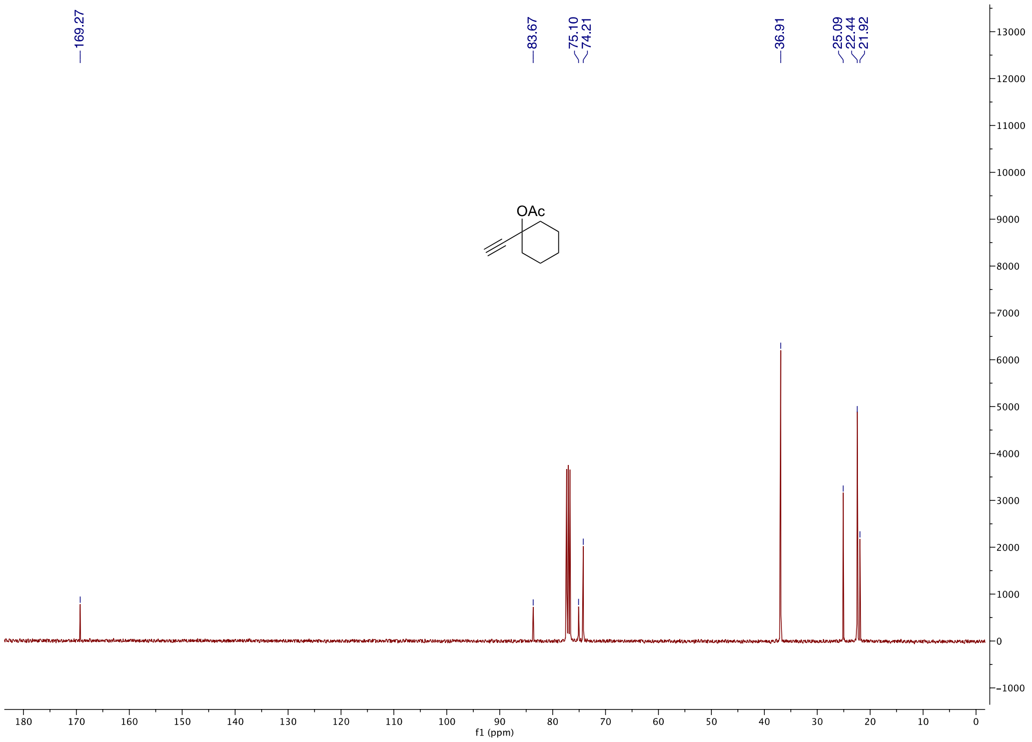

Top: ^{1}H NMR (400 MHz, $CDCl_3$), and bottom: ^{13}C NMR (101 MHz, $CDCl_3$).

4-Phenylbut-3-yn-2-yl acetate (**1n**)

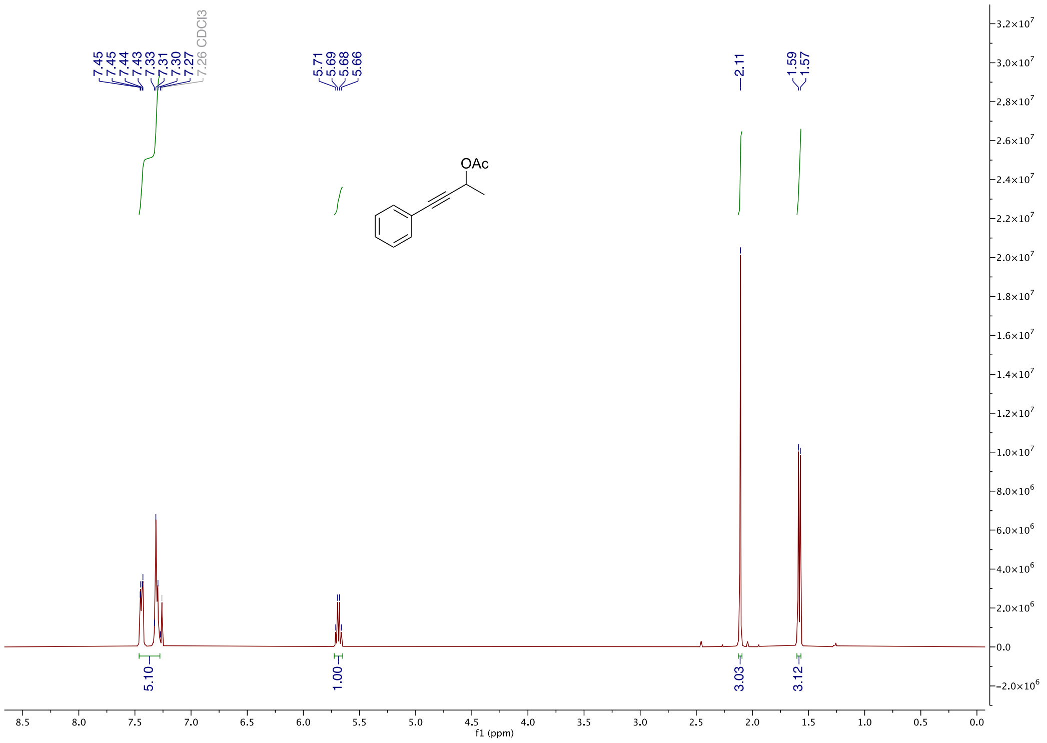

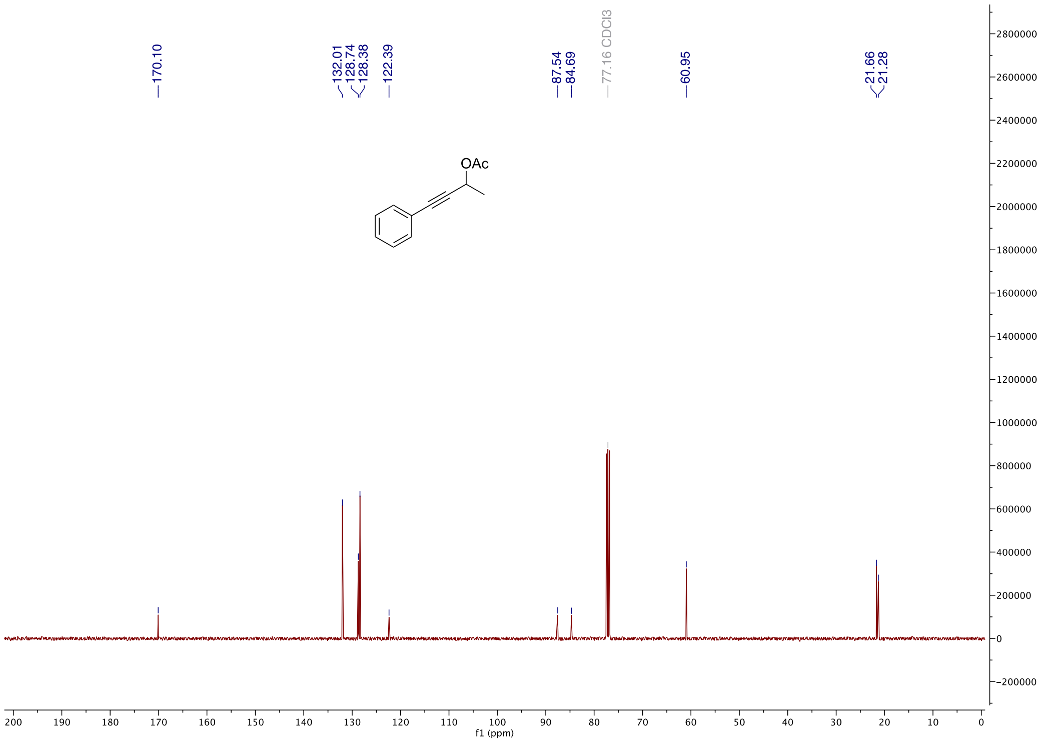

Top: ^{1}H NMR (400 MHz, $CDCl_3$), and bottom: ^{13}C NMR (101 MHz, $CDCl_3$).

1-Phenylpent-1-yn-3-yl acetate (**1o**)

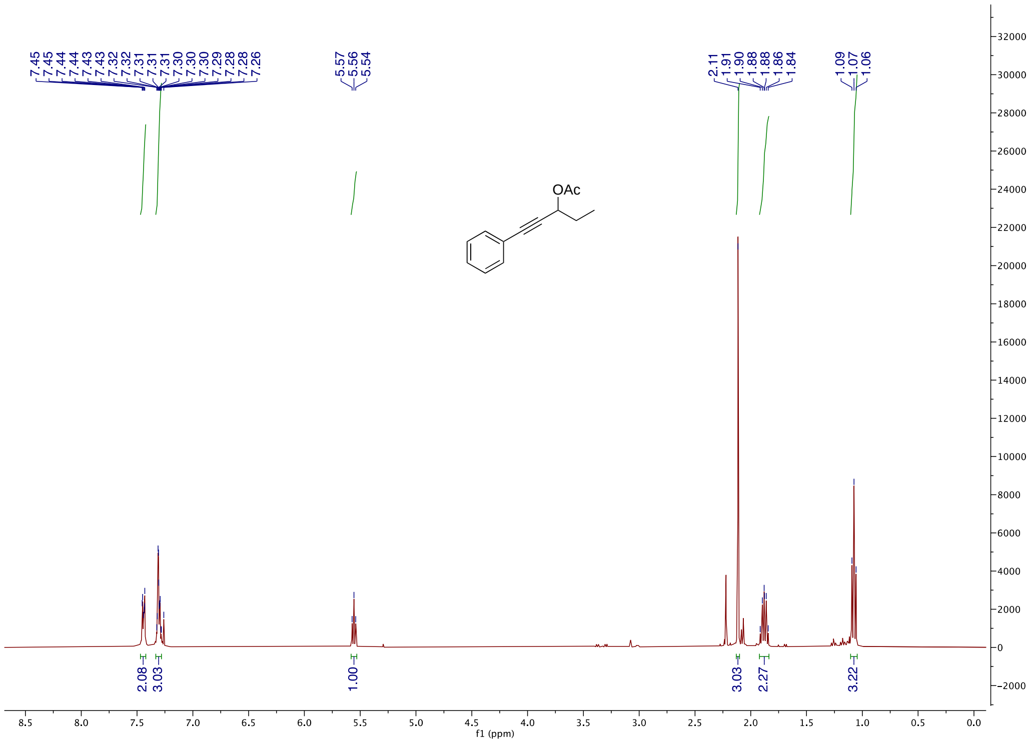

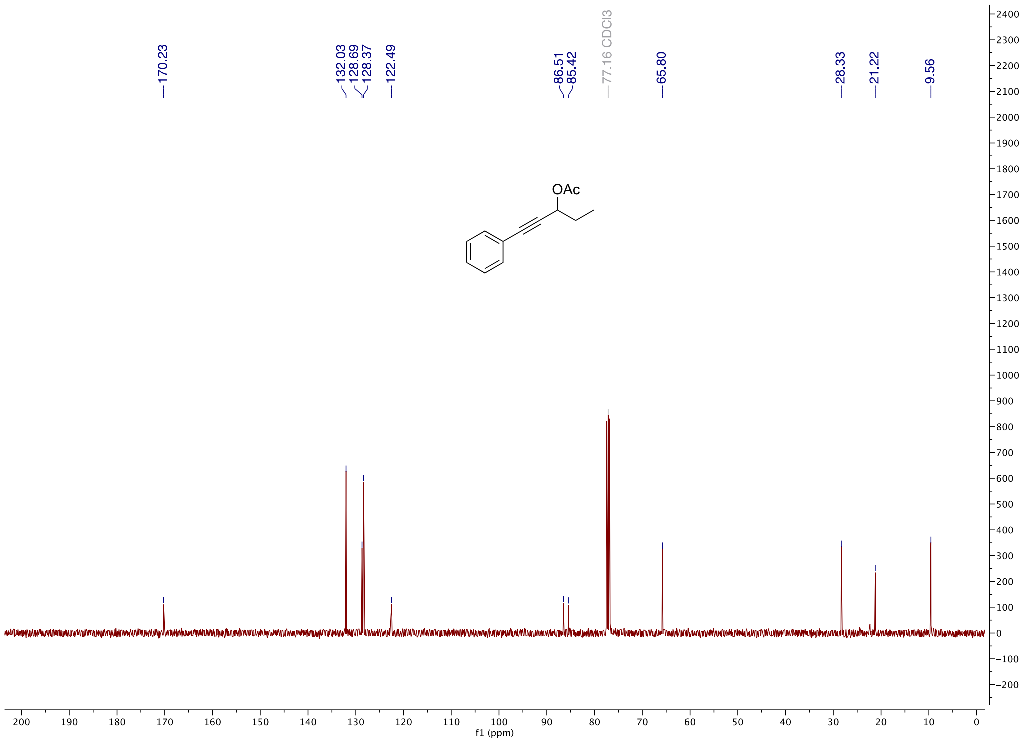

Top: ^{1}H NMR (400 MHz, $CDCl_3$), and bottom: ^{13}C NMR (101 MHz, $CDCl_3$).

2-Phenylbut-3-yn-2-yl acetate (**1p**)

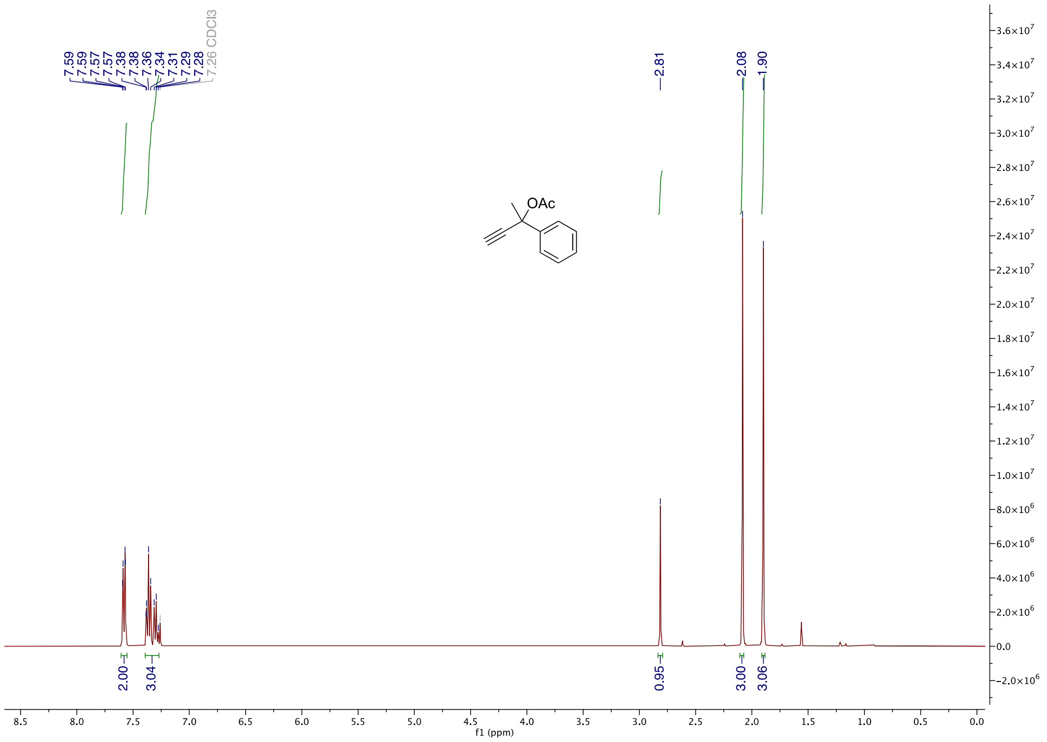

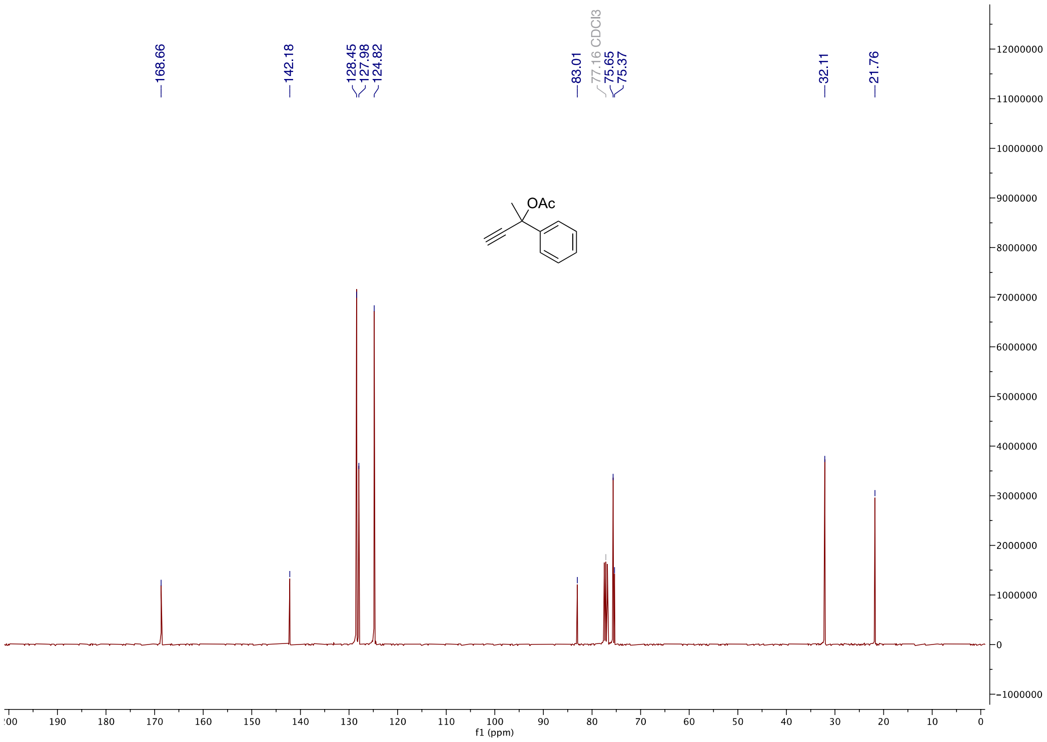

Top: ^{1}H NMR (400 MHz, $CDCl_3$), and bottom: ^{13}C NMR (101 MHz, $CDCl_3$).

1-((tert-Butyldimethylsilyl)oxy)-2-methylbut-3-yn-2-yl acetate (**1q**)

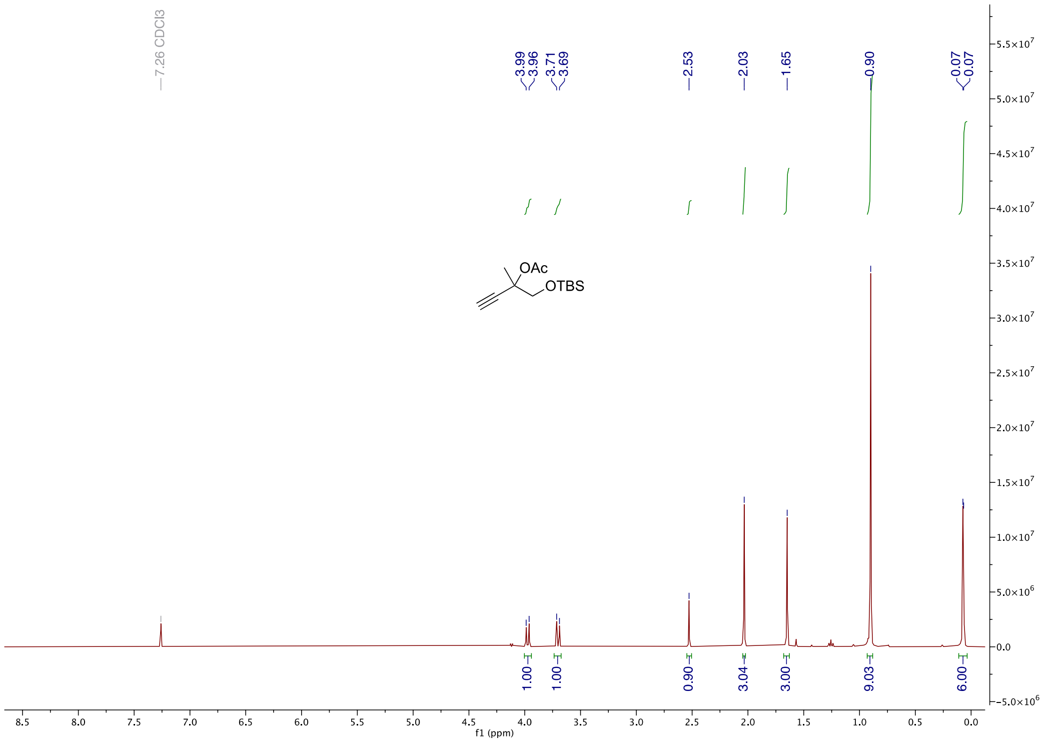

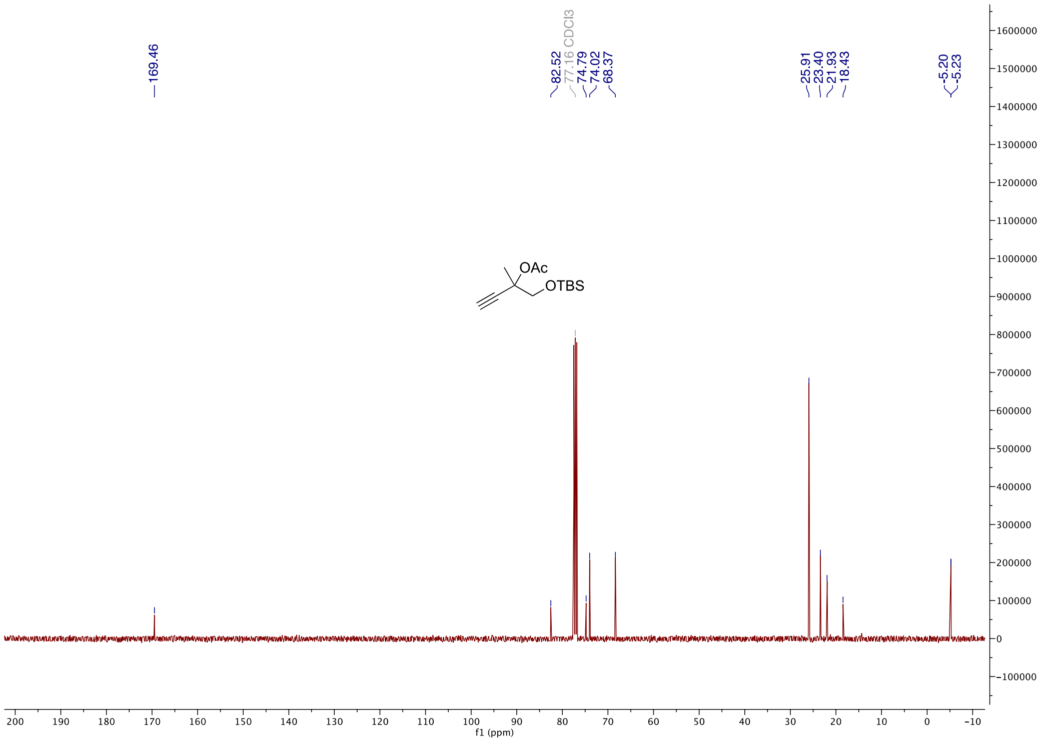

Top: ^{1}H NMR (400 MHz, $CDCl_3$), and bottom: ^{13}C NMR (101 MHz, $CDCl_3$).

Oct-1-yn-3-yl acetate (**1r**)

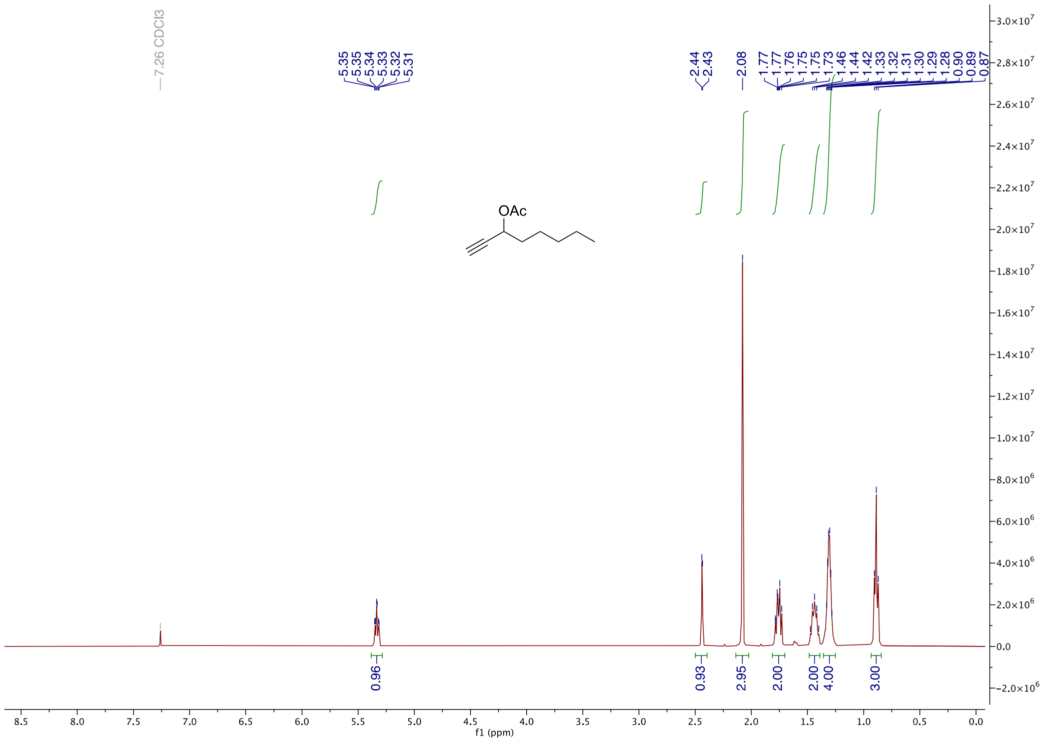

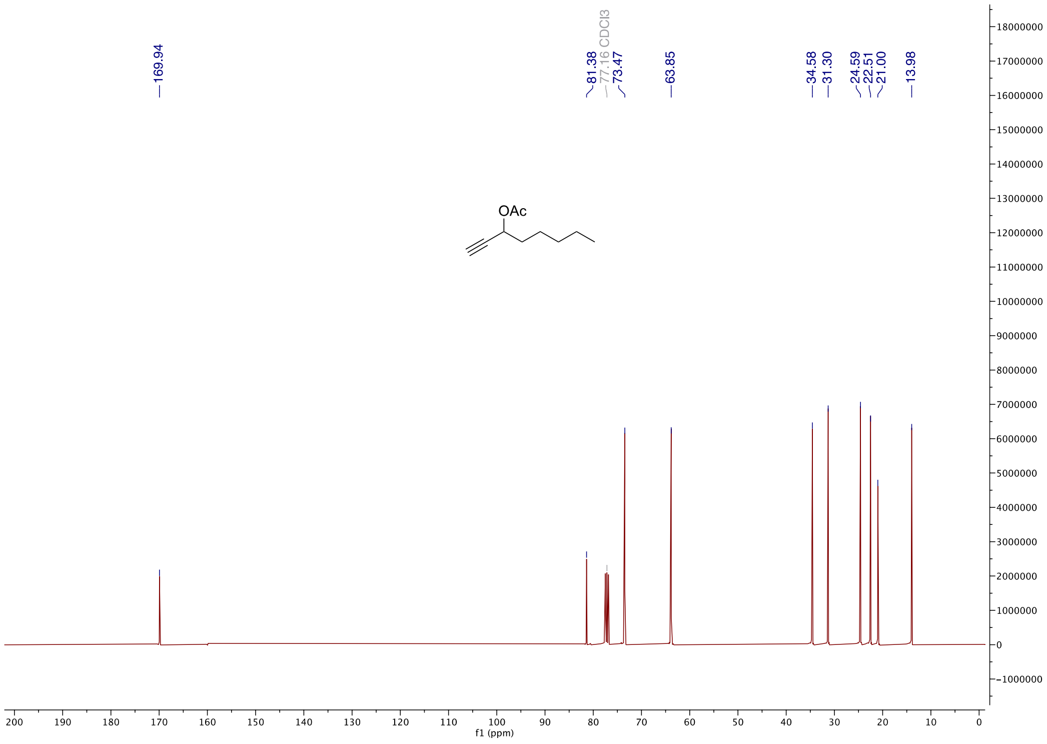

Top: ^{1}H NMR (400 MHz, $CDCl_3$), and bottom: ^{13}C NMR (101 MHz, $CDCl_3$).

(3S,8R,10R,13S,14S)-17-(3-((tert-Butyldimethylsilyl)oxy)prop-1-yn-1-yl)-10,13-dimethyl-2,3,4,7,8,9,10,11,12,13,14,15,16,17-tetradecahydro-1H-cyclopenta[a]phenanthrene-3,17-diyl diacetate (**1s**)

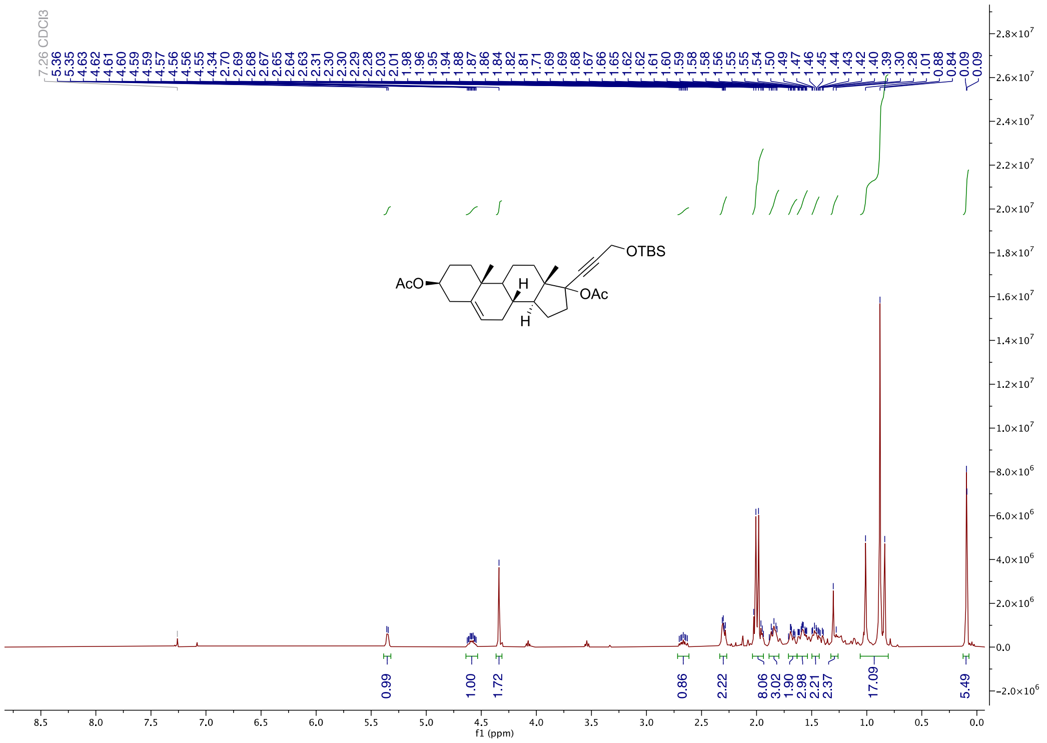

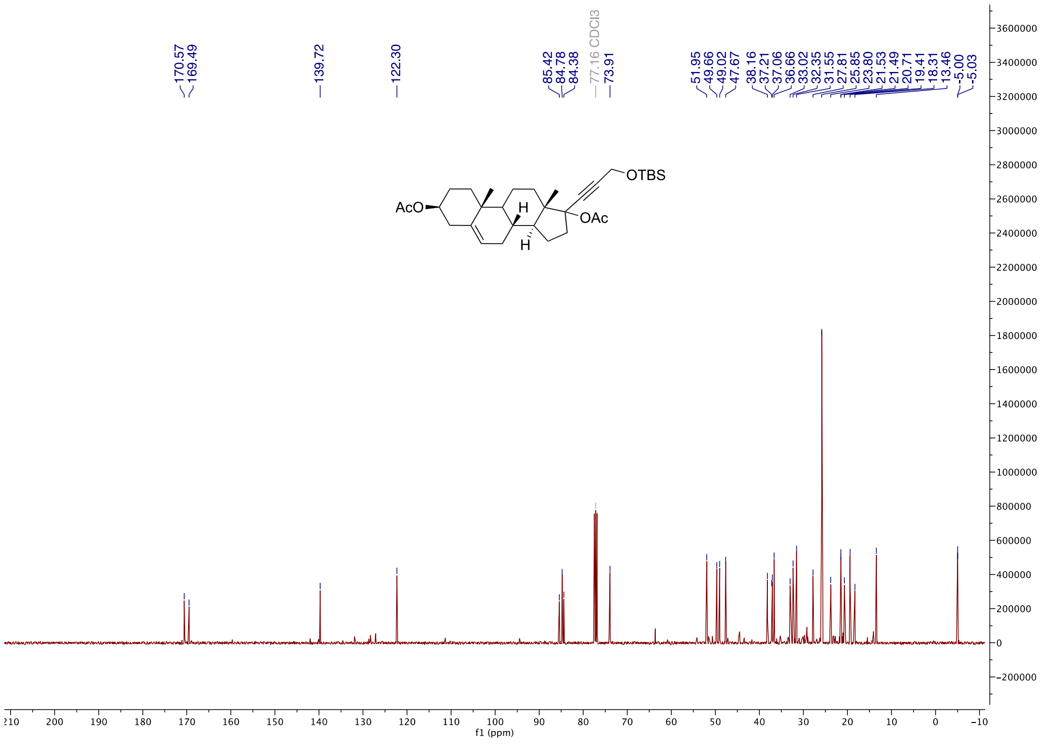

Top: ^{1}H NMR (400 MHz, $CDCl_3$), and bottom: ^{13}C NMR (101 MHz, $CDCl_3$).

4,4,5,5-Tetramethyl-2-(5-methyl-1-phenylhexa-3,4-dien-3-yl)-1,3,2-dioxaborolane (**2a**)

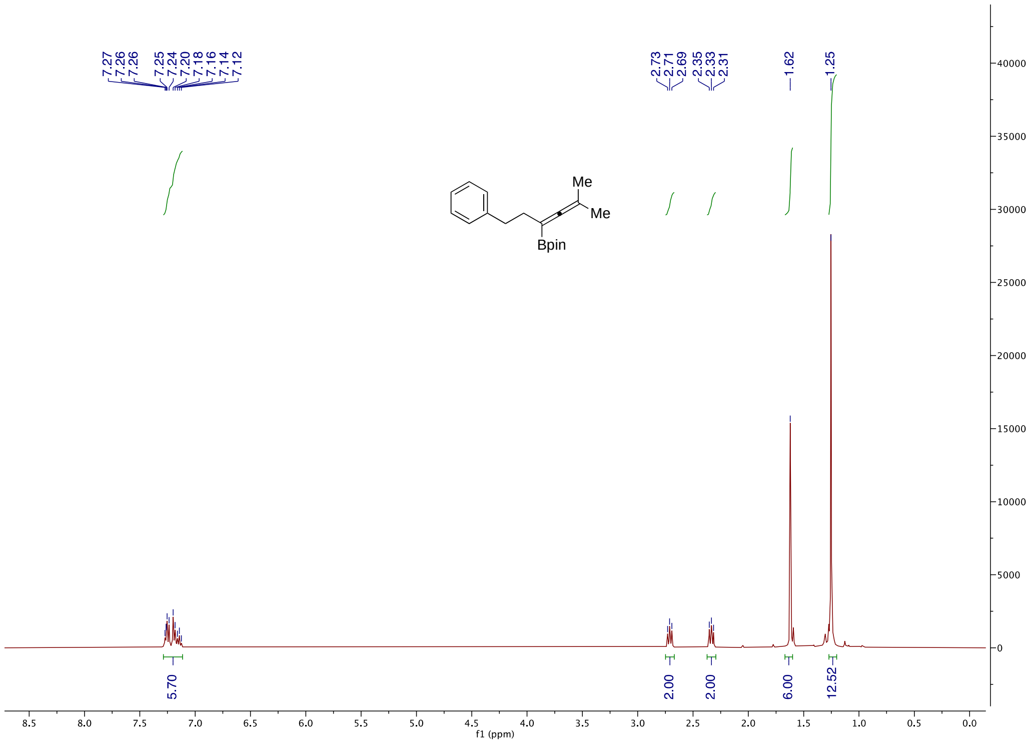

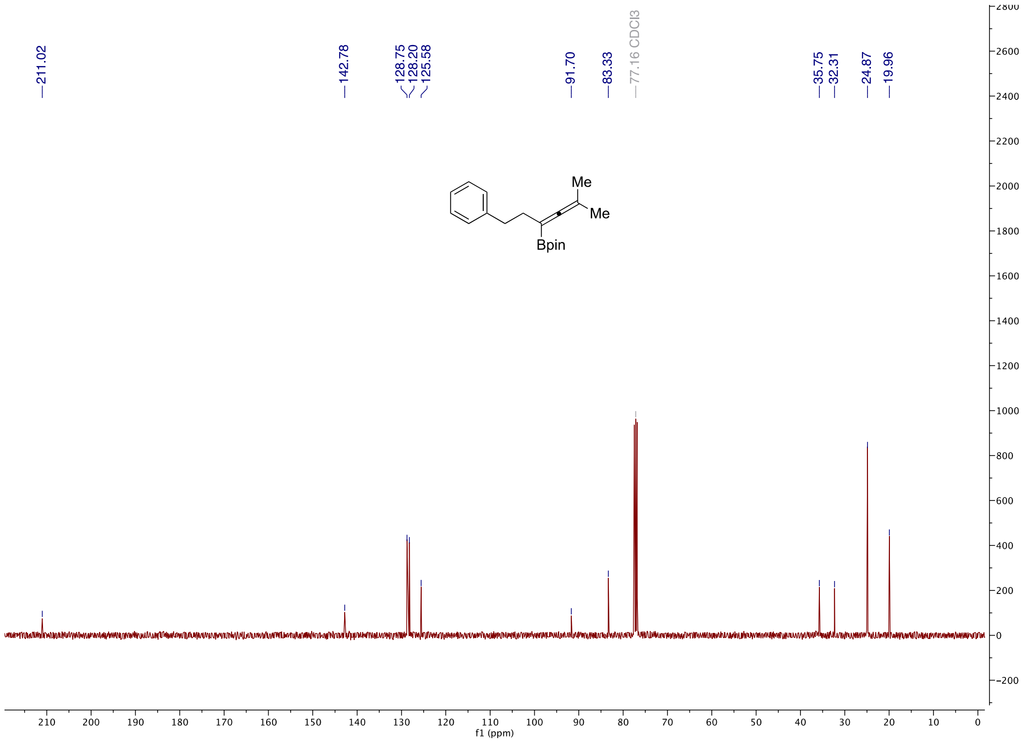

Top: ^{1}H NMR (400 MHz, $CDCl_3$), and bottom: ^{13}C NMR (101 MHz, $CDCl_3$).

4,4,5,5-Tetramethyl-2-(3-methyl-1-phenylbuta-1,2-dien-1-yl)-1,3,2-dioxaborolane (**2b**)

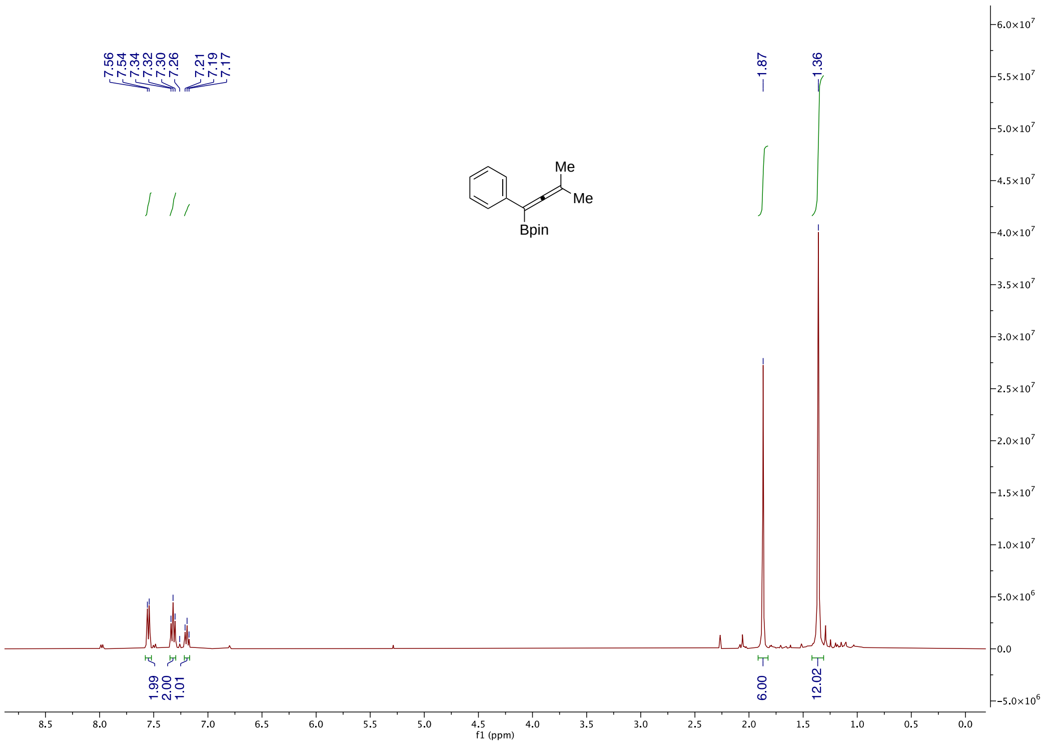

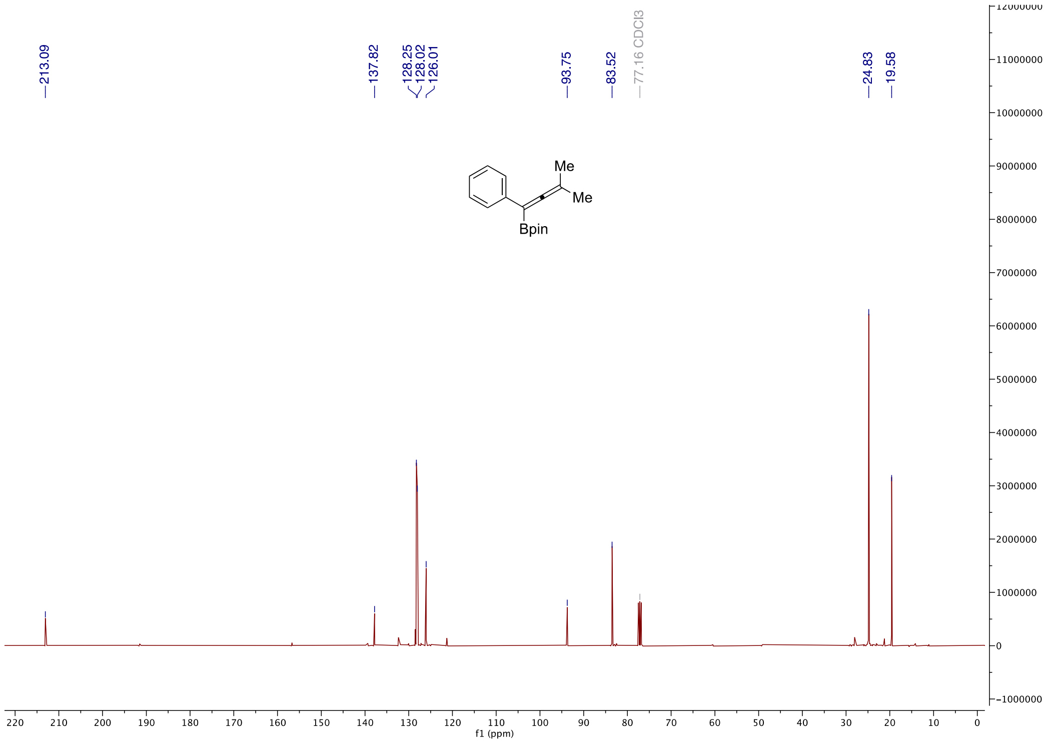

Top: ^{1}H NMR (400 MHz, $CDCl_3$), and bottom: ^{13}C NMR (101 MHz, $CDCl_3$).

4,4,5,5-Tetramethyl-2-(2-methylocta-2,3-dien-4-yl)-1,3,2-dioxaborolane (**2c**)

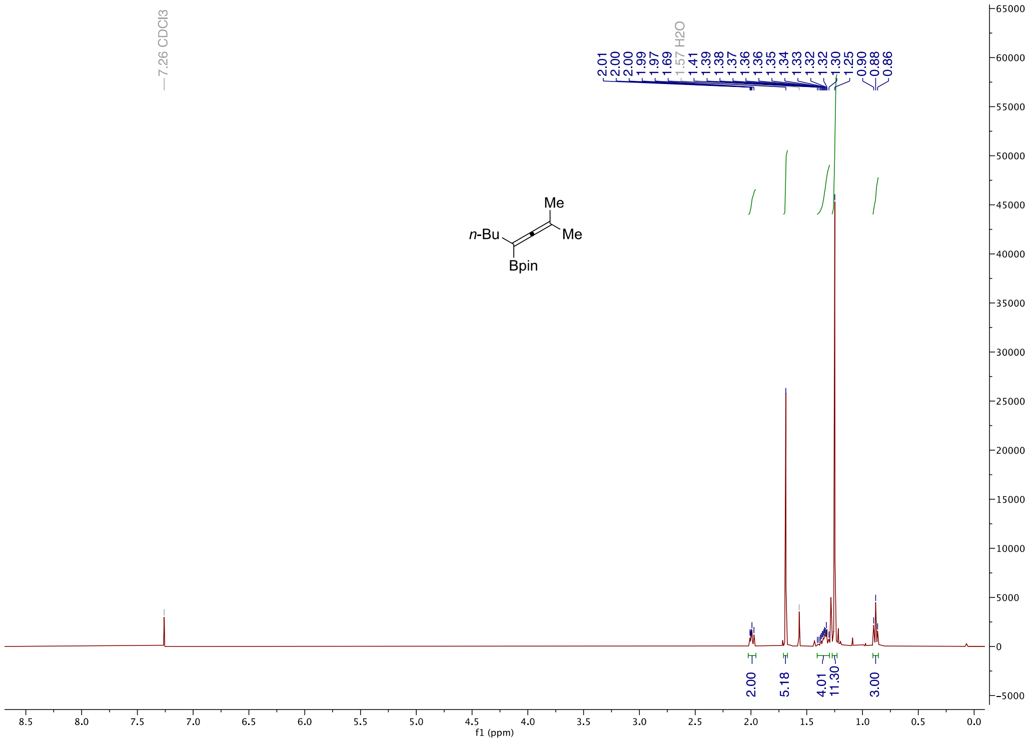

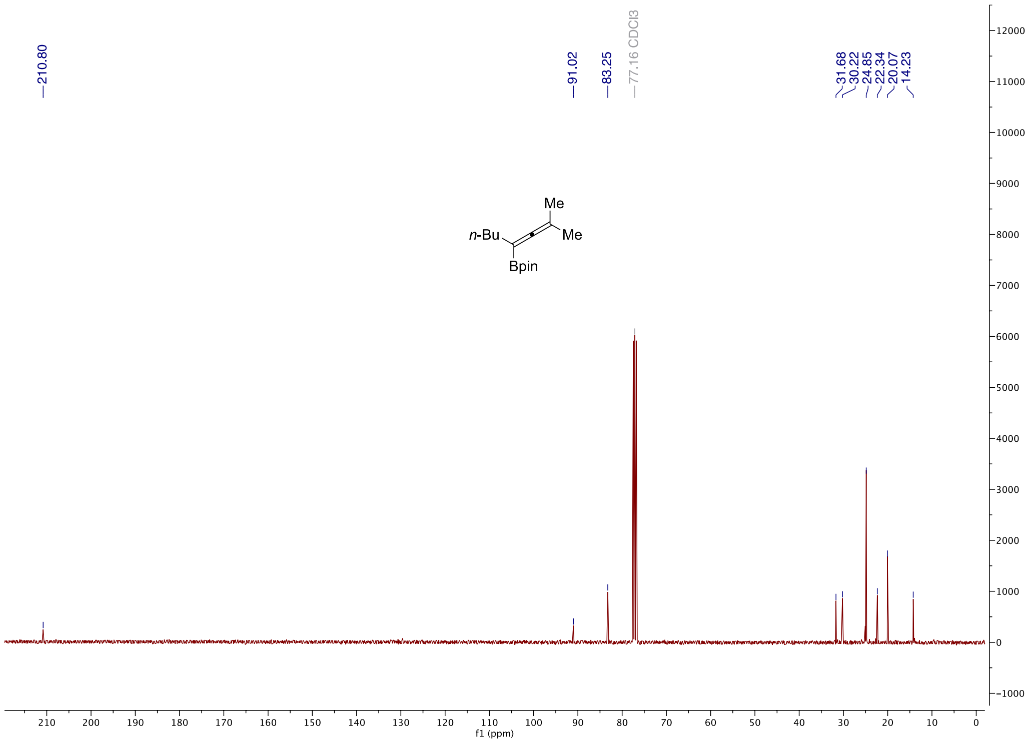

Top: ^{1}H NMR (400 MHz, $CDCl_3$), and bottom: ^{13}C NMR (101 MHz, $CDCl_3$).

2-(1-Cyclopentylidenehex-1-en-2-yl)-4,4,5,5-tetramethyl-1,3,2-dioxaborolane (**2d**)

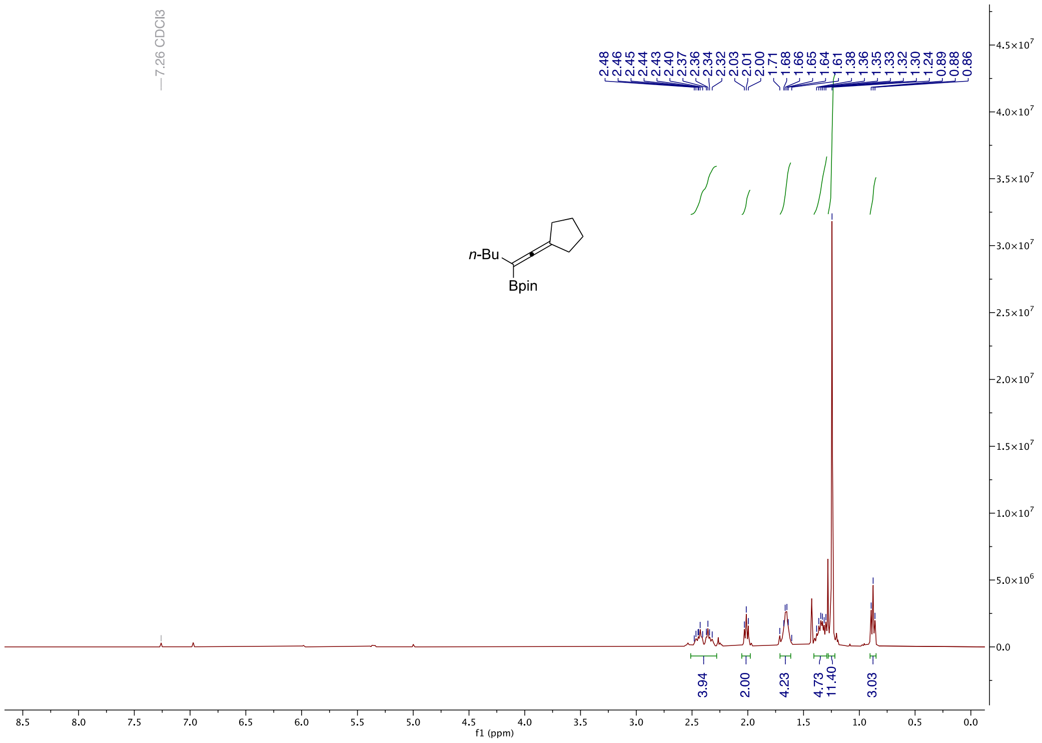

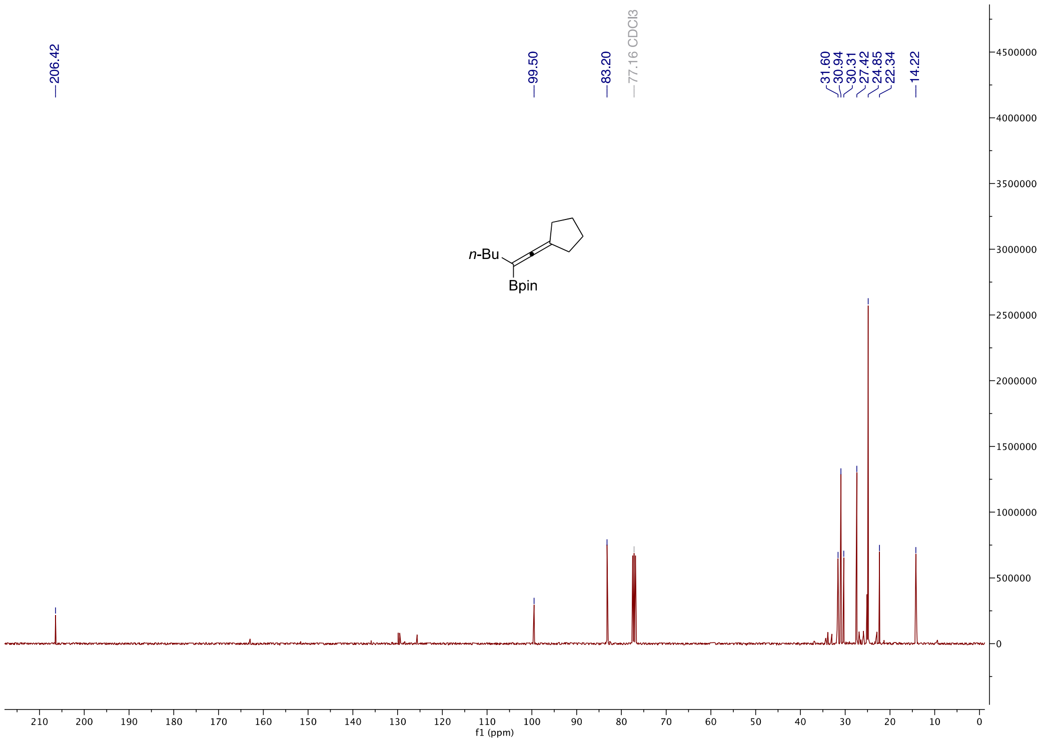

Top: ^{1}H NMR (400 MHz, $CDCl_3$), and bottom: ^{13}C NMR (101 MHz, $CDCl_3$).

2-(1-Cyclopentylidenehept-1-en-2-yl)-4,4,5,5-tetramethyl-1,3,2-dioxaborolane (**2e**)

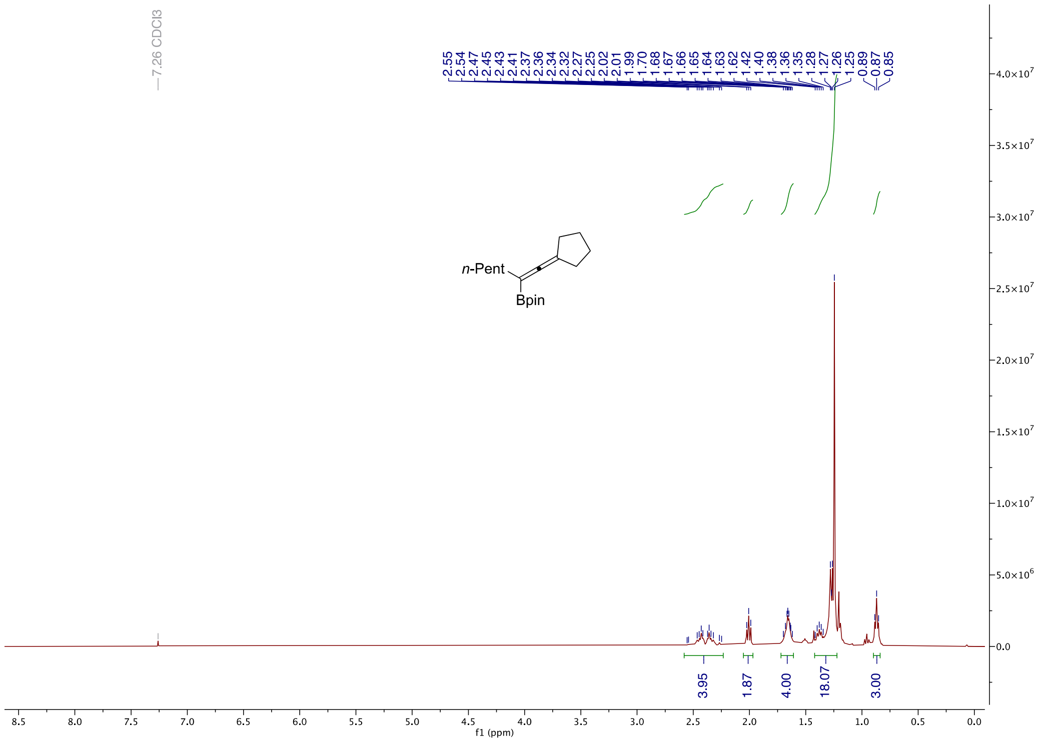

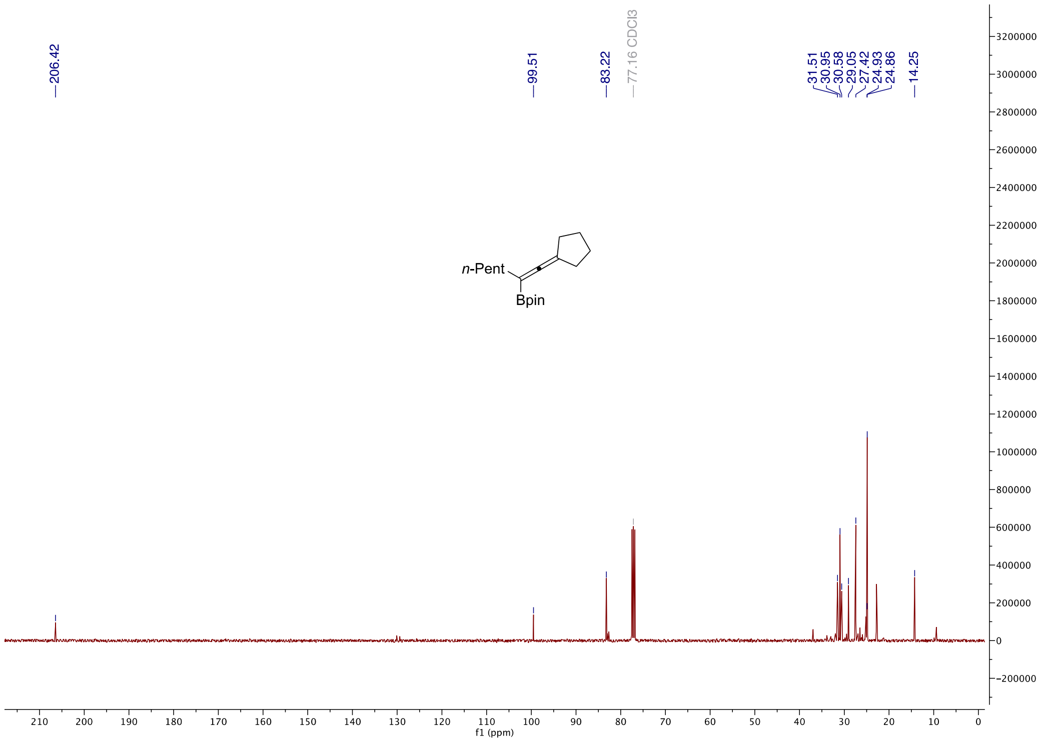

Top: ^{1}H NMR (400 MHz, $CDCl_3$), and bottom: ^{13}C NMR (101 MHz, $CDCl_3$).

2-(1-Cyclohexylidenehept-1-en-2-yl)-4,4,5,5-tetramethyl-1,3,2-dioxaborolane (**2f**)

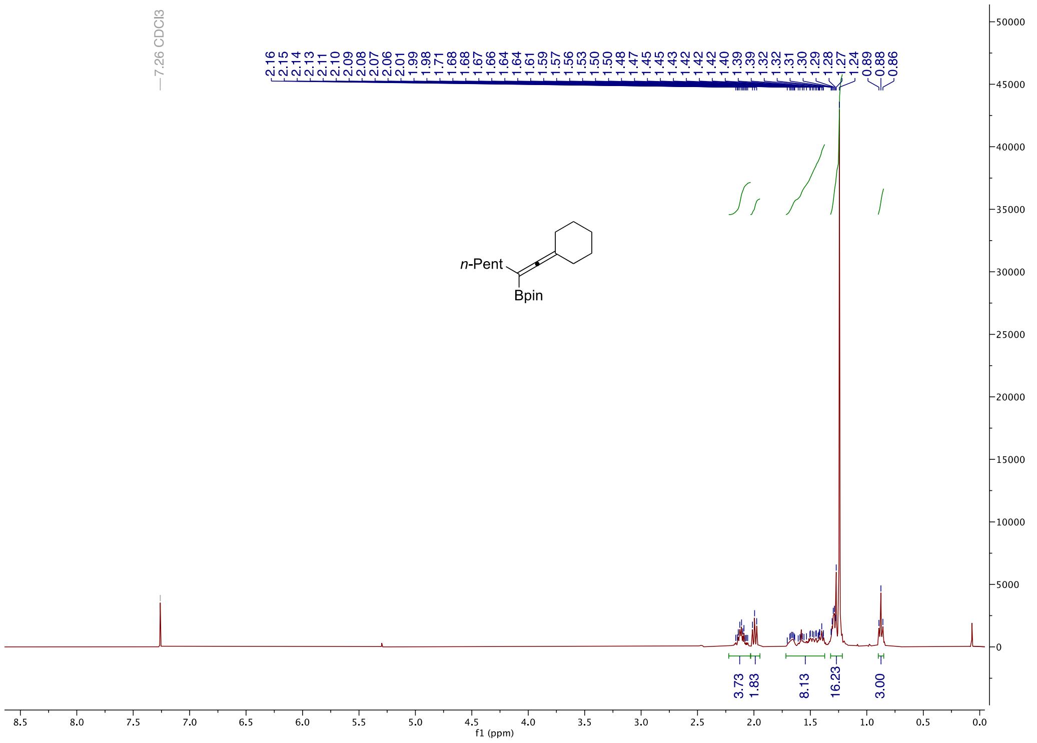

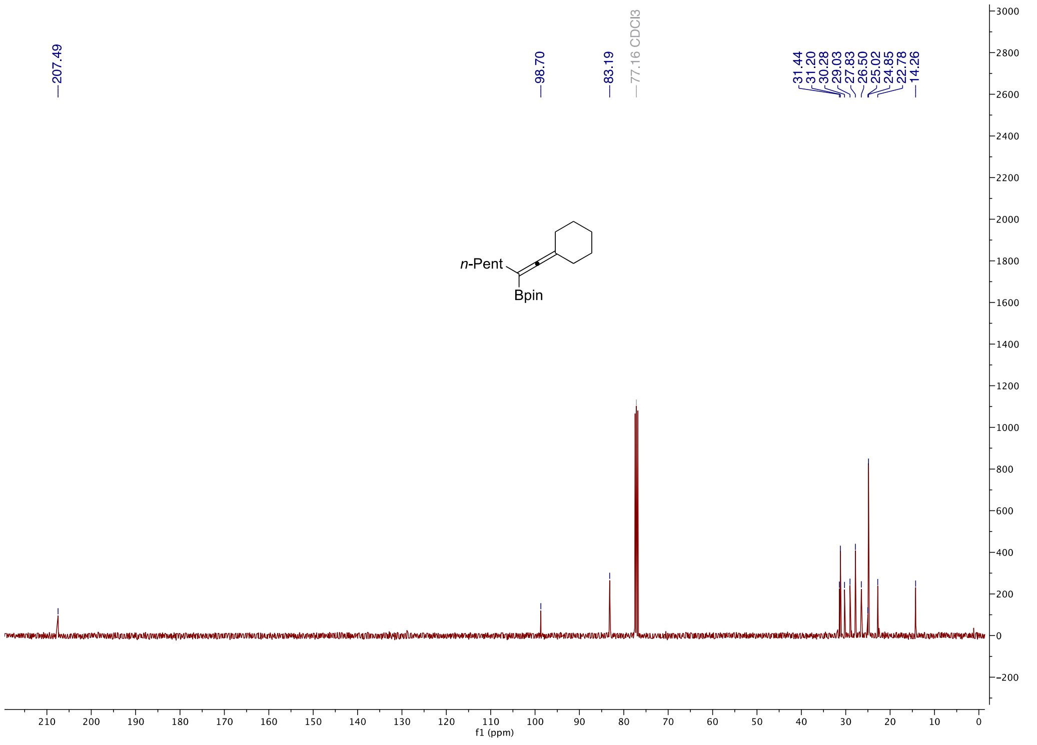

Top: ^{1}H NMR (400 MHz, $CDCl_3$), and bottom: ^{13}C NMR (101 MHz, $CDCl_3$).

tert-Butyldimethyl((4-methyl-6-phenyl-2-(4,4,5,5-tetramethyl-1,3,2-dioxaborolan-2-yl)hexa-2,3-dien-1-yl)oxy)silane (**2g**)

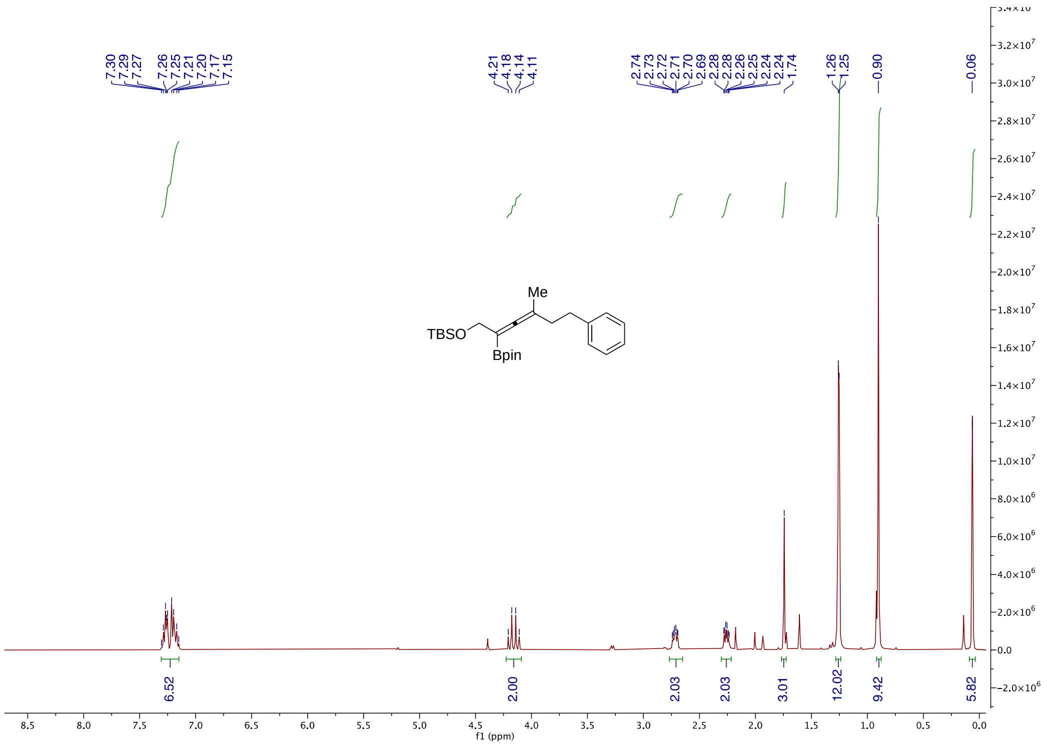

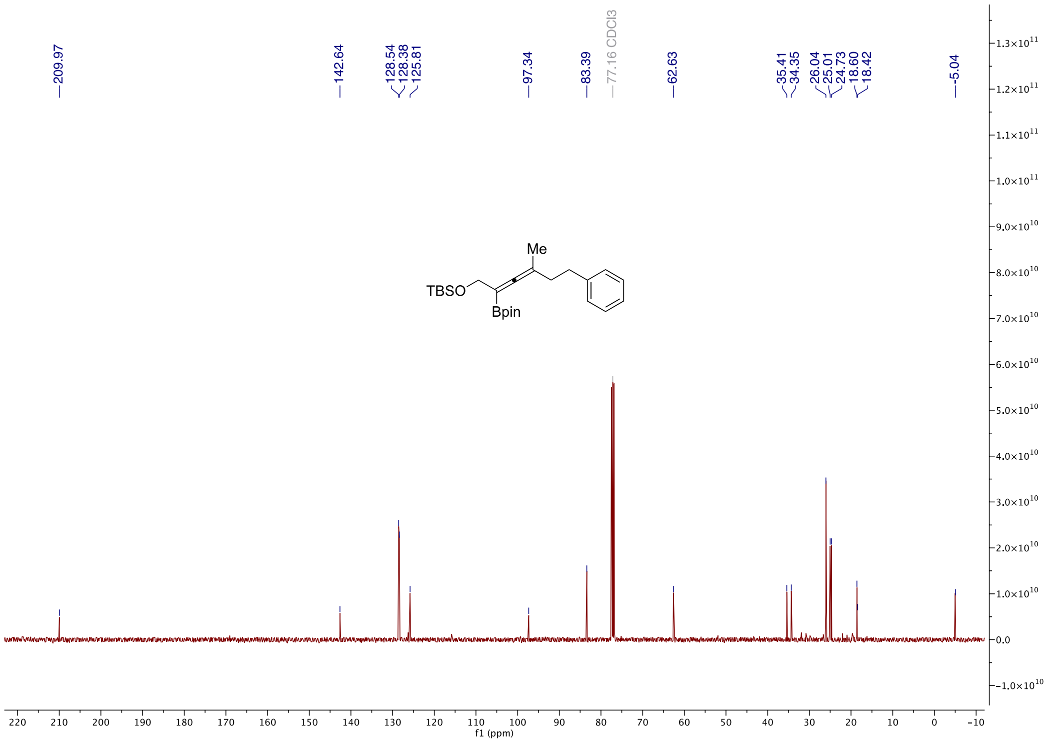

Top: ^{1}H NMR (400 MHz, $CDCl_3$), and bottom: ^{13}C NMR (101 MHz, $CDCl_3$).

tert-Butyl((3-cyclohexylidene-2-(4,4,5,5-tetramethyl-1,3,2-dioxaborolan-2-yl)allyl)oxy)dimethylsilane (**2h**)

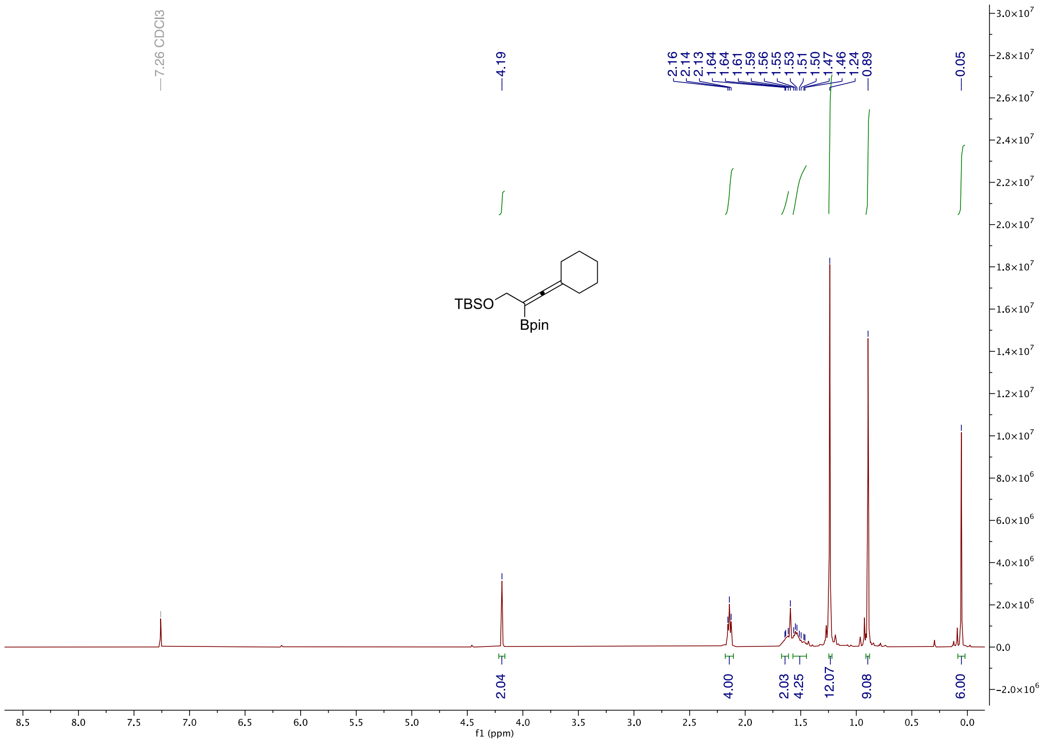

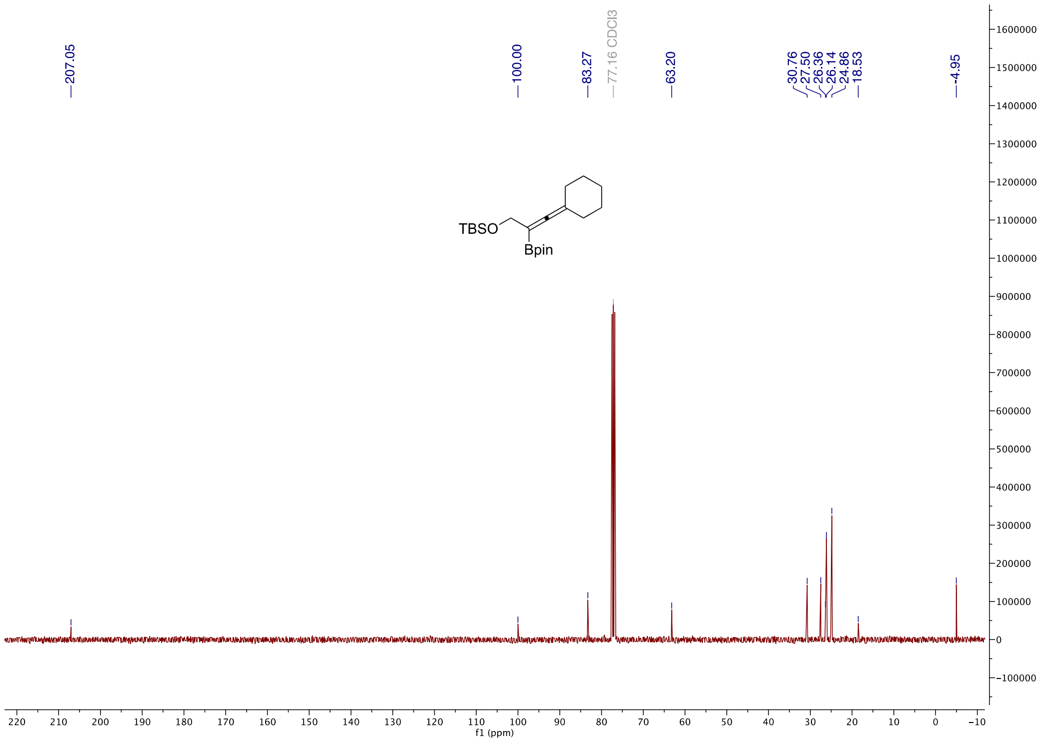

Top: ^{1}H NMR (400 MHz, $CDCl_3$), and bottom: ^{13}C NMR (101 MHz, $CDCl_3$).

tert-Butyldimethyl((5-methyl-7-phenyl-3-(4,4,5,5-tetramethyl-1,3,2-dioxaborolan-2-yl)hepta-3,4-dien-1-yl)oxy)silane (**2i**)

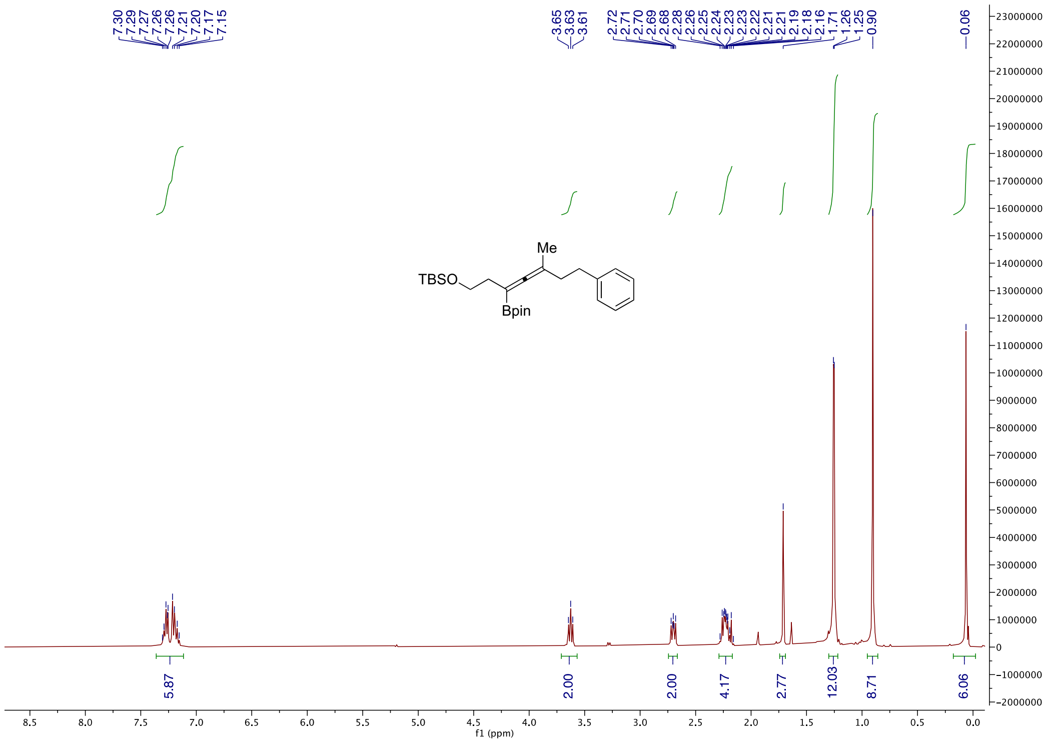

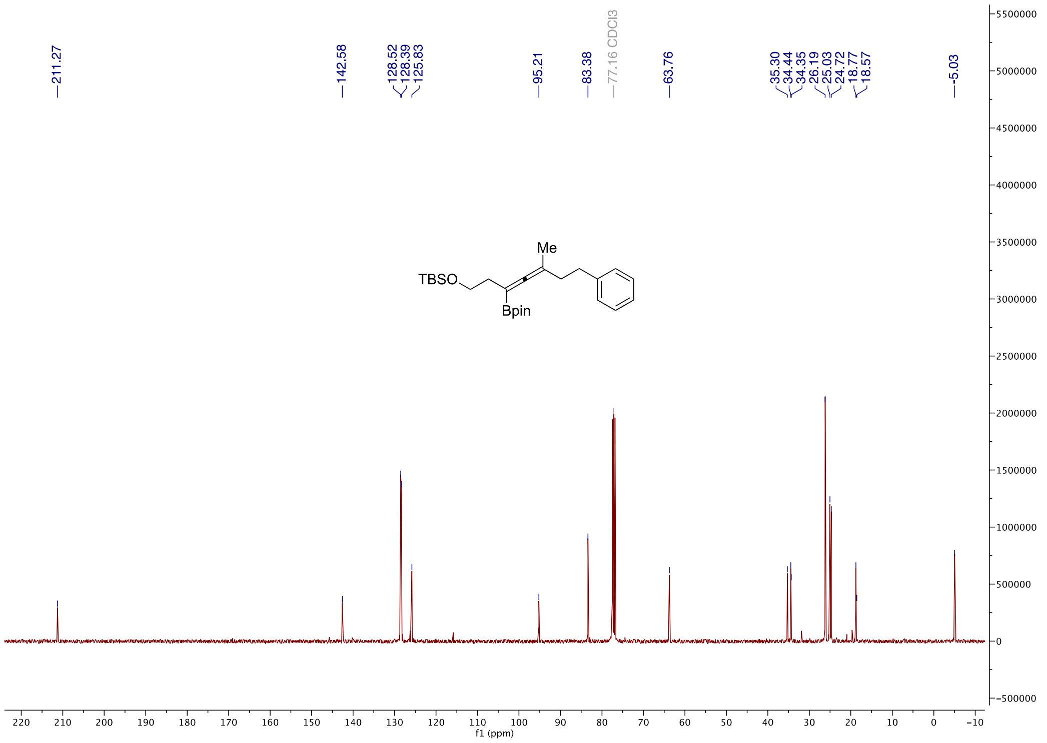

Top: ^{1}H NMR (400 MHz, $CDCl_3$), and bottom: ^{13}C NMR (101 MHz, $CDCl_3$).

tert-Butyl-2,2-bis(((tert-butyldimethylsilyl)oxy)methyl)-3-methyl-5-phenyl-5-(4,4,5,5-tetramethyl-1,3,2-dioxaborolan-2-yl)penta-3,4-dienoate (**2j**)

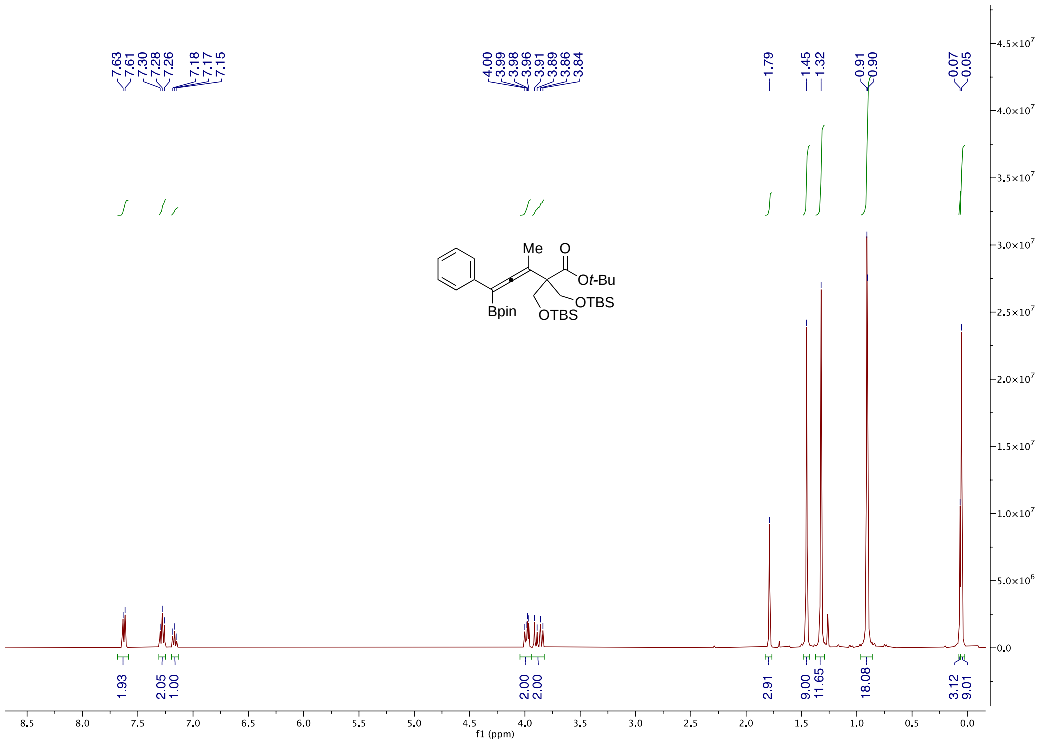

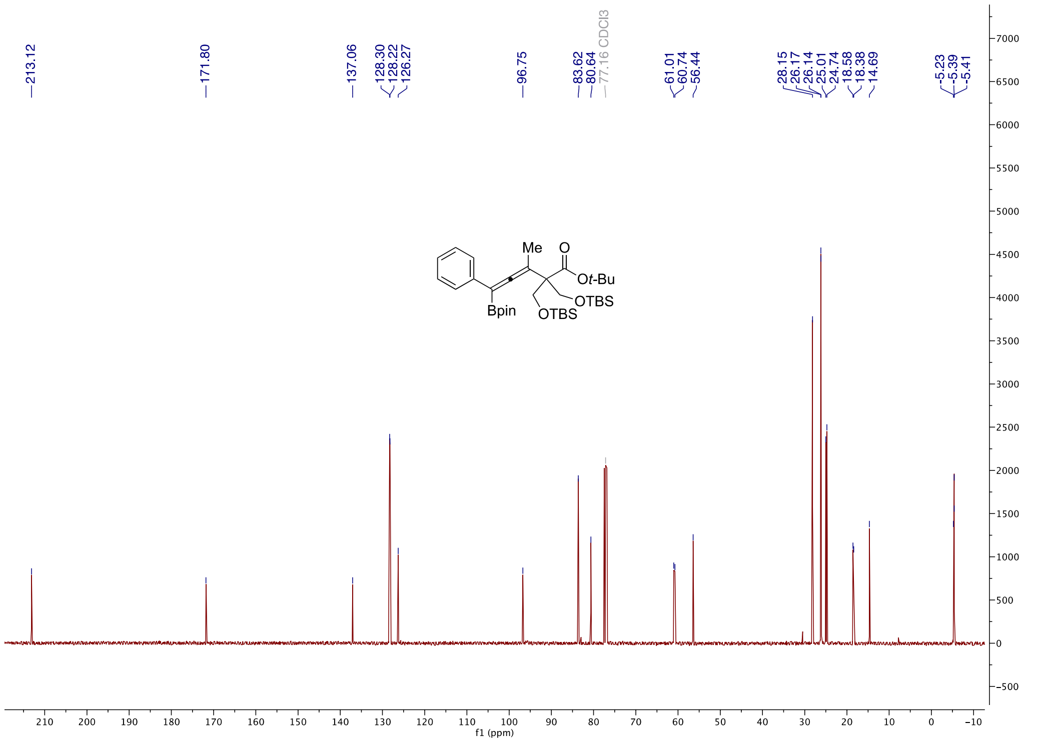

Top: ^{1}H NMR (400 MHz, $CDCl_3$), and bottom: ^{13}C NMR (101 MHz, $CDCl_3$).

*4,4,5,5-Tetramethyl-2-(3-methylbuta-1,2-dien-1-yl)-1,3,2-*dioxaborolane (**2k**)

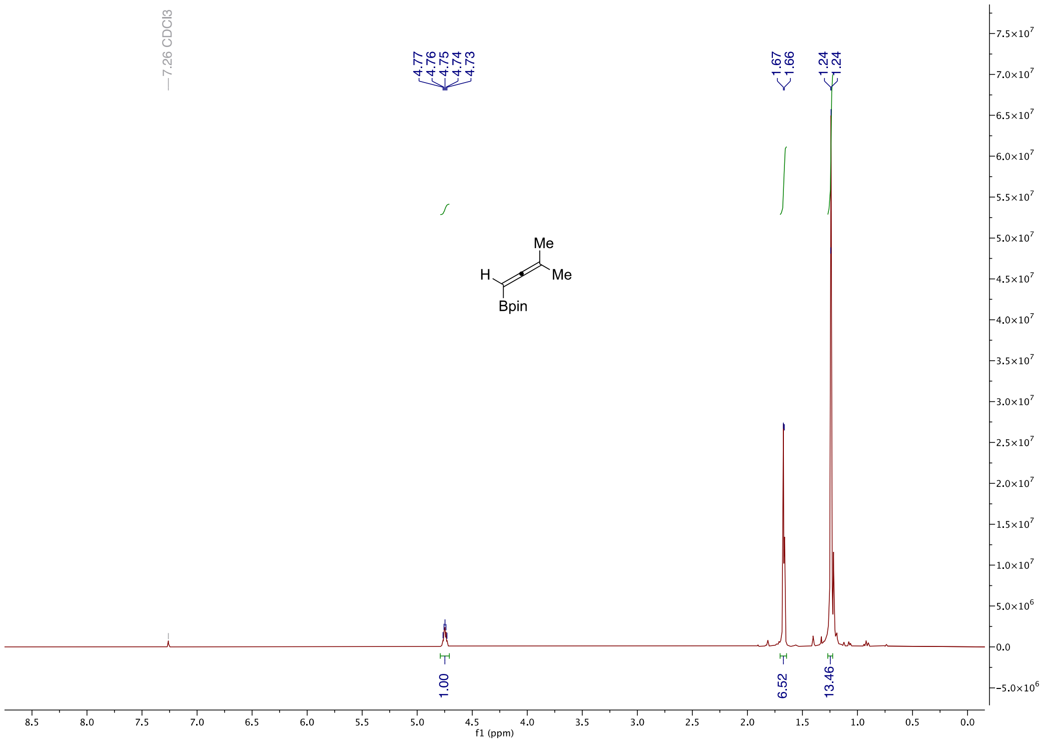

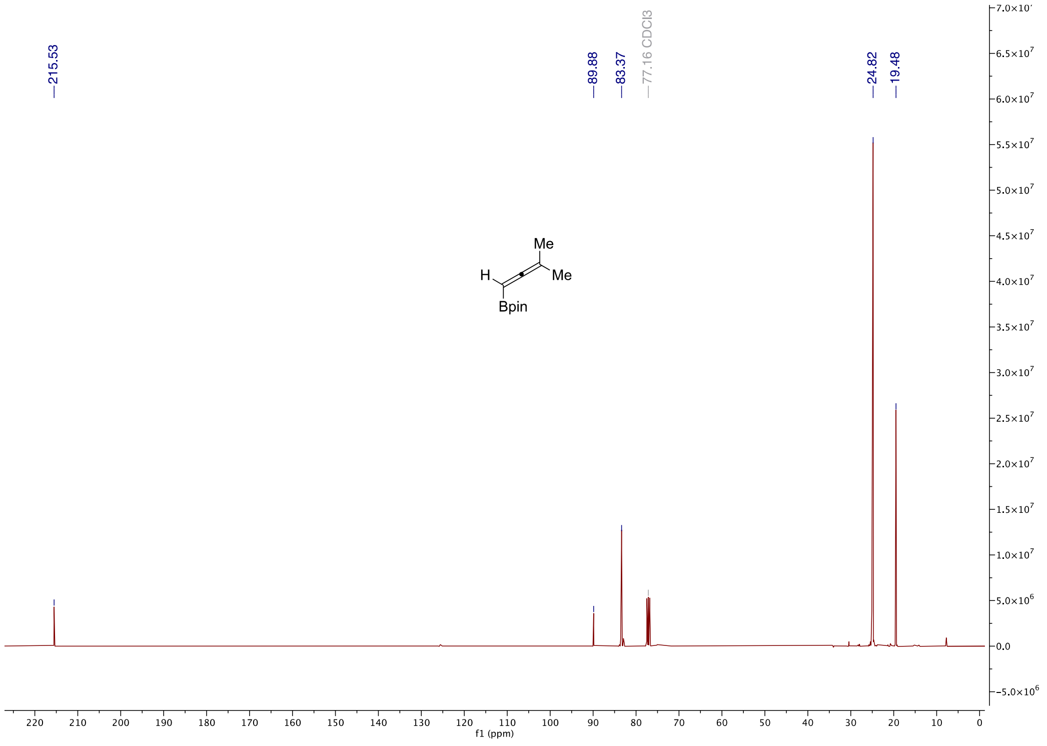

Top: ^{1}H NMR (400 MHz, $CDCl_3$), and bottom: ^{13}C NMR (101 MHz, $CDCl_3$).

2-(3-Ethylpenta-1,2-dien-1-yl)-4,4,5,5-tetramethyl-1,3,2-dioxaborolane (**2l**)

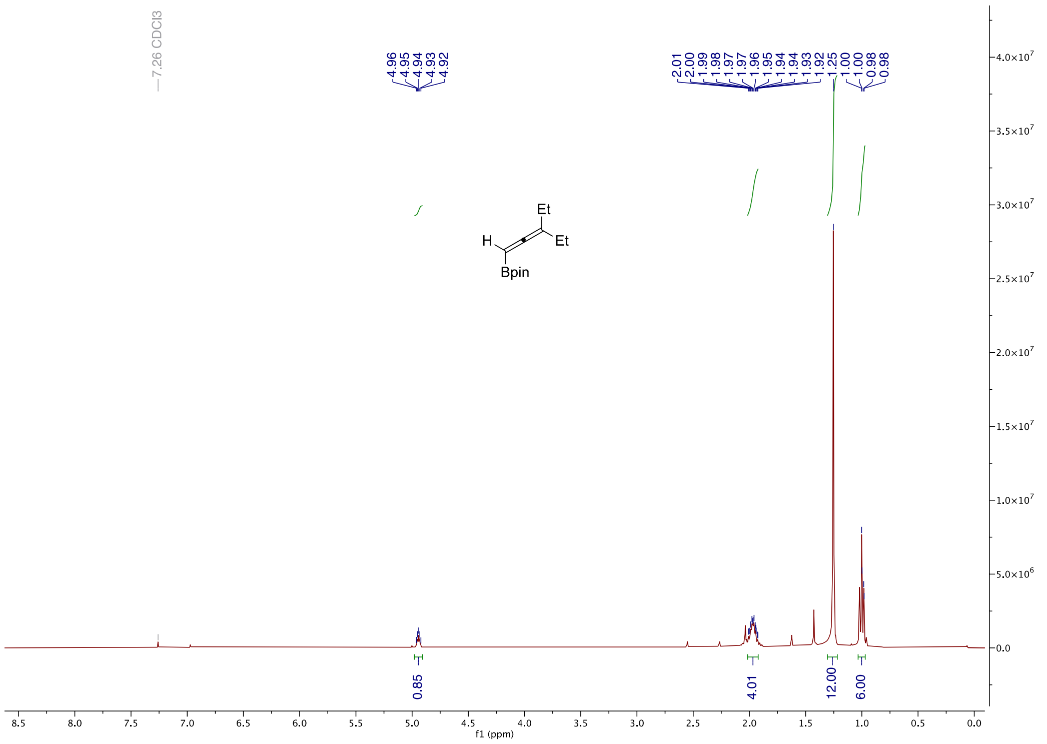

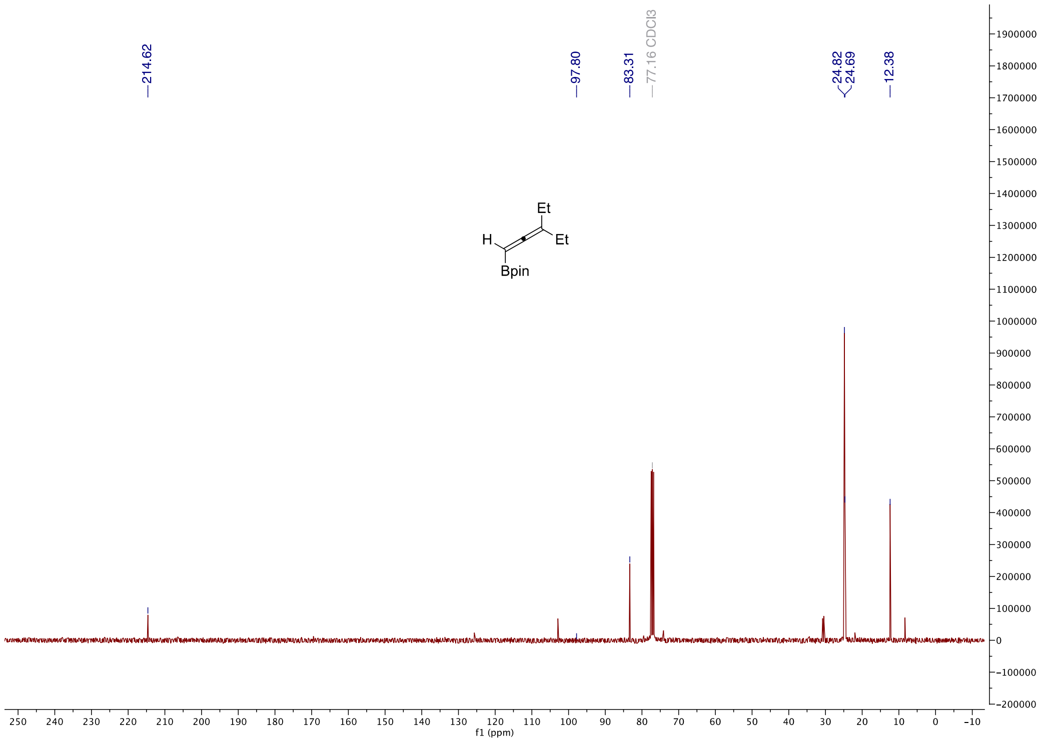

Top: ^{1}H NMR (400 MHz, $CDCl_3$), and bottom: ^{13}C NMR (101 MHz, $CDCl_3$).

2-(2-Cyclohexylidenevinyl)-4,4,5,5-tetramethyl-1,3,2-dioxaborolane (**2m**)

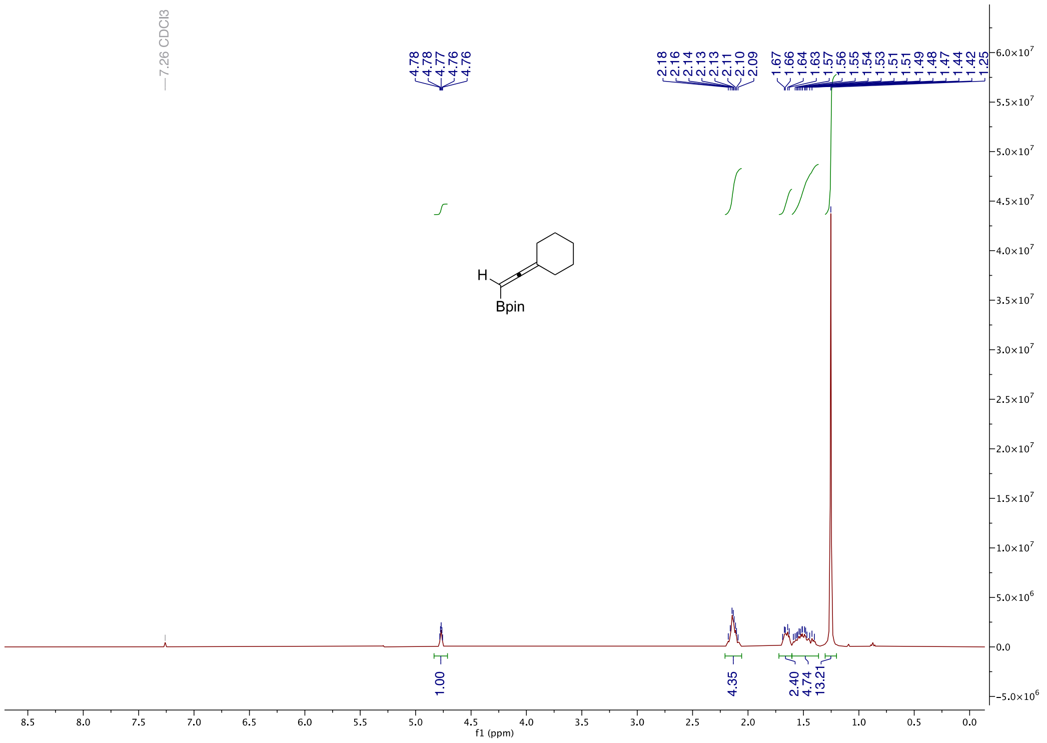

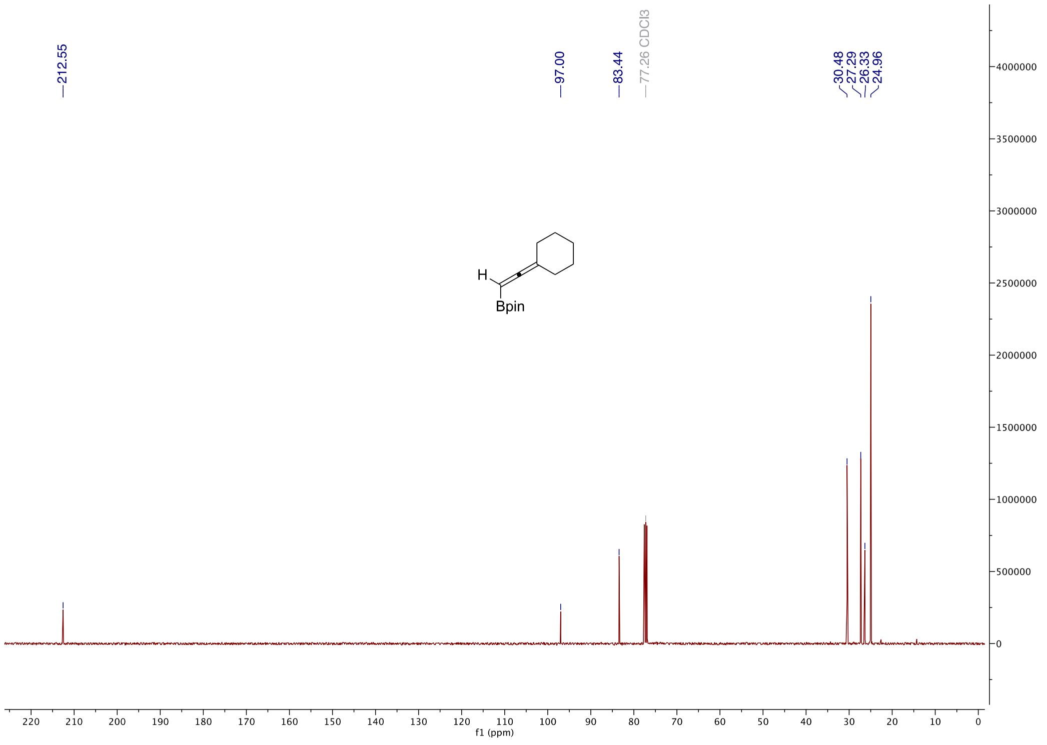

Top: ^{1}H NMR (400 MHz, $CDCl_3$), and bottom: ^{13}C NMR (101 MHz, $CDCl_3$).

4,4,5,5-Tetramethyl-2-(1-phenylbuta-1,2-dien-1-yl)-1,3,2-dioxaborolane (**2n**)

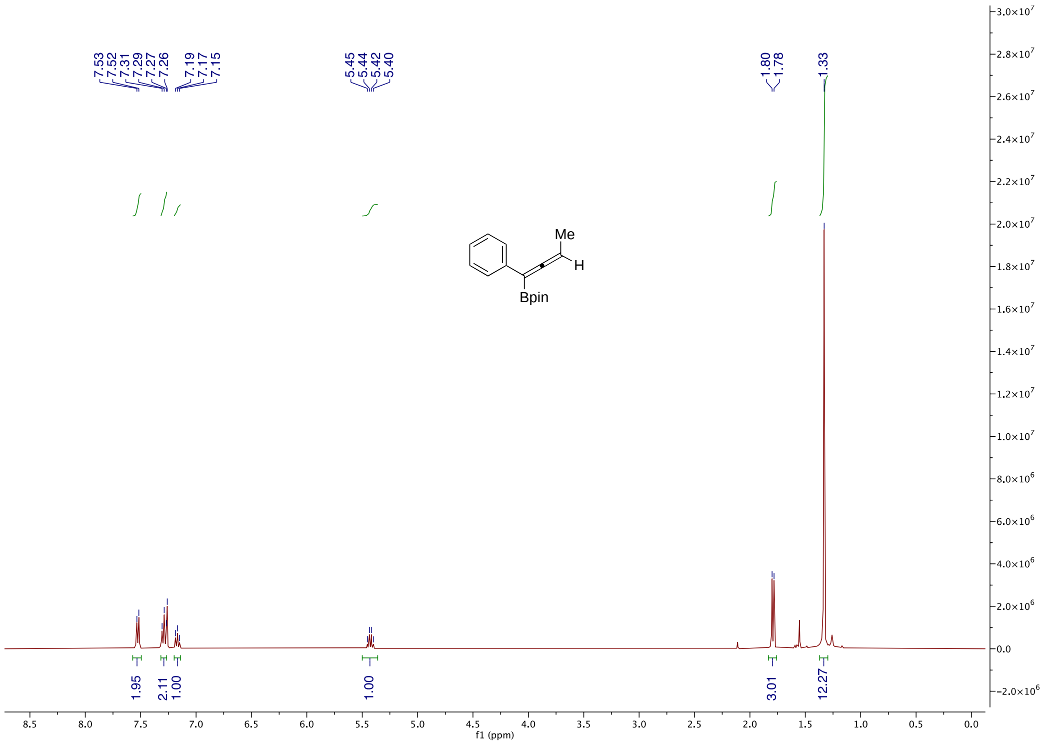

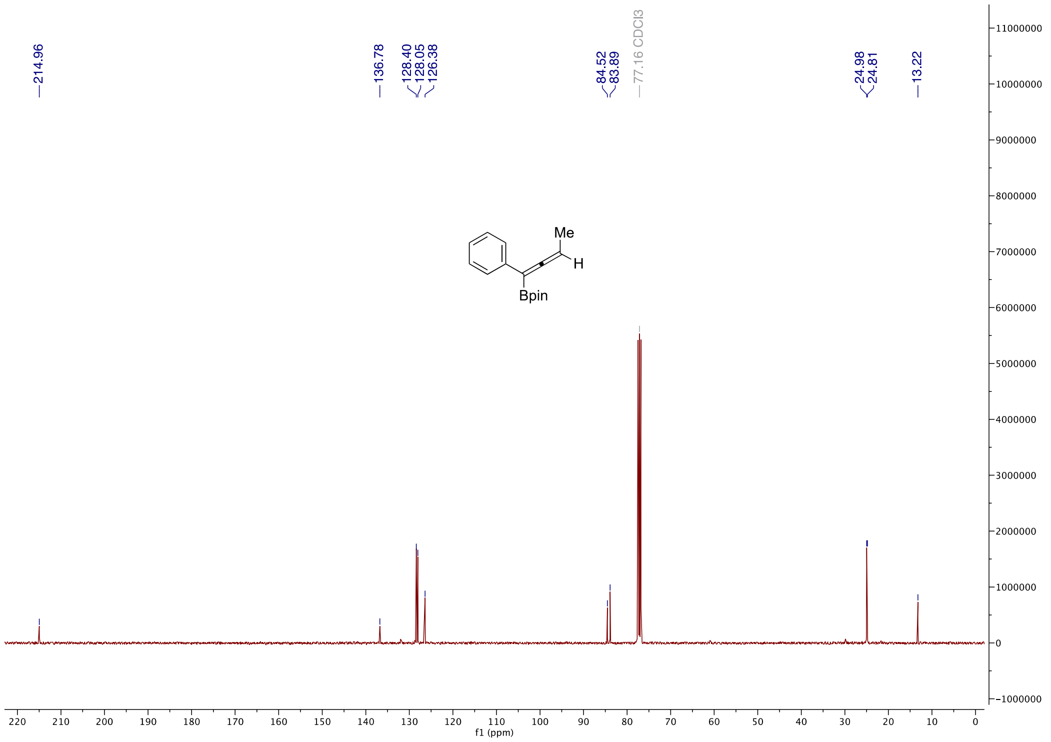

Top: ^{1}H NMR (400 MHz, $CDCl_3$), and bottom: ^{13}C NMR (101 MHz, $CDCl_3$).

4,4,5,5-Tetramethyl-2-(1-phenylpenta-1,2-dien-1-yl)-1,3,2-dioxaborolane (**2o**)

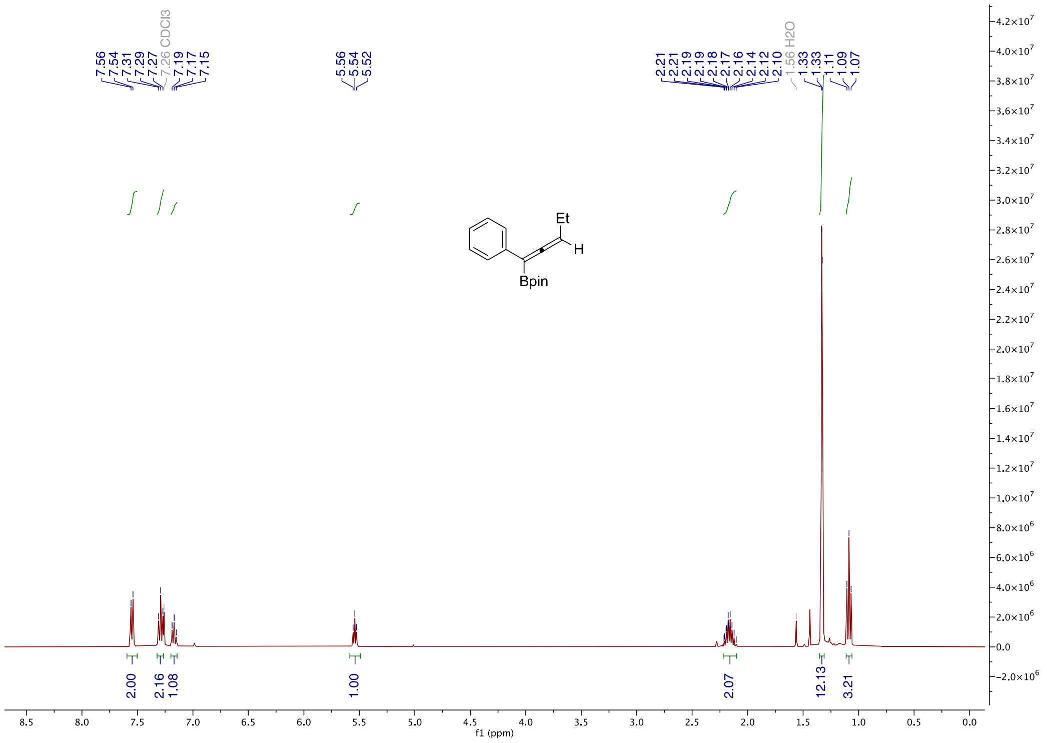

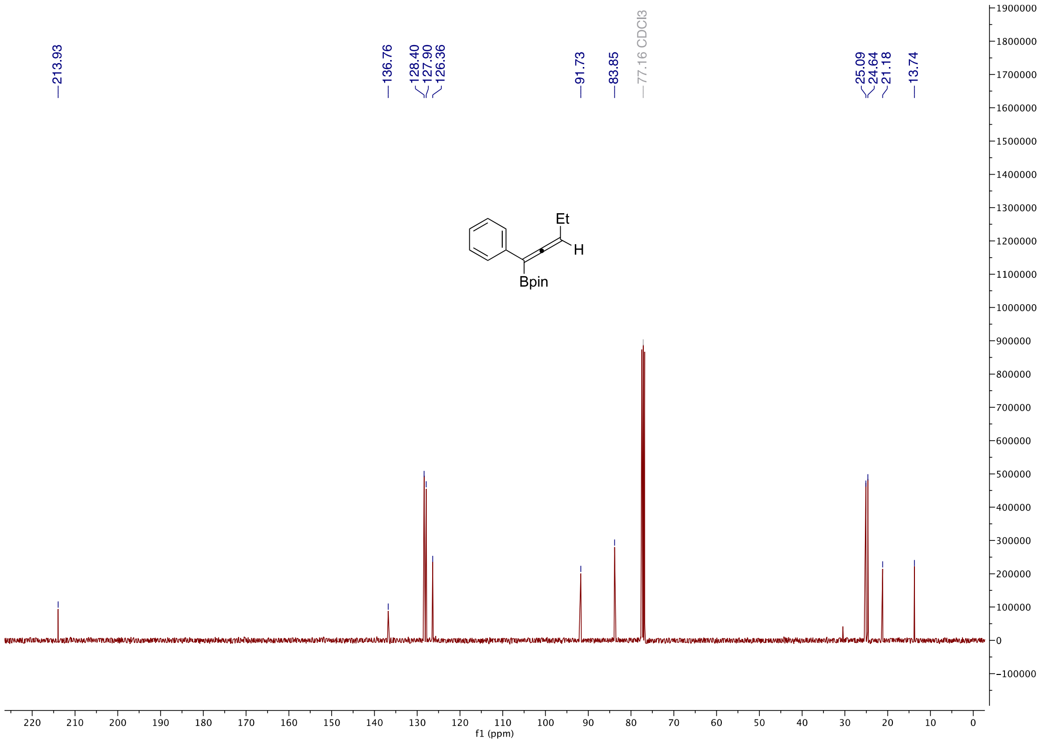

Top: ^{1}H NMR (400 MHz, $CDCl_3$), and bottom: ^{13}C NMR (101 MHz, $CDCl_3$).

4,4,5,5-Tetramethyl-2-(3-phenylbuta-1,2-dien-1-yl)-1,3,2-dioxaborolane (**2p**)

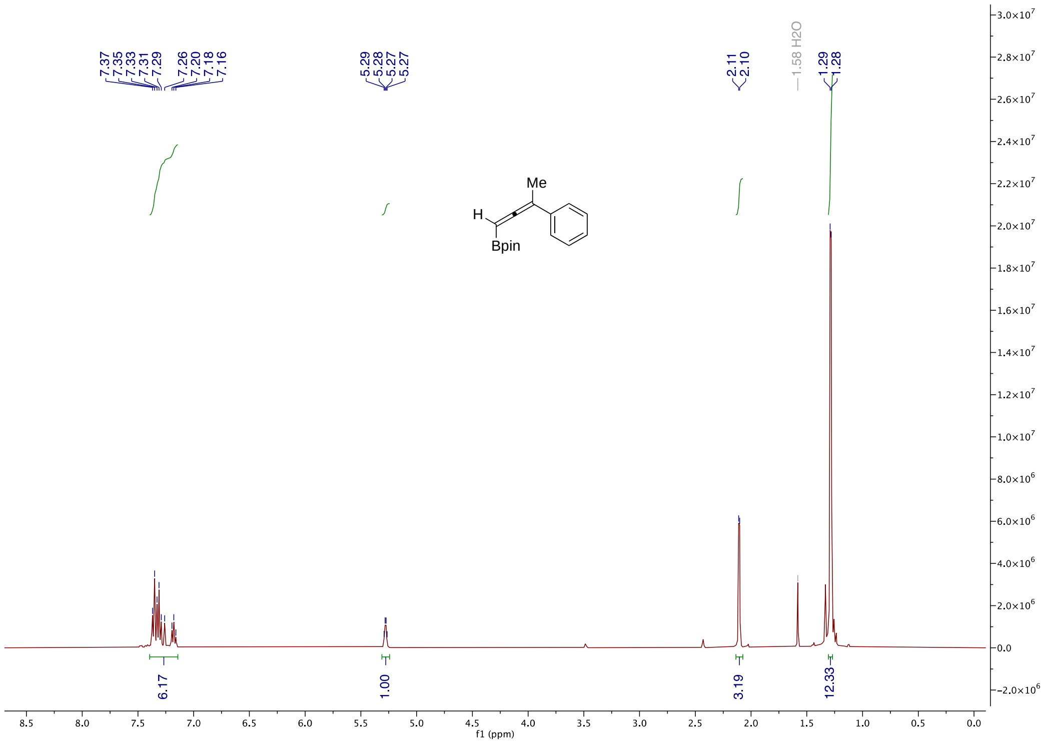

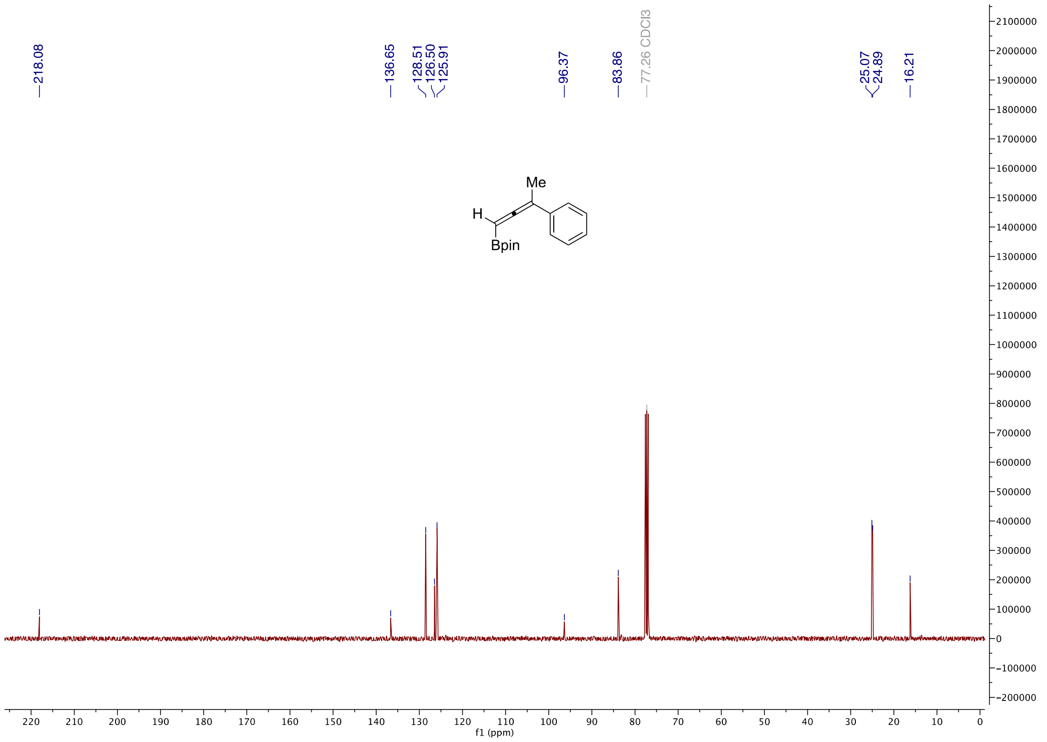

Top: ^{1}H NMR (400 MHz, $CDCl_3$), and bottom: ^{13}C NMR (101 MHz, $CDCl_3$).

tert-Butyldimethyl((2-methyl-4-(4,4,5,5-tetramethyl-1,3,2-dioxaborolan-2-yl)buta-2,3-dien-1-yl)oxy)silane (**2q**)

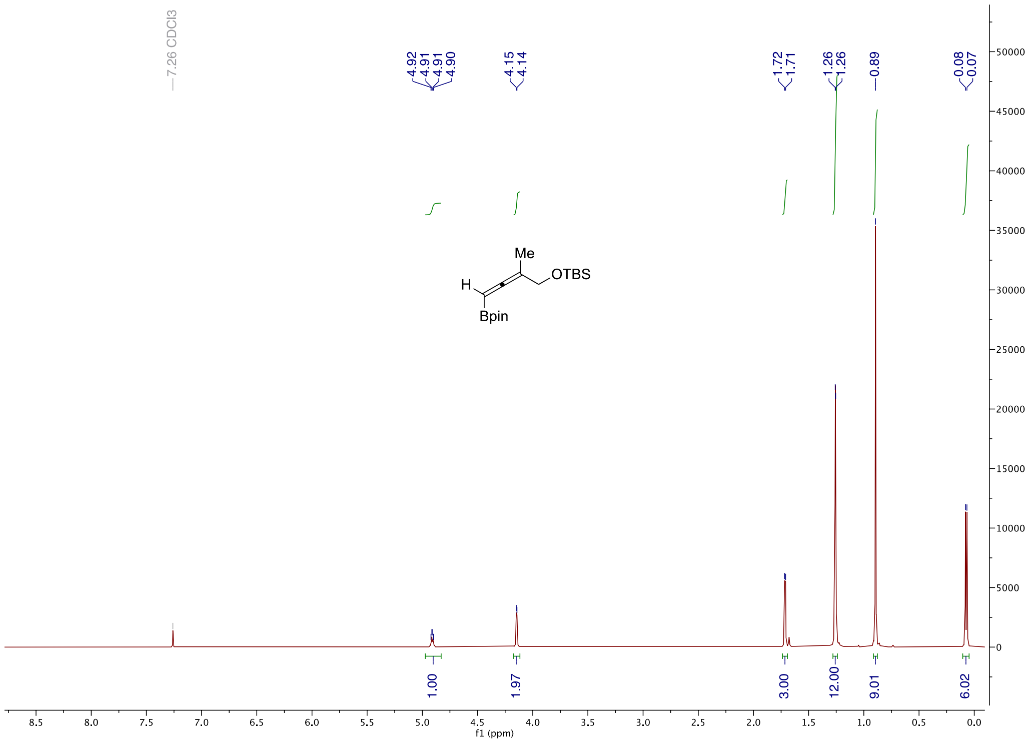

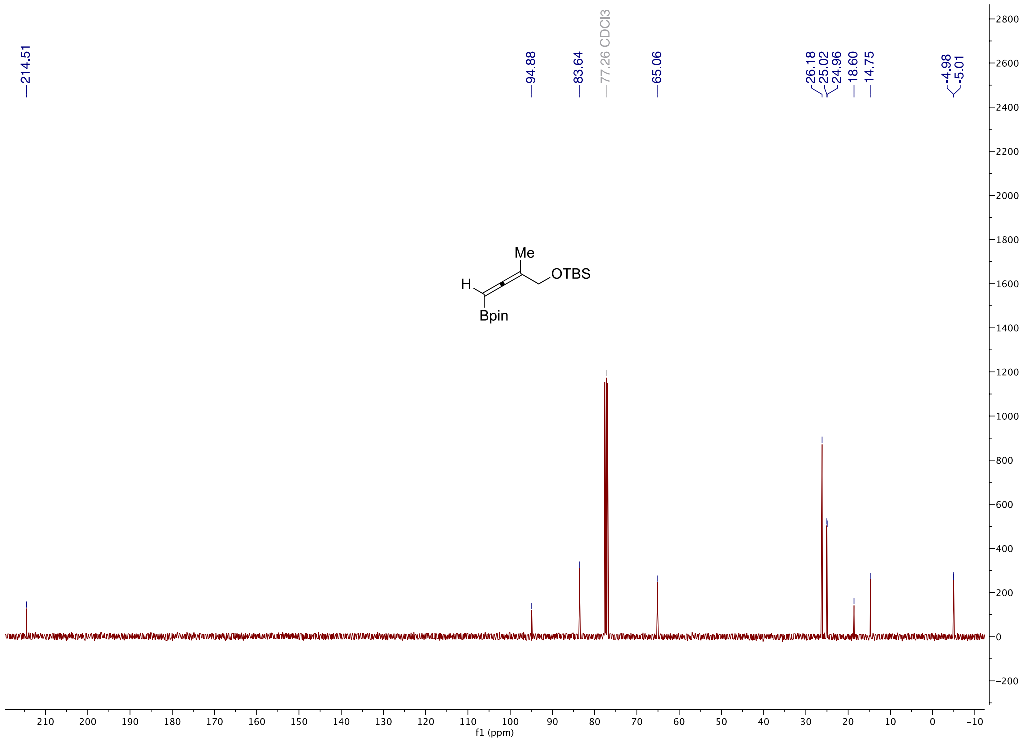

Top: ^{1}H NMR (400 MHz, $CDCl_3$), and bottom: ^{13}C NMR (101 MHz, $CDCl_3$).

4,4,5,5-Tetramethyl-2-(octa-1,2-dien-1-yl)-1,3,2-dioxaborolane (**2r**)

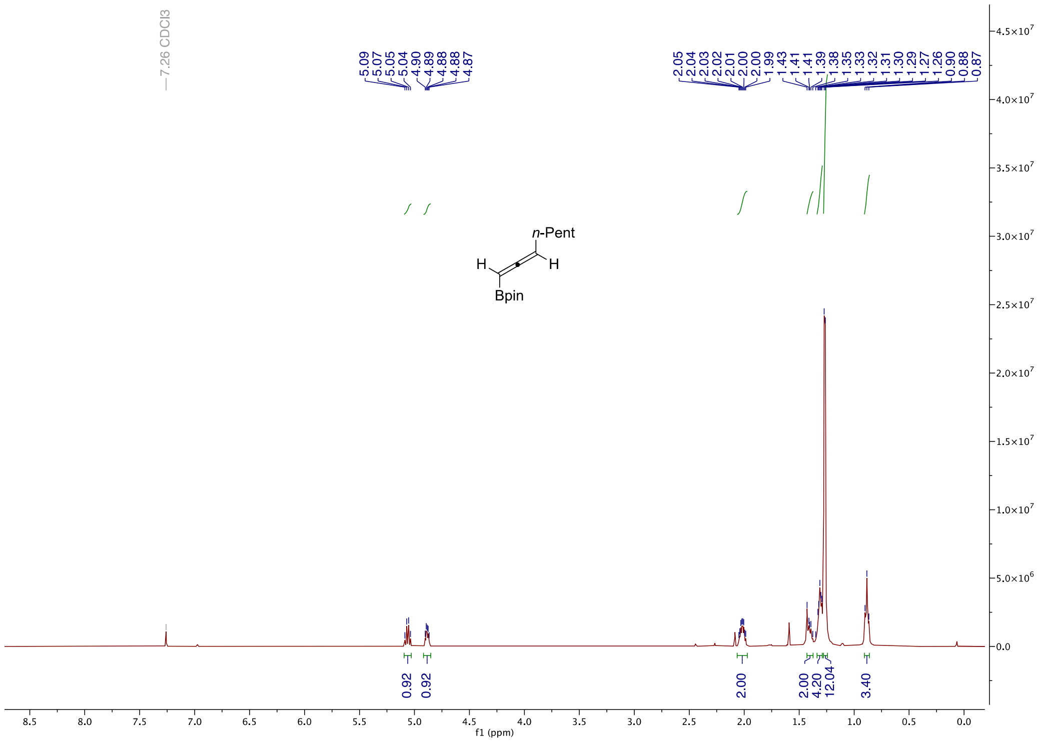

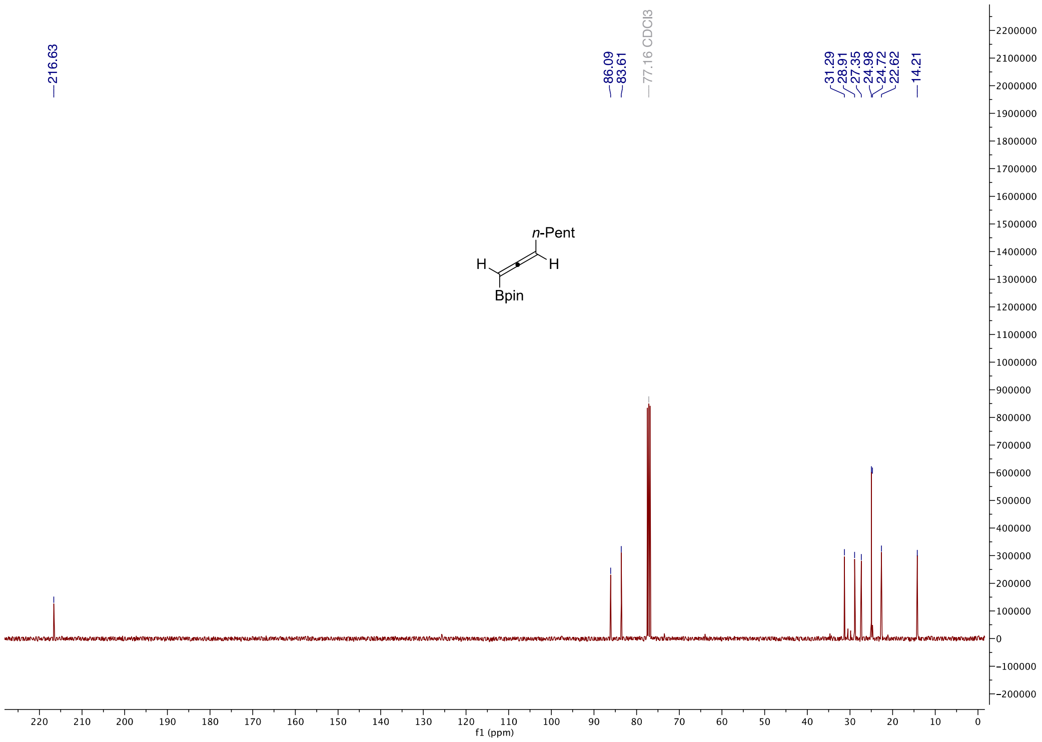

Top: ^{1}H NMR (400 MHz, $CDCl_3$), and bottom: ^{13}C NMR (101 MHz, $CDCl_3$).

(3S,8S,10R,13S,14S)-17-(3-((tert-Butyldimethylsilyl)oxy)-2-(4,4,5,5-tetramethyl-1,3,2-dioxaborolan-2-yl)prop-1-en-1-ylidene)-10,13-dimethyl-2,3,4,7,8,9,10,11,12,13,14,15,16,17-tetradecahydro-1H-cyclopenta[a]phenanthren-3-yl acetate (**2s**)

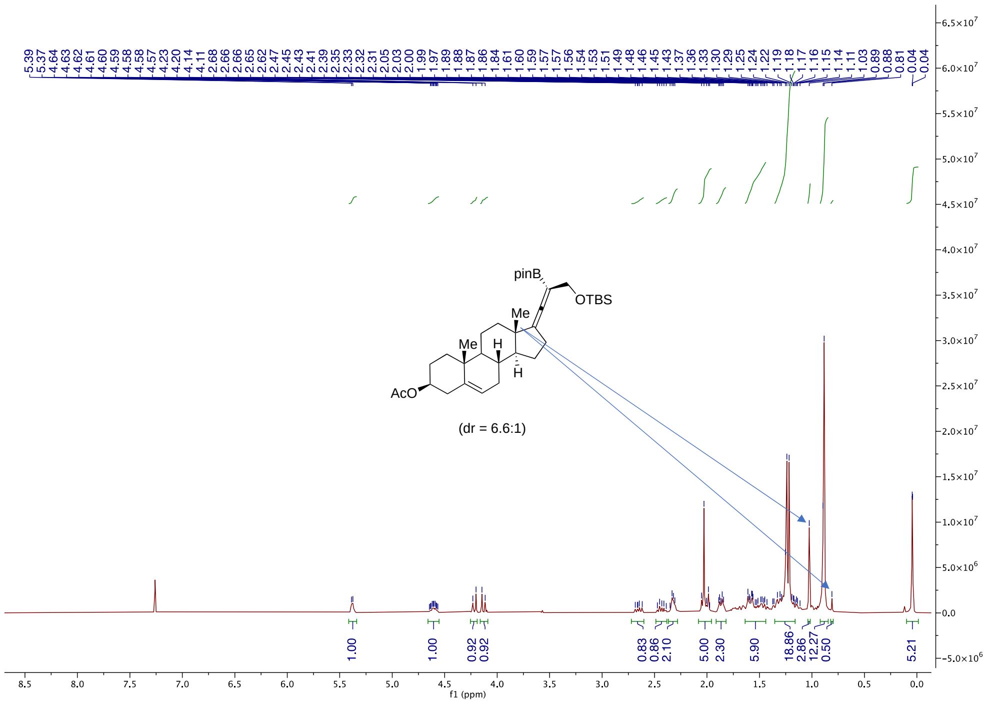

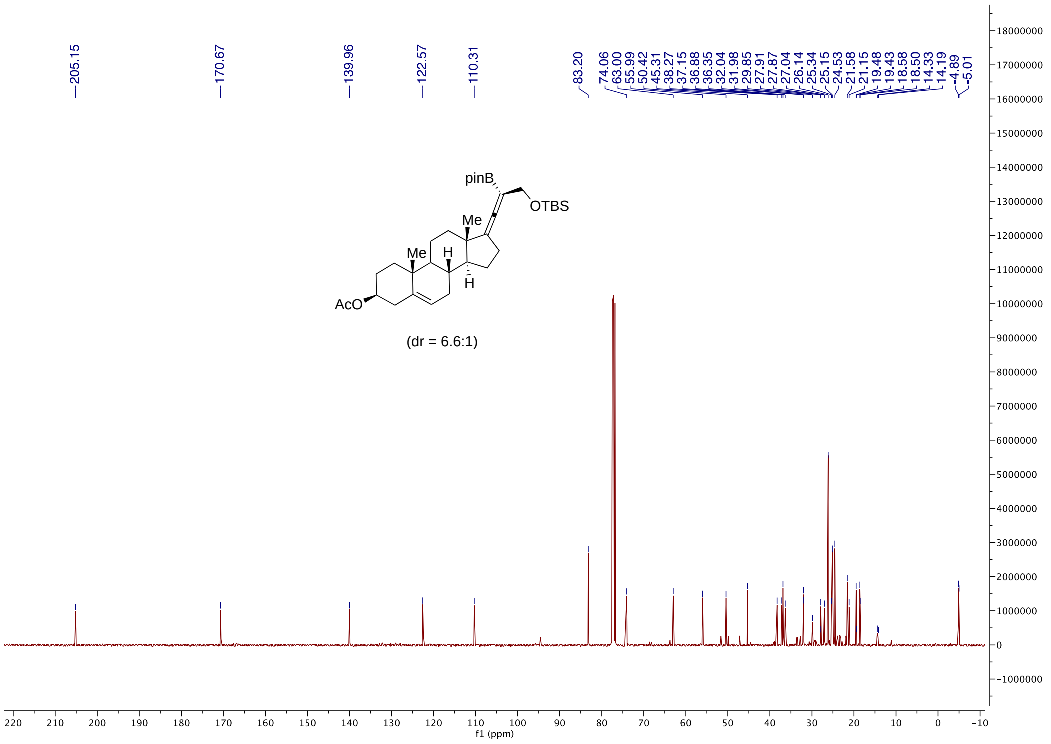

Top: ^{1}H NMR (400 MHz, $CDCl_3$), and bottom: ^{13}C NMR (101 MHz, $CDCl_3$).

3-Methyl-1-phenylbut-2-en-1-one (**3**)

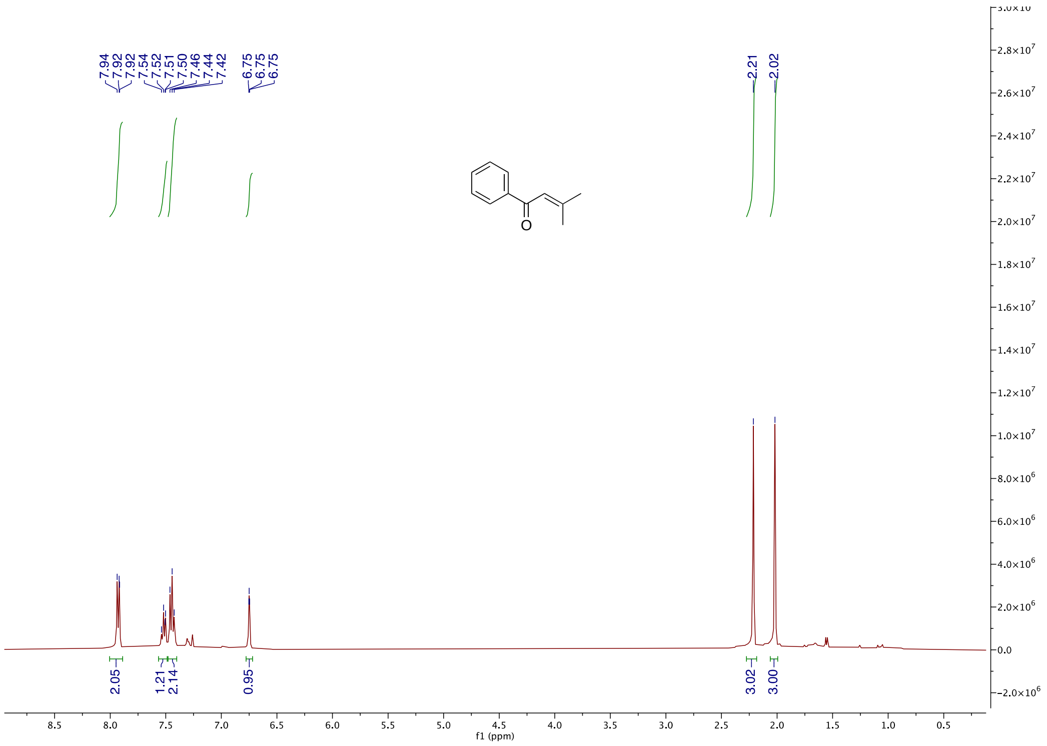

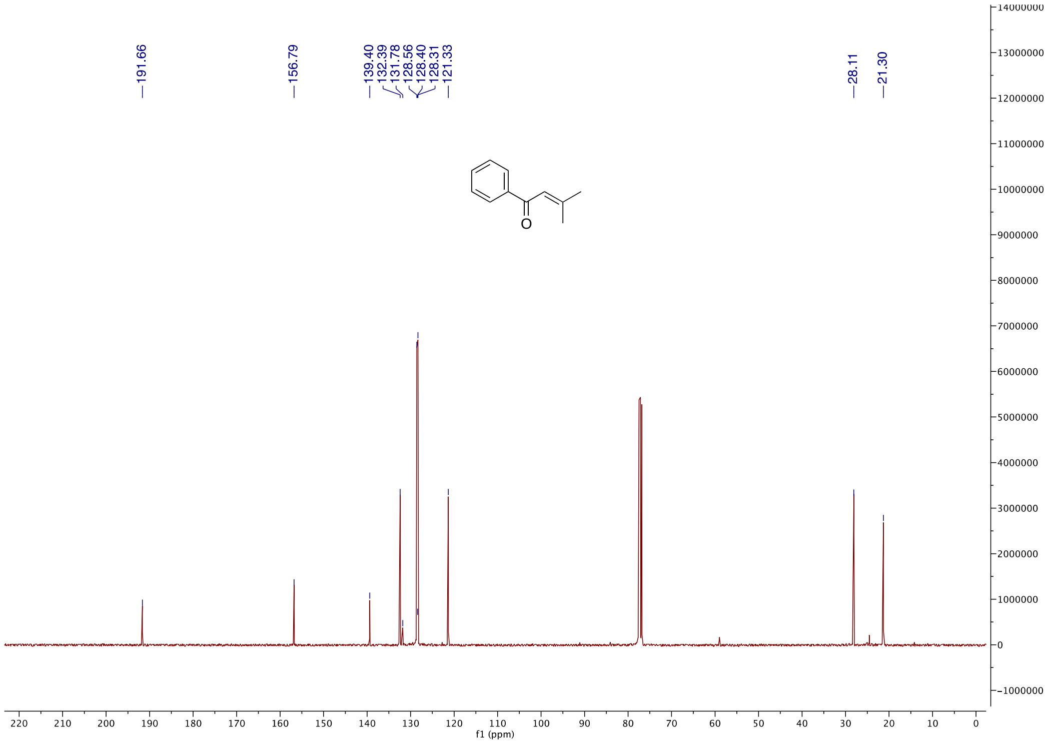

Top: ^{1}H NMR (400 MHz, $CDCl_3$), and bottom: ^{13}C NMR (101 MHz, $CDCl_3$).

Trifluoro(3-methyl-1-phenylbuta-1,2-dien-1-yl)-λ4-borane, potassium salt (**4**)

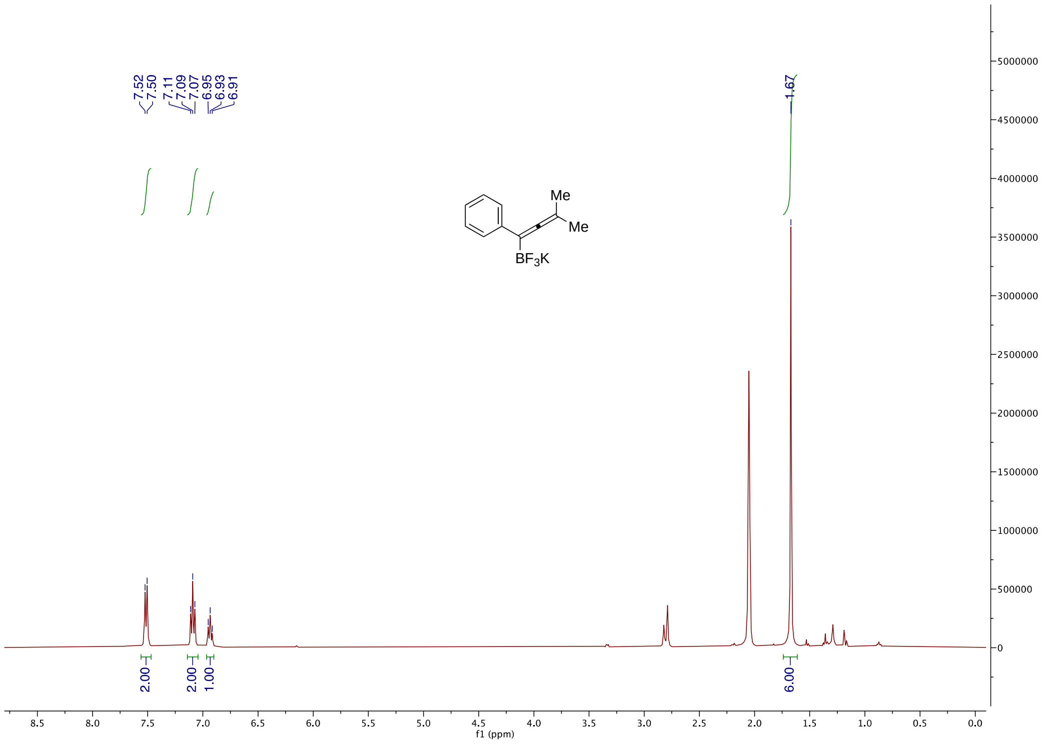

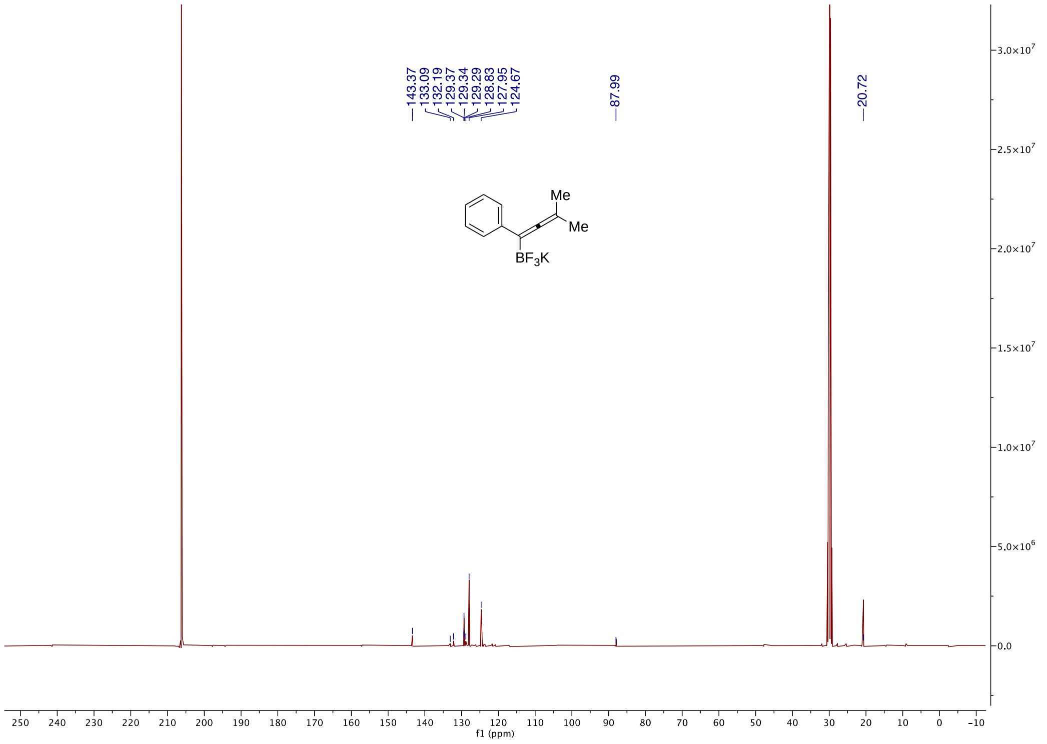

Top: ^{1}H NMR (400 MHz, COCd$_6$), and bottom: ^{13}C NMR (101 MHz, COCD$_6$).

2,2-Dimethyl-4-phenylbut-3-yn-1-ol (**5**)

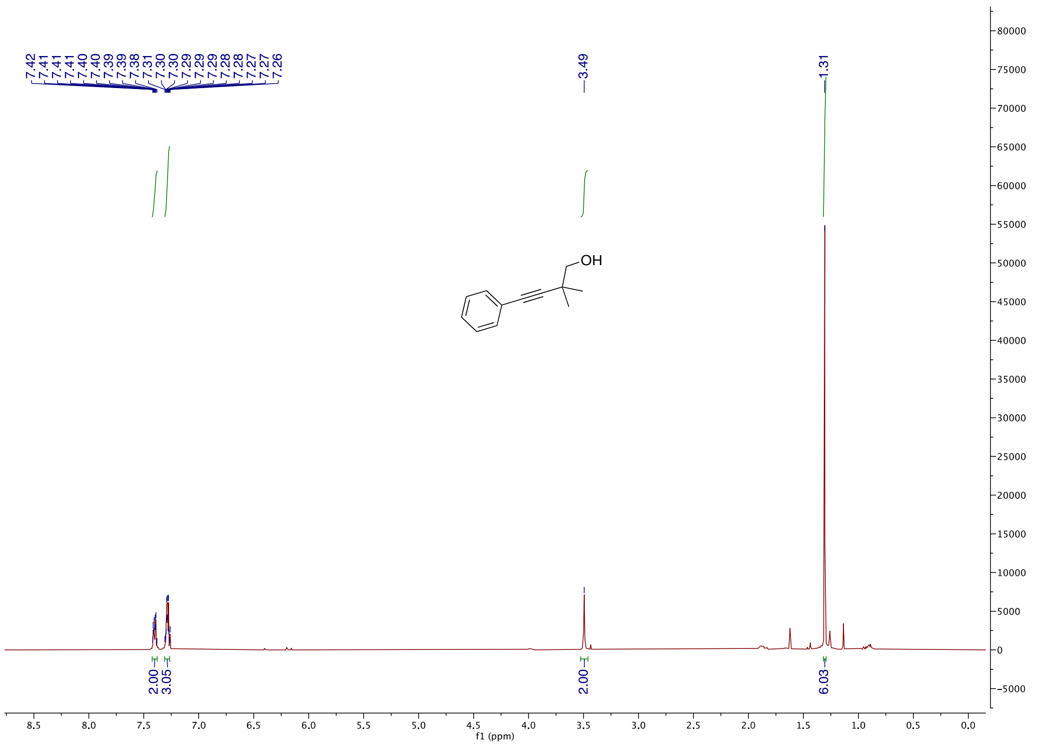

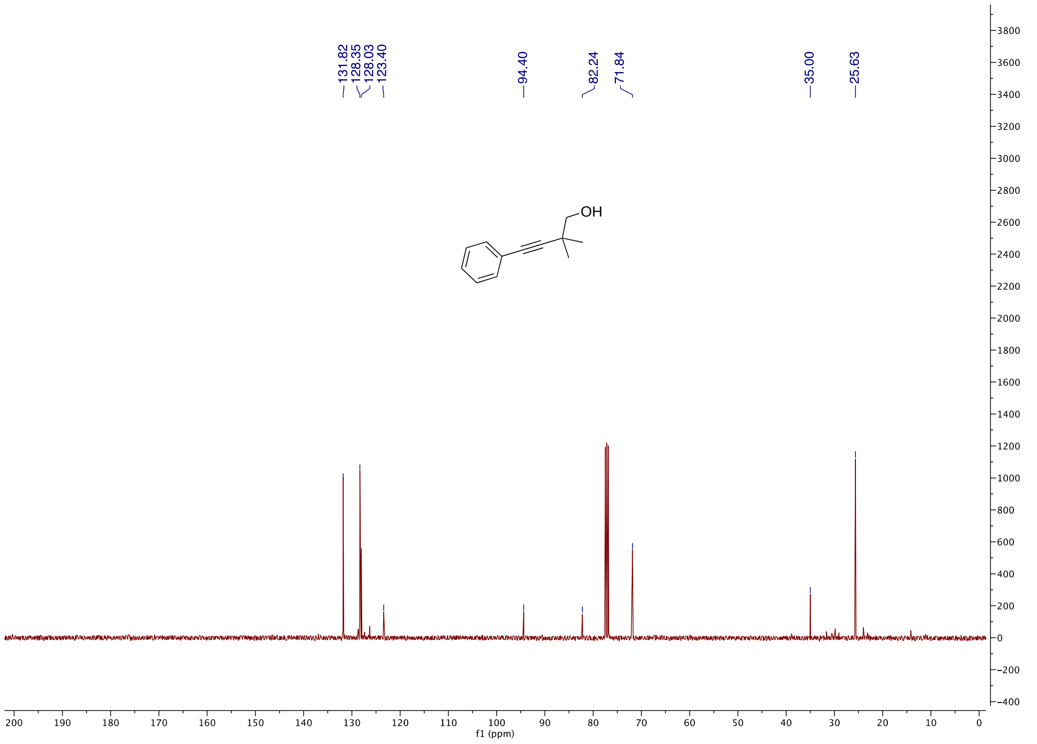

Top: ^{1}H NMR (400 MHz, $CDCl_3$), and bottom: ^{13}C NMR (101 MHz, $CDCl_3$).

6. References

[1] Krasovskiy, A.; Knochel, P. *Synthesis* **2006**, 890
[2] Posevins, D.; Bermejo-López, A. Bäckvall, J.-E. *Angew. Chem. Int. Ed*. **2021**, *60*, 22178–22183
[3] Qiao, H.; Zhang, S.; Li, K.; Cao, Z.; Zeng, F. *J. Org. Chem*. **2019**, *84*, 10843−10851
[4] Kessler, S. N.; Bäckvall, J.-E. *Angew. Chem. Int. Ed*. **2016**, *55*, 3734–3738
[5] Yu, M.; Zhang, G.; Zhang, L. *Org. Lett*. **2007**, *9*, 11, 2147–2150
[6] Alduhaish, O.; Varala, R.; Adil, S. F.; Khan, M.; Siddiqui, M. R. H.; AlAwarthan, A.; Alam, M. M. *J. Chem*. **2020**, 9139648
[7] Pradhan, T. R.; Mohapatra, D. K. *Adv. Synth. Catal*. **2019**, *361*, 3605–3611
[8] Zhao, T. T. N.; Yang, Y.; Lessing, T.; Szabó, K. J. *J. Am. Chem. Soc*. **2014**, *136*, 21, 7563–7566
[9] Kessler, S. N.; Hundemer, F.; Bäckvall, J.-E. *ACS Catal*. **2016**, *6*, 7448–7451
[10] Mao, L.; Szabó, K. J.; T. B. *Org. Lett*. **2017**, *19, 5*, 1204–1207
[11] Koyama, S.; Takahashi, F.; Saito, H.; Yorimitsu, H. *Org. Lett*. **2021**, *23*, 8590–8594
[12] Ito, H.; Sasaki, Y.; Sawamura, M. *J. Am. Chem. Soc*. **2008**, *130*, 47, 15774–15775
[13] Lippincott, D. J.; Lindstadt, R. T. H.; Maser, M. R.; Lipshutz, B. H. *Angew. Chem. Int. Ed*. **2017**, *56*, 847–850
[14] Gao, D.-W.; Xiao, Y.; Liu, M.; Liu, Z.; Karunananda, M. K.; Chen, J. S.; Engle, K. M. *ACS Catal*. **2018**, *8,* 4, 3650–3654
[15] Huang, Y.; del Pozo, J.; Torker, S.; Hoveyda, A. H. *J. Am. Chem. Soc*. **2018**, *140*, 7, 2643–2655
[16] Zhang, X.; Lu, Z.; Fu, C.; Ma, S. Org. *Biomol. Chem. Org.* **2009**, *7*, 3258–3263
[17] Sang, H. L.; Yu, S.; Ge, S. *Org. Chem. Front.* **2018**, *5*, 1284–1287
[18] Shen, Y.; Huang, B.; Zheng, J.; Lin, C.; Liu, Y.; Cui, S. *Org. Lett*. **2017**, 1*9*, 7, 1744–1747
